# The global burden of cardiovascular disease attributable to diet high in sugar-sweetened beverages among people aged 60 years and older: an analysis for the global burden of disease study 2019

**DOI:** 10.3389/fpubh.2024.1366286

**Published:** 2024-07-19

**Authors:** Jiajie Lv, Chenghao Yang, Xitao Yang

**Affiliations:** ^1^Shanghai Ninth People’s Hospital, School of Medicine, Shanghai Jiao Tong University, Shanghai, China; ^2^Shanghai Putuo People's Hospital, Putuo, China

**Keywords:** global burden of disease, cardiovascular disease, mortality, disability-adjusted life years, age-period-cohort models, GBD

## Abstract

**Objectives:**

This study aimed to quantify the global cardiovascular disease (CVD) burden attributable to diet high in sugar-sweetened beverages (SSB) among adults aged 60 years and older using data from the Global Burden of Disease (GBD) Study 2019.

**Methods:**

We extracted data on CVD mortality, disability-adjusted life-years (DALYs), and risk-factor exposures from the GBD 2019 study for people aged 60 and older. Age-period-cohort models were used to estimate the overall annual percentage change in mortality and DALY rate (net drift, % per year), mortality and DALY rate for each age group from 1990 to 2019 (local drift, % per year), longitudinal age-specific rate corrected for period bias (age effect), and mortality and Daly rate for each age group from 1990 to 2019 (local drift, % per year). And period/cohort relative risk (period/cohort effect).

**Results:**

Between 1990 and 2019, global age-standardized CVD mortality (ASMR) and disability-adjusted life years (DALY) rates attributable to high SSB intake decreased, with larger reductions in high-SDI regions. ASMR declined from 19.5 to 13 per 100,000 (estimated annual percentage change (EAPC): −1.46%) and ASDR declined from 345.8 to 220.6 per 100,000 (EAPC: −1.66%). Age-period-cohort analysis showed CVD deaths and DALYs increased exponentially with age, peaking at 85–89 years. Period effects indicated declining CVD mortality and DALY rates since 1999, especially in higher-SDI regions. Cohort effects demonstrated consistent risk declines across successive generations born between 1900 and 1959. Predictions suggest continuing decreases through 2045 globally, but slower declines in lower-SDI regions.

**Conclusion:**

In conclusion, this comprehensive assessment of global CVD burden among older adults attributable to high SSB intake highlights major achievements but also persistent areas needing attention. Favorable declining mortality and DALY rate trends reflect substantial progress in CVD control amid population growth and aging.

## Introduction

Cardiovascular disease (CVD), mainly ischemic heart disease (IHD) and stroke, is the leading cause of death worldwide and a leading cause of disability (1, 2). CVD pathology is characterized by impaired endothelial function followed by inflammation of the vessel wall, leading to the formation of atherosclerotic lesions, leading to myocardial infarction and stroke (3). The increase in major risk factors such as obesity and diabetes has further increased the burden of vascular disease in both industrialized and developing countries (4, 5). The global prevalence of CVD nearly doubled from 1993 to 2019.4 and is projected to continue to increase through 2024 (6).

Sugar-sweetened beverages (SSB) are defined as any liquids that are sweetened with various forms of added sugars like sucrose (table sugar), high fructose corn syrup, or fruit juice concentrates. These beverages include regular sodas, fruit drinks, sports drinks, energy drinks, sweetened waters, and coffee and tea beverages with added sugars. A typical 12-fl oz. (355-mL) serving of soda contains 35.0–37.5 g of sugar and 140–150 calories (7, 8). The definition of SSB can vary across different countries. For instance, in the United States, SSB are often defined based on their content of high fructose corn syrup or sucrose. In contrast, European countries might include beverages with other forms of added sugars in their definitions. Additionally, in some developing countries, both traditional sweetened beverages and modern industrially produced SSB are considered under this category. These variations in definition are influenced by differences in national policies on food and beverage regulation and dietary culture (9). SSB have emerged as an important risk factor, with growing evidence of a consistent association between high SSB intake and many pressing public health issues, including weight gain, type 2 diabetes, dental caries, and heart disease (7, 10–13). Many health authorities have called for a reduction in SSB consumption. In addition, many public policies have been implemented to limit SSB intake in order to improve health and curb rising medical costs.

Recent studies show that CVD was the underlying cause of 9.6 million deaths in men and 8.9 million deaths in women in 2019, accounting for about one third of all deaths globally (14). Of those deaths, 6.1 million were among those aged 30–70 years. The largest number of CVD deaths was in China, followed by India, the Russian Federation, the United States of America, and Indonesia (14).

Further understanding of the CVD burden attributable to SSB is essential for disease control and appropriate healthcare planning, which helps disease control authorities to develop targeted prevention programs to reduce SSB consumption. The 2019 Global Burden of Disease (GBD) quantifies the burden of 369 diseases and injuries and 87 risks in 204 countries and territories from 1990 to 2019. Using the 2019 Global Burden of Disease (GBD) data, age-period-cohort (APC) model was used to analyze the trends of CVD attributable to high SSB consumption at global, regional and national levels from 1990 to 2019.

## Methods

### Data sources

The GBD 2019 study is a large international collaboration, supported by the Institute for Health Metrics and Evaluation and sustained by ongoing cross-country collaboration. These available epidemiological data have been used to estimate the global burden of 369 diseases. Injuries and 87 risk factors by age and gender in 204 countries and regions from 1990 to 2019 (7, 11). Repeated cross-sectional data of cardiovascular disease mortality, disability-adjusted life year (DALY) and 95% UI were collected and used by gender, age, region, and country in the world and different regions for more than 30 years. Specifically, the mortality rate refers to the number of deaths in the population during a specific period. DALY is the sum of potential years of life lost (YLL) due to premature death and productive years of life lost (YLD) due to disability. The 95% UI is a range of values that reflects the certainty with which an estimate can be made from the 25 and 975th ordered values derived from the 1,000-fold posterior distribution. We also obtained SDI information for each country or region based on lagged *per capita* distributive income, the average educational attainment of those aged 15 years and older, and the total fertility rate for women under 25 years of age. This measure ranges from 0 to 1 and represents the social and economic status of health outcomes at each study site. Higher values indicate higher socioeconomic levels. Countries were divided into five quintiles based on SDI values: low, moderate-low, medium, and high-moderate-high.

### Case definition

Each CVD cause and related health states were identified with standard case definitions. IHD represented acute myocardial infarction, chronic stable angina, chronic IHD, and heart failure due to IHD. Myocardial infarction was defined according to the Fourth Universal Definition of Myocardial Infarction and was adjusted to include out-of-hospital sudden cardiac death. Stable angina was defined according to the Rose Angina Questionnaire. Stroke was defined according to the World Health Organization definition and was estimated separately for three subcategories: (1) ischemic stroke (IS); (2) intracerebral hemorrhage; and (3) subarachnoid hemorrhage (15). Lower extremity peripheral artery disease (PAD) was defined by an ankle brachial index of <0.9 (16).

According to GBD 2019, high SSB consumption is defined as the consumption (in grams) of any beverage containing 50 calories per 226.8 g serving, including carbonated drinks, sodas, energy drinks, and fruit drinks, but excluding 100% fruit and vegetable juices (17). GBD 2019 calculates population attributable fractions using risk exposures, meta-analysis-based relative risk estimates, and theoretical minimum risk levels identified for each risk outcome pair. The theoretical minimum risk exposure level for high SSB is zero, and the risk function is monotonically increasing (17). GBD 2019 uses the Comparative Risk Assessment Framework (CRA) to assess mortality and DALY due to CVD due to high SSB intake, which includes acute myocardial infarction, chronic stable angina, chronic IHD, and IHD-related heart failure. Trends in CVD burden attributable to high SSB consumption were quantified by age-standardized mortality rates (ASMR) and age-standardized disability-adjusted life years (ASDR) per 100,000 people.

### Statistical analysis

Overall time trend analysis of CVD mortality and DALY in the aged.

The first aim of this study was to investigate the temporal trends in the mortality and DALY rate of CVD from 1990 to 2019. We used ASMR and ASDR to eliminate the effects of demographic differences. Trends in ASMR and ASDR for CVD burden due to high SSB intake were determined by estimated annual percentage change (EAPC). The EAPC is a widely used measure of age-standardized rate (ASR) trends. To do so, we calculated ASR per 100,000 using the following formula:

$$ASR=\frac{\sum_{i=1}^{A}a_{i}w_{i}}{\sum_{i=1}^{A}w_{i}}\times 10,000$$

where *a_i_*: the age-specific rate in *i*th the age group; w: the number of people in the corresponding *i*.th age group among the standard population; A: the number of age groups. To examine the temporal patterns of incidence, mortality, and DALYs, we calculated the EAPC rates. The EAPC serves as a prevalent metric in epidemiological studies to ascertain temporal evolutions in ASRs of diseases. The coefficient, denoted as β, is derived from the natural logarithm of the ASRs. Herein, *y* represents ln(ASR) while. *x* corresponds to the calendar years. The EAPC, accompanied by its 95% confidence interval (CI), was determined utilizing the ensuing linear regression model:

y=α+βx+ε

$$\mathrm{EAPC}=100*\left(\exp\;\left(\beta \right)\right)-1$$

The trend of the ASR can be discerned by analyzing the EAPC and its corresponding 95% CI. If the EAPC value and the lower limit of the 95% CI are both positive, this indicates an upward trend in the ASR. Conversely, if both the EAPC value and the upper limit of the 95% CI are negative, this suggests a downward trend in the ASR (11). To predict the future disease burden from 1990 to 2045, we utilized a log-linear age-period-cohort model. This model restricts linear trend projection and curbs exponential growth, rendering it suitable for fitting recent trends. We implemented the model in R using the NORDPRED package. To explore the factors influencing the changes of disease burden, the relationships between ASRs and SDI were calculated globally and in 20 geographic regions using Pearson’s correlation analysis from 1990 to 2019.

### Age–period–cohort analysis

The second objective was to conduct an age-period-cohort (APC) analysis to assess the different effects of age, period, and birth cohort on CVD mortality and DALY rate. The age factor reflects the social and biological dynamics of aging. Period effects refer to the effects of events and changes, such as updates in diagnostic criteria or advances in treatment, on the statistics for bipolar disorder across age groups. Cohort effect refers to the changes of disease impact caused by different exposure levels of risk factors in different population generations. APC Web Tool14 (Biostatistics Branch, National Cancer Institute, Bethesda, Md; http://analysistools.nci.nih.gov/apc/). Key parameters include: (a) net drift, representing the overall annual percentage change by calendar year and birth cohort in a log-linear fashion; (b) Local drift, with log-linear trends for each age group expressed by calendar year and birth cohort, detailing the annual percentage change for each age group; (c) longitudinal age curves showing adjusted longitudinal age-standardized rates (ASR) in the reference cohort, accounting for period bias; (d) Period relative risk (RR), time-related risk relative to the reference period, adjusted for age and nonlinear cohort effects; and (e) Cohort RR, comparing the risk of the birth cohort relative to the reference cohort, adjusting for age and nonlinear period effects. Wald chi-square tests were used to determine the significance of estimable parameters and functions. We report overall age, period, and cohort effects of CVD globally and further disaggregate these effects by SDI and sex. Analyses and graphical representations were performed with the use of R statistical software, version 4.3.0. Two-sided *p* values of less than 0.05 were considered to indicate statistical significance.

## Results

### Trends in CVD burden attributable to diet high in SSB

**Figure 1:**
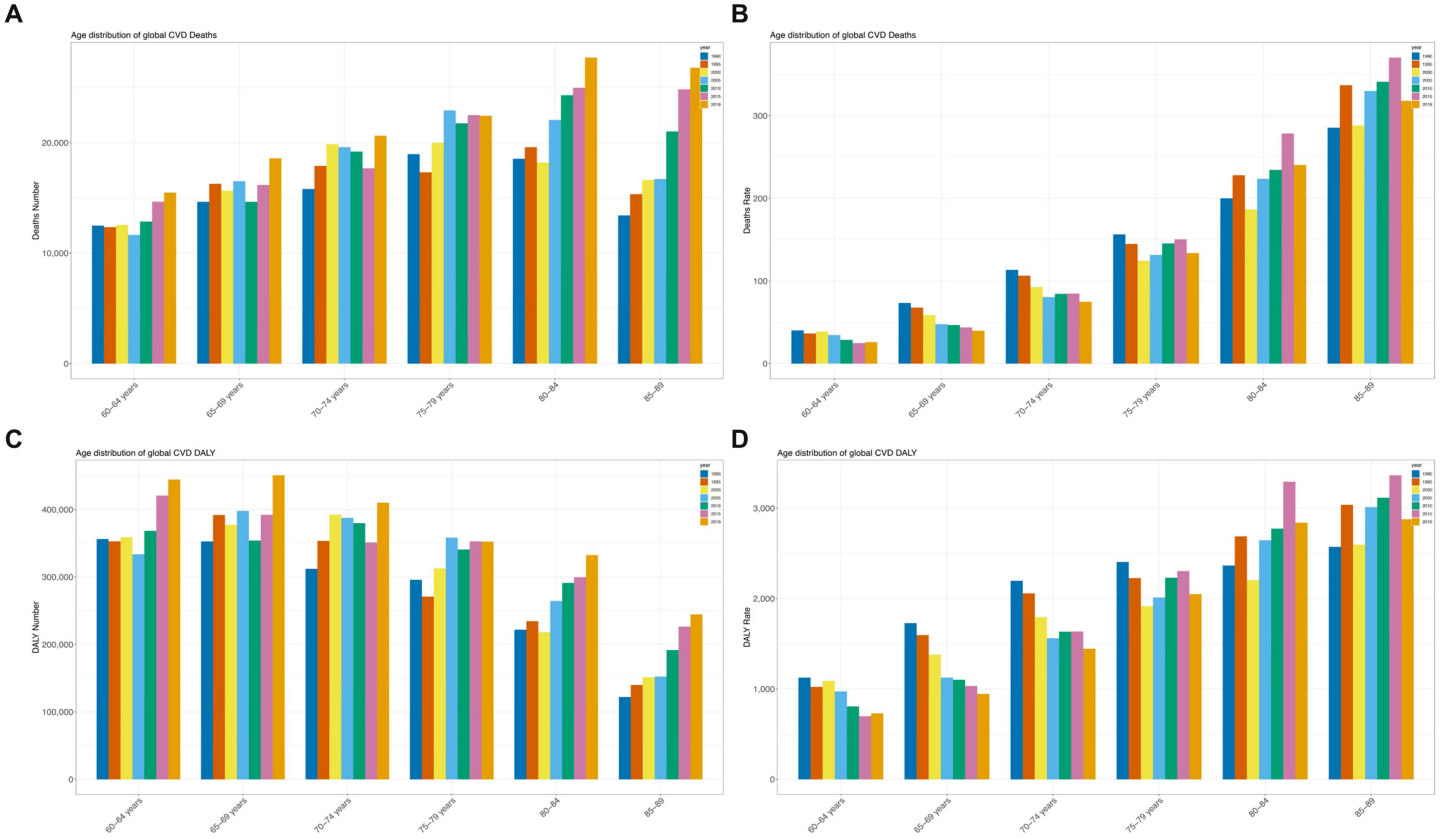
Trends in cardiovascular disease mortality and disability-adjusted life years (DALYs) from 1990 to 2019. **(A)** Trends in deaths; **(B)** Mortality trends; **(C)** Trends in DALY cases; **(D)** Trends in DALY rates.

**Figure 2:**
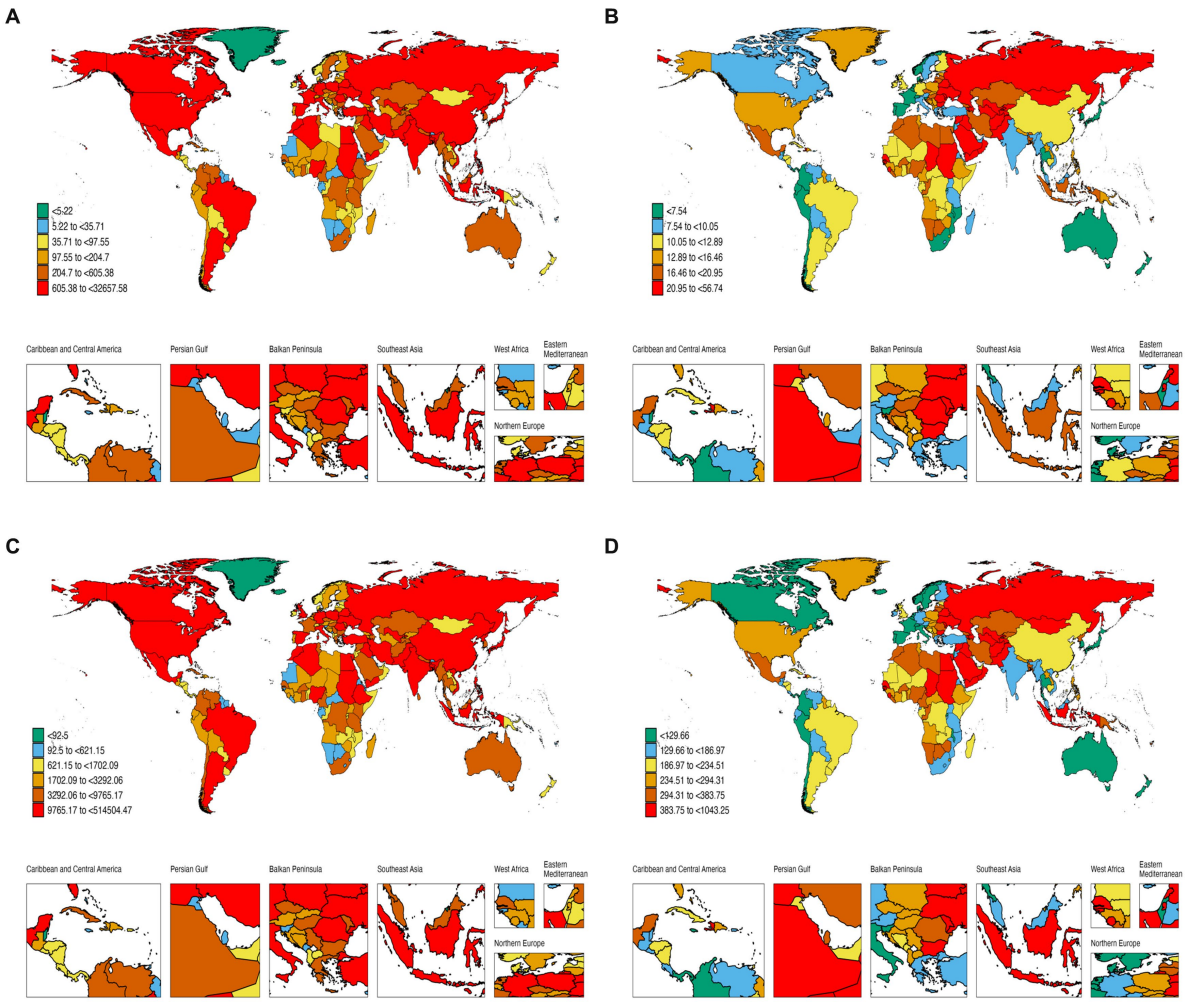
Global burden of disease of CVD attributed to high dietary SSB in 204 countries and territories. **(A)** Mortality from CVD; **(B)** ASMR of CVD; **(C)** DALY rate of CVD; **(D)** ASDR of CVD.

**Figure 3:**
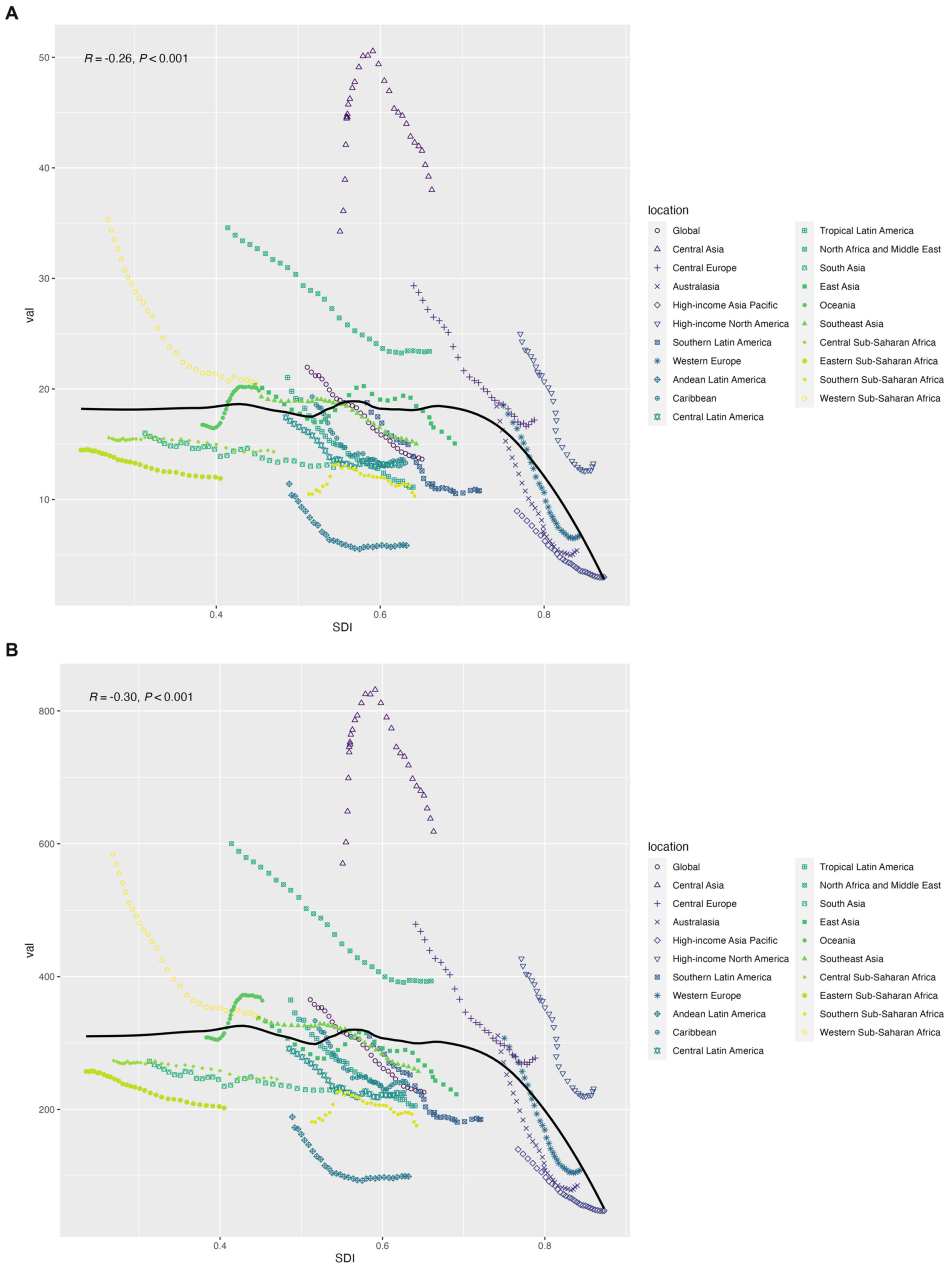
**(A)** The association between age-standardized cardiovascular disease mortality and socio-demographic indices; **(B)** Association between age-standardized DALY rate of cardiovascular disease and socio-demographic index.

Between 1990 and 2019, the global age-standardized mortality rate (ASMR) for CVD attributable to high SSB consumption decreased by 1.46 per year, from 19.5 to 13 per 100,000. High SDI (−3.22, 95%CI: −3.42 to −3.02), high and middle SDI (−1.59, 95%CI: −1.82 to −1.36), Australasia (−4.35, 95%CI: −4.59 to −4.11), Western Europe (−3.42, 95%CI: −3.66 to −3.17), High-income Asia Pacific (−2.78, 95%CI: −3.1 to −2.46), and High-income North America (−2.89, 95%CI: −3.15 to −2.62). In contrast, there was an increase in ASMR in the Oceania region (from 13.9 to 16.9 per 100,000 population) (Table 1; Figures 1A,B). At the national level, in 2019, China reported the highest CVD mortality due to high SSB consumption (32657.6, 95%UI: 17524.2–51810.4), and the lowest mortality due to Tokelau and Niue (Figure 2A). For ASMR, Tajikistan reported the highest at 56.7 per 100,000 (95%CI: 17.2–101.7); in contrast, the Republic of Korea reported the lowest ASMR (2.4 per 100,000, 95%CI: 0.9–4.6) (Supplementary Table S1; Figure 2B). In addition, Timor-Leste had the largest increase in EAPC (2.66, 95%CI: 2.53–2.79) and Denmark, Bahrain, and Bahrain had the most pronounced decreases (Supplementary Table S1; Supplementary Figure S1A). Figure 3A shows a significant negative correlation between ASMR and SDI (*p* < 0.001). In general, the ASMR of CVD caused by high SSB consumption showed a downward trend in the world and all SDI regions. The ASMR of high SDI and low-medium SDI regions was always lower than that of the world, on the contrary, the ASMR of low SDI and high-medium SDI regions was higher than that of the world (Supplementary Figure S2).

**Table 1:** Deaths of cardiovascular diseases between 1990 and 2019 in the old at the global and regional level.

Location	1990		2019		EAPC_95%CI
	Number_95%UI	ASR	Number_95%UI	ASR	
Global	93852.7 (65782.9–118639.9)	19.5 (13.7–24.7)	131624.4 (87175–171,859)	13 (8.6–17)	−1.46 (−1.53 to −1.38)
High SDI	27533.3 (13614.3–39515.3)	20.7 (10.2–29.7)	21604.5 (9115.9–33042.4)	9.2 (3.9–14.1)	−3.22 (−3.42 to −3.02)
High-middle SDI	30357.5 (19596.1–40241.8)	22.7 (14.6–30)	38732.1 (23130–53117.1)	15 (8.9–20.5)	−1.59 (−1.82 to −1.36)
Middle SDI	20727.1 (15310.9–26,515)	17.5 (12.9–22.4)	41886.9 (27269.5–57196.8)	13.9 (9–19)	−0.53 (−0.71 to −0.36)
Low-middle SDI	10220.4 (7711.4–12935.1)	15.1 (11.4–19.1)	20382.6 (14054.5–26712.1)	12.7 (8.7–16.6)	−0.47 (−0.56 to −0.37)
Low SDI	4968.7 (3744.7–6377.3)	18.8 (14.1–24.1)	8952.6 (6462.3–11712.6)	15.7 (11.3–20.5)	−0.66 (−0.72 to −0.59)
Andean Latin America	247.6 (102.4–410.6)	10.6 (4.4–17.6)	381.1 (133.2–675.4)	5.7 (2–10.1)	−1.88 (−2.35 to −1.41)
Australasia	546.7 (147.7–1050.3)	18 (4.9–34.6)	359 (97.5–675.5)	5.8 (1.6–10.9)	−4.35 (−4.59 to −4.11)
Caribbean	596.4 (301.9–927.9)	18.8 (9.5–29.2)	829.6 (383.3–1331.7)	13.3 (6.1–21.3)	−1.24 (−1.45 to −1.02)
Central Asia	1728.3 (965.5–2523.1)	31 (17.3–45.3)	2535.6 (1261.3–3927.1)	30.4 (15.1–47)	0.02 (−0.45 to 0.49)
Central Europe	5142.7 (2073.7–7884.7)	26.9 (10.8–41.2)	5042.7 (1650.3–8124.6)	17.7 (5.8–28.5)	−1.45 (−1.66 to −1.23)
Central Europe, Eastern Europe, and Central Asia	20113.3 (12045.5–27865.7)	32.9 (19.7–45.6)	21660.6 (12359.8–29830.7)	26.3 (15–36.2)	−1.03 (−1.37 to −0.7)
Central Latin America	1516.3 (808.5–2189.1)	15.9 (8.5–23)	3591.4 (1790.3–5309.4)	12.7 (6.4–18.8)	−0.74 (−0.95 to −0.54)
Central Sub-Saharan Africa	307.7 (165.3–532.5)	12.2 (6.5–21.1)	649.9 (282.4–1222.6)	11.7 (5.1–22.1)	−0.08 (−0.25 to 0.1)
East Asia	16598.9 (9816.2–24211.1)	15.9 (9.4–23.2)	33656.6 (18557.5–52999.9)	12.8 (7.1–20.1)	−0.26 (−0.59 to 0.07)
Eastern Sub-Saharan Africa	1,016 (648.1–1445.9)	12.1 (7.7–17.3)	1747.1 (1062.5–2,568)	10 (6.1–14.7)	−0.77 (−0.81 to −0.72)
High-income Asia Pacific	2,066 (1010.4–3107.6)	8.2 (4–12.4)	1997.5 (943.6–3025.7)	3.7 (1.8–5.6)	−2.78 (−3.1 to −2.46)
High-income North America	11643.1 (4911.2–18679.7)	25.8 (10.9–41.4)	10726.9 (3790.6–18468.5)	13.4 (4.7–23)	−2.89 (−3.15 to −2.62)
North Africa and Middle East	5801.6 (3742.9–7832.6)	29.6 (19.1–39.9)	10020.4 (6048.5–13824.3)	20.7 (12.5–28.5)	−1.33 (−1.4 to −1.27)
Oceania	45.4 (21.4–81.4)	13.9 (6.6–24.9)	123.5 (50.5–231)	16.9 (6.9–31.6)	0.89 (0.72–1.06)
South Asia	7880.7 (5425.2–10612.2)	12.6 (8.7–17)	19365.2 (12059–26,743)	11.6 (7.2–16)	−0.09 (−0.19 to 0.01)
Southeast Asia	4722.9 (3330.1–6204.1)	16.4 (11.6–21.5)	9264.9 (6370.7–12249.5)	13.1 (9–17.3)	−0.68 (−0.86 to −0.49)
Southern Latin America	1020.8 (273.3–1906.2)	17.5 (4.7–32.7)	1147.4 (232.6–2092.3)	11 (2.2–20.1)	−1.71 (−1.91 to −1.52)
Southern Sub-Saharan Africa	300.3 (158.8–448.2)	9.4 (5–14)	597.1 (299.3–896.2)	9.1 (4.5–13.6)	−0.14 (−0.48 to −0.2)
Tropical Latin America	1,974 (989.3–2931.8)	18.5 (9.3–27.5)	3144.8 (1666.4–4491.2)	10.7 (5.7–15.2)	−1.79 (−1.86 to −1.73)
Western Europe	14,490 (7064.6–20955.7)	19.4 (9.5–28.1)	8883.3 (3647.1–13423.7)	8.1 (3.3–12.3)	−3.42 (−3.66 to −3.17)
Western Sub-Saharan Africa	2,965 (2033.6–4,136)	29.6 (20.3–41.3)	3,478 (2273.1–4897.3)	17.3 (11.3–24.3)	−1.95 (−2.08 to −1.82)

EAPC, Estimated annual percentage change; SDI, Sociodemographic index; UI, Uncertainty interval.

For DALY, the global age-standardized rate decreased from 345.8 per 100,000 in 1990 to 220.6 per 100,000 in 2019, with an EAPC of −1.66 (95% CI: −1.74 to 1.58) (Figure 1B; Supplementary Table S2). The EAPC was −3.29 (95%CI: −3.52 to −3.05) for high-SDI countries, −1.96 (95%CI: −2.24 to −1.69) for high-middle SDI regions, and − 0.91 (95%CI: −2.24 to −1.69) for middle SDI regions (−1.06 to 0.77), low—SDI in the area of 0.62 (95% CI: 0.71–0.53), low SDI area is 0.82 (95% CI: 0.88–0.76) (Supplementary Table S2). Timor-Leste (2.4, 95%CI: 2.31–2.48), Uzbekistan (2.07, 95%CI: 1.16–2.99), and Lesotho (1.89, 95%CI: 1.63–2.15) had the highest rates of increase. In contrast, the largest reductions in DALY rate attributed to high dietary SSAs were seen in Denmark (−5.69, 95%CI: −6.07 to 5.31) and Bahrain (−5.09, 95%CI: −6.07 to 5.31) (5.5–4.68), and Norway (5.02, 95% CI: 5.2–4.84) (Supplementary Table S3; Figures 2C,D; Supplementary Figure S1B). Figure 3B shows a significant negative correlation between ASDR and SDI (*p* < 0.001). Overall, the ASDR for CVD due to high SSB consumption showed a downward trend globally and across SDI regions, similar to the ASMR (Supplementary Figure S2).

Interestingly, the results of the gender analysis suggested that the male to female ratio of CVD due to high SSB consumption decreased with age among people over 60 years old, but the ASMR and ASDR of men were higher than those of women globally and in all SDI regions (Supplementary Figure S3).

### Age, period, and cohort effects on the global trend

**Figure 4:**
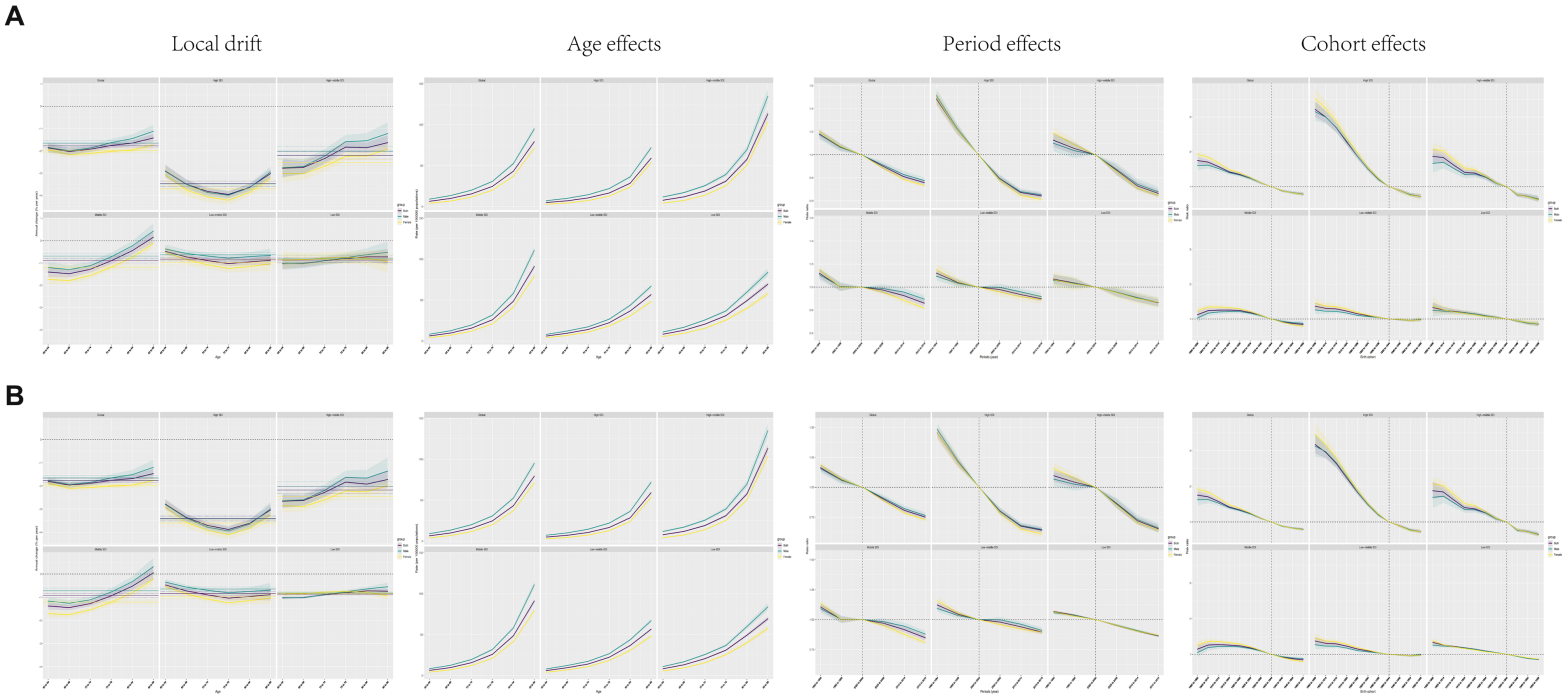
Local drift, age effect, period effect, and cohort effect of CVD in the world and in each SDI region from 1990 to 2019. **(A)** Mortality; **(B)** DALY.

From 1990 to 2019, the CVD mortality rate attributable to high dietary SSB increased with age among people older than 60 years of age (Figure 4; Supplementary Figure S4), with a steeper increase among women (net drift of −1.74 among people aged 85–89 years; 95% CI − 1.86 to −1.61) (Supplementary Table S4). Similar trends were observed among men (net drift of −1.12 for those aged 85–89; 95% CI, − 1.4, − 0.84) (Supplementary Table S4). Rates of death from CVD and DALY were highest at ages 85–89 years for both women and men (Figure 4; Supplementary Tables S4–S7).

The period effect showed that the mortality and DALY rates declined from 1999 to 2019 regardless of sex (Figure 4). Compared with 2000–2004, the mortality rate was the lowest in 2015–2019 (0.76, 95%CI: 0.74, 0.78) and the highest in 1990–1994 (1.18, 95%CI: 1.15, 1.21, Supplementary Table S8). A similar trend was observed for DALY rates (Supplementary Table S9; Figure 4).

There was an overall downward trend in cohort mortality and DALY rates for successive 10-year birth cohorts from 1900–1909 to 1950–1959, with declines since the 1940–1949 cohort (Figure 4; Supplementary Tables S10, S11). Mortality and DALY risk were higher in the earlier birth cohort (before 1940) than in the centralized birth cohort (1940–1959). In the cohort before the reference group, 1935 to 1944, men had lower risks for both measures than did women. However, in more recent cohorts, the risk among men equals or exceeds that among women (Figure 4; Supplementary Tables S10, S11).

### Age, period, and cohort effects by SDI quintiles

From 1990 to 2019, the CVD mortality and DALY rates attributable to high dietary SSB increased with age in all SDI regions (Figure 4), peaking in the 84–89 age group. Interestingly, the magnitude of change was greater among men than among women across regions, and the magnitude of change was similar between men and women (Figure 4; Supplementary Tables S12–S14). In addition, the increasing trends of mortality and DALY rates with age were most pronounced in high-middle SDI regions (Figure 4).

From 1990 to 2019, the mortality rate and DALY rate of CVD attributable to high dietary SSB declined in all SDI areas, especially in high SDI areas, with no significant difference between men and women. In contrast, low-medium SDI regions showed the smallest changes (Figure 4; Supplementary Tables S15, S16).

For birth cohorts after the reference group during 1935 to 1944, changes in mortality and DALY rates were modest in the moderate-to-low SDI regions, in contrast to substantial decreases in the remaining SDI regions (Figure 4; Supplementary Tables S18, S19).

### Future burden of bipolar disorder

**Figure 5:**
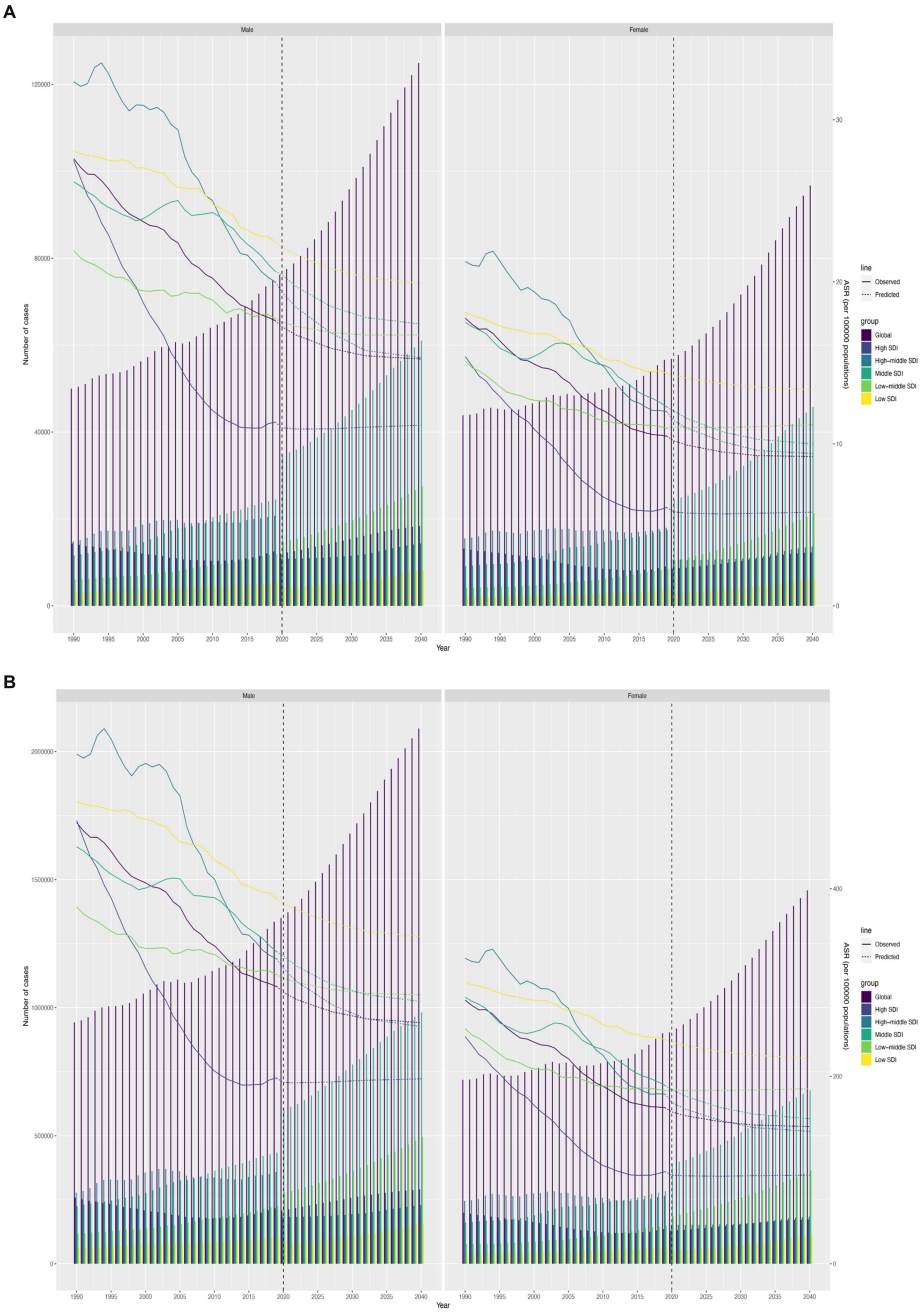
Future forecasts of GBD in cardiovascular diseases. **(A)** Trend of mortality; **(B)** Trend of DALY rate.

Figure 5 plots the predicted trajectories of CVD attributed to high SSB in the population over 60 years of age, indicating a decreasing rate of increase in the global burden of these diseases. Notably, the mortality and DALY rates in low, low-medium SDI regions were higher than those in other regions and the global average. Regions with high-middle and high SDI reported lower mortality rates than the global average, but prevalence changes were more stable. In addition, gender differences were evident, with the trends of mortality and DALY rates in men being significantly greater than those in women (Figure 5).

## Discussion

This study provides a comprehensive assessment of the global burden and trends of cardiovascular disease (CVD) mortality and disability-adjusted life years (DALYs) attributable to high sugar-sweetened beverage (SSB) consumption among adults aged 60 years and older from 1990 to 2019 using data from the Global Burden of Disease (GBD) Study 2019. Several key findings emerged.

First, we found that the age-standardized CVD mortality rate (ASMR) and DALY rate (ASDR) attributable to high SSB intake decreased globally and across all sociodemographic index (SDI) regions from 1990 to 2019, with an estimated annual percentage change (EAPC) of −1.46% for ASMR and − 1.66% for ASDR. The largest reductions were observed in high-SDI regions like Western Europe, High-Income North America, and Australasia. In contrast, ASMR and ASDR increased in lower-SDI regions like Sub-Saharan Africa and Oceania over the study period. Significant negative correlations were observed between SDI level and ASMR as well as ASDR, highlighting the influence of socioeconomic development on CVD burden due to SSB. These findings are consistent with previous studies showing favorable CVD mortality trends in higher-income regions but worsening trends in lower-income regions globally (14, 18, 19). The reasons for divergent CVD mortality trends across SDI levels likely relate to differences in health system capacity, access to preventive services, diagnosis and treatment rates, and underlying risk factor profiles. As countries develop economically, investments in health systems and policies to tackle key CVD risks like unhealthy diets may accelerate declines in CVD deaths (20, 21). However, lower-SDI countries continue to face resource constraints and have competing health priorities, limiting CVD control efforts. Among the different types of CVDs, ischemic heart disease (IHD) showed the most significant decline in mortality rates (9). The age-standardized mortality rate (ASMR) for IHD decreased markedly, particularly in high-SDI regions, which can be attributed to better management and prevention strategies, including improved medical treatments, lifestyle changes, and public health interventions targeting risk factors like high SSB intake (9, 20).

Second, our age-period-cohort (APC) analysis provides novel insights into the drivers of changing CVD mortality and DALY rates attributed to high SSB intake over the past 3 decades. We found that CVD burden increased exponentially with age, peaking at 85–89 years for both sexes globally—aligning with the natural history of CVD where manifestations of atherosclerotic vascular disease accumulate over decades of exposure until clinically apparent events emerge later in life (22). However, more pronounced increases in CVD deaths and DALYs with age emerged in higher-SDI regions compared to lower-SDI regions. This likely reflects better control of competing mortality risks (e.g., infectious diseases) in early life in wealthier nations, enabling more individuals to survive into old age where CVD emerges as a dominant source of morbidity and mortality (23).

Regarding period effects, CVD mortality and DALY rates declined steadily from 1999 onwards globally—coinciding with concerted global efforts to tackle key CVD risks like tobacco use, hypertension, diabetes, and hyperlipidemia over the past 2 decades (20, 24). We also noted favorable period effects on CVD burden across all SDI regions, but again more markedly in higher-SDI areas. This aligns with the wider implementation of evidence-based policies and programs to curb smoking, improve diets, enhance physical activity levels, and expand screening and treatment in these resource-rich settings (20). However, period declines were less pronounced in lower-SDI regions, highlighting major CVD prevention and control gaps.

For cohort effects, our study shows consistent declines in CVD mortality and DALY rates for successive generations born between 1900–1909 and 1950–1959 globally. These falling cohort trends likely reflect declining lifetime exposures to key CVD risks like smoking, elevated blood pressure, and cholesterol across cohorts due to societal changes (25). Some theories posit that improved living standards, education, nutrition, sanitation, and healthcare in more contemporary cohorts may confer CVD resilience even in the face of other risks like obesity and diabetes at the population level (26). However, the slower cohort declines observed in lower-SDI regions versus higher-SDI regions again underscores widening socioeconomic inequalities in lifetime CVD risk management globally.

Our future predictions based on recent trends forecast continuing falls in global CVD mortality and DALY rates attributable to high SSB intake through 2045. However, more modest declines are expected for lower-SDI regions. Given projections that nearly 80% of global CVD burden will occur in low- and middle-income countries by 2030 (27), concerted policy action is needed to avert this growing inequality. Investments to curb SSB intake should be a priority, given consistent links between high consumption and adverse cardiometabolic outcomes (9, 28–30). Some cost-effective, population-level interventions could include raising SSB prices through taxation, enforcing advertising restrictions, introducing warning labels, and reformulating products (31–33). However, lower-resourced nations may face industry interference, financial constraints, poor governance, and weak health systems that could hamper intervention success (34). Therefore, global coordination and funding support from international agencies to assist suitable, context-specific SSB control policies in disadvantaged countries will be vital (35).

In recent years, various policies and interventions have been implemented globally to reduce sugar intake and combat CVD (36). These efforts focus on taxation, regulation, and public health education (37). One prominent approach is the implementation of SSB taxes. For instance, Mexico introduced a nationwide SSB tax in 2014, leading to a significant reduction in SSB purchases and a potential decrease in obesity and related diseases, including CVD (38). Similarly, cities in the United States, such as Berkeley, Philadelphia, and Seattle, have adopted SSB taxes, showing promising results in reducing consumption and improving public health outcomes (33, 39). In addition to taxation, regulatory measures have been adopted. Chile’s comprehensive food labeling and marketing regulation, implemented in 2016, requires warning labels on products high in sugar, sodium, and saturated fats, and restricts their marketing to children (40). This policy has led to significant changes in consumer behavior and product reformulation by manufacturers. Public health campaigns and educational initiatives are also crucial. The United Kingdom’s “Change4Life” campaign, for example, provides resources and tools to help families make healthier food and drink choices, contributing to the reduction of CVD risk factors (41). These examples demonstrate that various countries have successfully implemented policies to reduce sugar intake, which is essential for improving cardiovascular health and reducing the global burden of CVD. Supplementary Figure S5 shows various approaches to reducing mortality from cardiovascular disease caused by SSB.

Our study has several strengths worth highlighting. First, we utilized extensive, high-quality data on CVD morbidity, mortality, exposures, and social indicators across 204 countries and territories over 30 years from the GBD Study to deliver robust, reliable estimates of attributable CVD burden. Second, our advanced statistical approach enabled in-depth examination of temporal dynamics and drivers of changing disease rates. The APC framework isolated distinct age, period, and cohort influences amidst background epidemiological transitions. Third, we quantified CVD burden due to high SSB consumption specifically among older adults—an under-studied, high-risk group. As populations age rapidly, evidence to guide prevention in this demographic is vital. Fourth, our high-resolution results by country/region, SDI level, and sex provided tailored insights to inform policy prioritization and resource allocation for maximum CVD control.

### Limitations

Despite its strengths, our study has some limitations worth acknowledging. First, the availability and quality of data sources varied across locations and over time within the GBD modeling framework. Potential biases from incomplete vital registration systems, miscoded causes of death, and missing data may affect estimates despite extensive corrections. Second, high between-country heterogeneity exists regarding definitions, diagnostics, healthcare access, coding practices, and cultural factors that may distort disease estimates. However, the GBD Study implements extensive data corrections to enhance cross-national comparability. Third, determining causality between SSB intake and CVD outcomes remains challenging given multiple interconnected diet and lifestyle risks. Further research should elucidate independent risk relationships. Fourth, potential residual confounding from unmeasured factors might affect estimated disease associations. Nonetheless, GBD risk-outcome pairs draw from the best available evidence synthesized systematically across cohort studies. Fifth, summary exposure measures like the SDI may incompletely capture societal complexities within countries. Further subnational data could provide additional context. Finally, accuracy of future predictions depends considerably on underlying model assumptions and sustained trends, although triangulated projections based on complementary statistical approaches enhanced robustness.

## Conclusion

In conclusion, this comprehensive assessment of global CVD burden among older adults attributable to high SSB intake highlights major achievements but also persistent areas needing attention. Favorable declining mortality and DALY rate trends reflect substantial progress in CVD control amid population growth and aging. However, heavier and potentially increasing burdens projected for disadvantaged regions underscore widening inequities that demand urgent policy action aligned with the sustainable development goal agenda of leaving no one behind. Furthermore, with age, period, and cohort risk declines less marked in lower-SDI countries, a proactive lifespan approach that targets high-risk groups via cost-effective primary and secondary prevention from early life is imperative. Globally coordinated, multi-sectoral efforts incorporating regulation, pricing, reformulation, and targeted education around SSB should be part of integrated strategies to promote cardiovascular health across the life course, especially in underserved populations.

## Data availability statement

The original contributions presented in the study are included in the article/Supplementary material; further inquiries can be directed to the corresponding author.

## Ethics statement

Ethical review and approval was not required for the study on human participants in accordance with the local legislation and institutional requirements. Written informed consent from the patients/participants or the patients’/participants’ legal guardian/next of kin was not required to participate in this study in accordance with the national legislation and the institutional requirements.

## Author contributions

JL: Formal Analysis, Methodology, Resources, Software, Writing – original draft. CY: Data curation, Formal Analysis, Validation, Writing – review & editing. XY: Conceptualization, Project administration, Resources, Writing – review & editing.
