# Supplementary material for: The global burden of cardiovascular disease attributable to diet high in sugar-sweetened beverages among people aged 60 years and older: an analysis for the global burden of disease study 2019
Source: Front Public Health. 2024 Jul 19;12:1366286. doi: 10.3389/fpubh.2024.1366286 (PMC11295280; doi:10.3389/fpubh.2024.1366286)
Supplement: Supplementary file 1 [file Table_1.DOCX]

**Supplementary material**

**Table and figures’ legends**

**Table S1.** Deaths of Cardiovascular diseases between 1990 and 2019 in the old at the 204 Countries Level.

**Table S2.** DALY of Cardiovascular diseases between 1990 and 2019 in the old at the global and regional level.

**Table S3.** DALY of Cardiovascular diseases between 1990 and 2019 in the old at the 204 Countries Level.

**Table S4.** Annual percentage change for cardiovascular diseases deaths overall (net drift) and in each age group (Local drift) by sex from 1990 to 2019 in the old.

**Table S5.** Fitted longitudinal age effects of cardiovascular diseases deaths (per 100 000 person-years) and the corresponding 95% CIs in the old.

**Table S6.** Annual percentage change for cardiovascular diseases DALY rate overall (net drift) and in each age group (Local drift) by sex from 1990 to 2019 in the old.

**Table S7.** Fitted longitudinal age effects of cardiovascular diseases DALY rate (per 100 000 person-years) and the corresponding 95% CIs in the old.

**Table S8.** Relative risk for cardiovascular diseases deaths rate of each period compared with the reference (2000–2004) and the corresponding 95% CIs by sex in the old.

**Table S9.** Relative risk for cardiovascular diseases DALY rate of each period compared with the reference (2000–2004) and the corresponding 95% CIs by sex in the old.

**Table S10.** Relative risk for cardiovascular diseases deaths rate of each birth cohort compared with the reference (cohort 1935-1944) and the corresponding 95% CIs by sex in the old.

**Table S11.** Relative risk for cardiovascular diseases DALY rate of each birth cohort compared with the reference (cohort 1935-1944) and the corresponding 95% CIs by sex in the old.

**Table S12.** Annual percentage change for cardiovascular diseases deaths rate overall (net drift) and in each age group (local drift) by sex and SDI region from 1990 to 2019 in the old.

**Table S13.** Fitted longitudinal age effects of cardiovascular diseases deaths (per 100 000 person-years) and the corresponding 95% CIs by sex and SDI region in the old.

**Table S14.** Annual percentage change for cardiovascular diseases DALY rate overall (net drift) and in each age group (local drift) by sex and SDI region from 1990 to 2019 in the old.

**Table S15.** Fitted longitudinal age effects of cardiovascular diseases DALY rate (per 100 000 person-years) and the corresponding 95% CIs by sex and SDI region in the old.

**Table S16.** Relative risk for cardiovascular diseases deaths rate of each period compared with the reference (2000–2004) and the corresponding 95% CIs by sex and SDI region in the old.

**Table S17.** Relative risk for cardiovascular diseases DALY rate of each period compared with the reference (2000–2004) and the corresponding 95% CIs by sex and SDI region in the old.

**Table S18.** Relative risk for cardiovascular diseases deaths rate of each birth cohort compared with the reference (cohort 1935-1944) and the corresponding 95% CIs by sex and SDI region in the old.

**Table S19.** Relative risk for cardiovascular diseases DALY rate of each birth cohort compared with the reference (cohort 1935-1944) and the corresponding 95% CIs by sex and SDI region in the old.

**Figure S1.** Global Burden of disease for CVD in women and men for 204 countries and territories (A) EAPC for BD mortality; (B) EAPC of BD DALY.

**Figure S2.** Trends of ASMR and ASDR of CVD in regions with different development levels from 1990 to 2019.

**Figure S3.** Mortality and disability-adjusted life years (DALY) ratio of men to women for bipolar disorder by age subgroup (A) mortality ratio of men to women; (B) The proportion of DALYs among men and women.

**Figure S4.** Temporal changes in the age distribution of CVD mortality and DALY among people aged 60 and above from 1990 to 2019.

**Figure S5.** The various ways of reducing CVD mortality due to SSBs.

**Table S1.** Deaths of Cardiovascular diseases between 1990 and 2019 in the old at the 204 Countries Level.

| Location | 1990 | | 2019 | | EAPC_95%CI |
| --- | --- | --- | --- | --- | --- |
|  | Number_95%UI | ASR | Number_95%UI | ASR |  |
| Afghanistan | 413.8 (112.4-807.6) | 48 (13.1-93.7) | 527.4 (148.4-982) | 48.9 (13.8-91.1) | -0.52 (-0.93--0.1) |
| Albania | 26.2 (8.2-56) | 11 (3.4-23.5) | 84.8 (17.9-194.7) | 15 (3.2-34.5) | 1.89 (1.4-2.38) |
| Algeria | 555.1 (159.4-1088.7) | 37.8 (10.9-74.2) | 738.8 (219.9-1571.8) | 18.8 (5.6-40) | -2.81 (-3.06--2.57) |
| American Samoa | 0.3 (0.1-0.6) | 10.7 (3-23.3) | 0.6 (0.2-1.4) | 11.5 (3.3-25.1) | 0.37 (0.25-0.5) |
| Andorra | 0.7 (0.1-1.6) | 9.2 (2.1-21.9) | 1.1 (0.2-2.4) | 6.4 (1.4-14.4) | -1.07 (-1.17--0.97) |
| Angola | 91.8 (31.9-160.9) | 22.7 (7.9-39.8) | 157.1 (45.2-314.1) | 13.7 (3.9-27.4) | -2.19 (-2.34--2.03) |
| Antigua and Barbuda | 1.7 (0.6-3) | 25.5 (8.4-44.6) | 1.8 (0.6-3.1) | 14.5 (5-25.4) | -2.26 (-2.46--2.05) |
| Argentina | 785.2 (182.8-1570.2) | 19.1 (4.4-38.2) | 862.8 (151.8-1688.7) | 12.8 (2.2-25) | -1.51 (-1.7--1.31) |
| Armenia | 159.3 (55.8-264.9) | 48.7 (17.1-81) | 153.4 (50.3-291.3) | 28 (9.2-53.1) | -2.25 (-2.42--2.08) |
| Australia | 465.7 (115.6-933.2) | 18.4 (4.6-36.9) | 295.4 (73.4-583.7) | 5.7 (1.4-11.2) | -4.5 (-4.74--4.26) |
| Austria | 239.3 (81.3-442.3) | 15.6 (5.3-28.9) | 198.2 (52.6-393.1) | 9.3 (2.5-18.5) | -2.06 (-2.24--1.89) |
| Azerbaijan | 221.2 (63.2-447.4) | 39.4 (11.3-79.7) | 363 (109.1-753.8) | 34.1 (10.3-70.9) | -0.56 (-0.79--0.33) |
| Bahamas | 3.4 (1.1-6) | 19.7 (6.5-35) | 5.9 (1.9-10.6) | 12.9 (4.2-23.4) | -1.66 (-1.88--1.43) |
| Bahrain | 6.6 (2-13.2) | 36.5 (10.9-73.4) | 10.5 (2.9-21.5) | 10 (2.7-20.6) | -4.95 (-5.4--4.49) |
| Bangladesh | 1309.4 (555.9-2149.5) | 25.1 (10.7-41.2) | 2938.4 (839.5-5409.1) | 18.8 (5.4-34.6) | -0.55 (-0.81--0.3) |
| Barbados | 4.8 (1.5-10) | 12.9 (3.9-26.5) | 4.3 (1.3-8.7) | 6.7 (2-13.6) | -2.82 (-3.09--2.54) |
| Belarus | 620.1 (180.4-1288) | 36.9 (10.7-76.7) | 959.6 (279-1916.5) | 46.2 (13.4-92.2) | 1.29 (0.74-1.83) |
| Belgium | 294.3 (83.5-584.8) | 14.7 (4.2-29.2) | 205.8 (46.4-410.9) | 7.5 (1.7-14.9) | -2.48 (-2.64--2.32) |
| Belize | 1.5 (0.5-3.1) | 13.3 (4.1-28.4) | 2.2 (0.7-4.7) | 7.3 (2.3-15.2) | -2.54 (-2.98--2.11) |
| Benin | 72 (32.2-118) | 31.1 (13.9-50.9) | 81.3 (22.9-160.3) | 15.6 (4.4-30.7) | -2.53 (-2.81--2.24) |
| Bermuda | 2.7 (0.9-4.9) | 34.7 (11.1-63.4) | 2.5 (0.9-4.3) | 15.2 (5.5-26.2) | -2.94 (-3.17--2.71) |
| Bhutan | 5.3 (1.7-9.6) | 19.8 (6.2-36.2) | 12.4 (3.3-24.2) | 18.8 (5-36.7) | -0.03 (-0.14-0.07) |
| Bolivia (Plurinational State of) | 42.4 (12.4-93.1) | 11.5 (3.4-25.2) | 96.8 (27-214.2) | 9.1 (2.5-20.2) | -0.74 (-0.98--0.5) |
| Bosnia and Herzegovina | 62.6 (21.3-139.3) | 13.1 (4.5-29.1) | 112.5 (29.6-257.6) | 14.1 (3.7-32.3) | 0.35 (0.21-0.49) |
| Botswana | 11.5 (3.5-21.8) | 17.3 (5.3-32.6) | 27.4 (8.5-52.6) | 18.2 (5.6-35) | -0.04 (-0.43-0.35) |
| Brazil | 1944.6 (965.3-2902.6) | 18.7 (9.3-27.9) | 3080.7 (1622.5-4421.7) | 10.7 (5.6-15.3) | -1.82 (-1.89--1.76) |
| Brunei Darussalam | 2.8 (1-5) | 28.3 (9.6-49.9) | 4.5 (1.4-8.1) | 13.8 (4.4-24.6) | -2.32 (-2.52--2.11) |
| Bulgaria | 555.3 (131.9-1149.9) | 33.1 (7.9-68.5) | 476.4 (116.9-974.2) | 24.7 (6-50.4) | -1.51 (-1.84--1.18) |
| Burkina Faso | 130 (59-213.2) | 25.3 (11.5-41.5) | 183.6 (50.9-353.2) | 18.6 (5.2-35.7) | -1.09 (-1.23--0.95) |
| Burundi | 37.6 (10.1-84.6) | 14.1 (3.8-31.7) | 46.8 (12.6-111.8) | 9.6 (2.6-22.8) | -1.47 (-1.52--1.42) |
| Cabo Verde | 11.5 (5.6-17.7) | 39.6 (19.3-61.1) | 15.8 (6-26.7) | 32.6 (12.5-55.3) | -0.96 (-1.3--0.61) |
| Cambodia | 74.6 (19.3-150.4) | 14.9 (3.9-30.1) | 147.3 (41-320.8) | 10.8 (3-23.4) | -1.23 (-1.34--1.13) |
| Cameroon | 108 (43.6-181.7) | 21.7 (8.7-36.4) | 207.8 (60-407.2) | 16.3 (4.7-31.9) | -1.04 (-1.21--0.87) |
| Canada | 773 (236.8-1543.1) | 18.6 (5.7-37.2) | 672.1 (170.3-1414.4) | 7.7 (1.9-16.2) | -3.6 (-3.82--3.37) |
| Central African Republic | 20.4 (5.4-43.4) | 16.4 (4.4-34.8) | 27 (7.3-63.7) | 12.7 (3.4-29.8) | -1 (-1.18--0.82) |
| Chad | 116.1 (60.4-183.7) | 35.1 (18.2-55.5) | 150.9 (53.6-261.4) | 24.9 (8.8-43.2) | -1.22 (-1.32--1.12) |
| Chile | 147.9 (45.2-284.9) | 12.3 (3.7-23.6) | 204.1 (46.3-406.8) | 6.8 (1.5-13.6) | -2.09 (-2.37--1.82) |
| China | 16133.4 (9335-23693.4) | 16 (9.3-23.5) | 32657.6 (17524.2-51810.4) | 12.8 (6.9-20.4) | -0.27 (-0.6-0.07) |
| Colombia | 245.7 (89.6-457.7) | 12.3 (4.5-23) | 428.9 (129.1-828.9) | 6.7 (2-13) | -1.9 (-2.3--1.49) |
| Comoros | 2.3 (0.7-5.4) | 8.8 (2.6-20.9) | 4.3 (1.3-10.1) | 7.8 (2.4-18.3) | -0.43 (-0.53--0.32) |
| Congo | 27.1 (7.8-52.7) | 22.2 (6.4-43.2) | 44.9 (13-92.7) | 16.7 (4.8-34.6) | -1.09 (-1.25--0.93) |
| Cook Islands | 0.1 (0-0.3) | 8.7 (2.7-19.8) | 0.2 (0.1-0.6) | 8 (1.9-19.5) | -0.31 (-0.53--0.1) |
| Costa Rica | 25.3 (7.2-52.2) | 12.4 (3.5-25.5) | 48.2 (12.5-103.5) | 7.7 (2-16.6) | -1.91 (-2.14--1.69) |
| Coted'Ivoire | 86.2 (25.9-158.2) | 20.8 (6.2-38.2) | 142.7 (39.5-300.4) | 13 (3.6-27.4) | -1.78 (-1.92--1.64) |
| Croatia | 181.5 (52.1-385.4) | 22.1 (6.3-46.8) | 191.6 (41.8-446.4) | 16.7 (3.7-39) | -0.14 (-0.5-0.22) |
| Cuba | 235.9 (71.4-497.5) | 18.6 (5.6-39.2) | 275.7 (65.4-626.3) | 12.2 (2.9-27.7) | -1.58 (-1.95--1.22) |
| Cyprus | 18.5 (5.2-40) | 17.3 (4.8-37.4) | 20.8 (6.2-42.6) | 8.3 (2.5-16.9) | -3.43 (-3.68--3.18) |
| Czechia | 607.1 (160.5-1321.4) | 33.4 (8.8-72.8) | 457.7 (112-1036.5) | 16.8 (4.1-38.1) | -2.53 (-2.64--2.42) |
| Democratic People's Republic of Korea | 330.1 (121.3-580.1) | 18.7 (6.9-32.8) | 848.6 (261.2-1530.9) | 22.2 (6.8-40) | 0.91 (0.68-1.14) |
| Democratic Republic of the Congo | 152.7 (47.5-354.4) | 8.5 (2.7-19.8) | 401.6 (108.5-937.8) | 10.7 (2.9-25.1) | 0.96 (0.59-1.33) |
| Denmark | 225.2 (68.1-454.6) | 22 (6.6-44.3) | 82.8 (22.7-168.2) | 5.8 (1.6-11.7) | -5.67 (-6.05--5.28) |
| Djibouti | 2.3 (0.8-4.2) | 17.1 (5.9-30.5) | 7.1 (1.9-15.7) | 11.6 (3.1-25.5) | -1.55 (-1.72--1.39) |
| Dominica | 1.2 (0.4-2.6) | 13.1 (4.1-27.4) | 0.9 (0.3-2) | 8.2 (2.4-17.8) | -1.87 (-2.05--1.69) |
| Dominican Republic | 51.8 (15.4-109.2) | 12 (3.6-25.4) | 177.8 (52.6-372.6) | 16.3 (4.8-34.1) | 1.68 (1.5-1.87) |
| Ecuador | 56.7 (15.8-122.3) | 9.2 (2.6-19.9) | 136.6 (34.7-300.6) | 7.5 (1.9-16.5) | -0.4 (-0.79--0.01) |
| Egypt | 1088.2 (392.9-2166.3) | 33.2 (12-66.1) | 1473.8 (567.1-2651.1) | 20.3 (7.8-36.6) | -1.37 (-1.81--0.93) |
| El Salvador | 36.1 (11.3-75.6) | 10.5 (3.3-22) | 71 (20.7-149) | 10 (2.9-21) | -0.09 (-0.23-0.06) |
| Equatorial Guinea | 3.6 (1-7.7) | 16.3 (4.3-35) | 5.2 (1.5-11) | 10.3 (3-21.6) | -1.99 (-2.31--1.67) |
| Eritrea | 7.7 (2-18.2) | 8.2 (2.1-19.4) | 21.4 (6.5-52.8) | 8 (2.4-19.7) | 0.02 (-0.05-0.1) |
| Estonia | 71.6 (25.2-149.3) | 27.1 (9.6-56.6) | 45.7 (13.9-95.4) | 13.7 (4.1-28.5) | -2.81 (-3.03--2.59) |
| Eswatini | 3.5 (1-7.7) | 11.1 (3.1-24.6) | 8 (2.2-17.2) | 12.2 (3.4-26.2) | 0.49 (0.24-0.74) |
| Ethiopia | 429.8 (171.1-768.6) | 19.6 (7.8-35.1) | 600.5 (229.8-1118.2) | 13.3 (5.1-24.8) | -1.58 (-1.7--1.47) |
| Fiji | 7.4 (2.3-17.3) | 20.2 (6.2-47.1) | 14.3 (4.3-32) | 16.6 (4.9-37) | -0.69 (-0.84--0.54) |
| Finland | 190 (63.3-350.4) | 20.7 (6.9-38.2) | 157.7 (46.5-294.8) | 10.3 (3-19.2) | -2.51 (-2.69--2.34) |
| France | 883.2 (285.6-1751.4) | 8.3 (2.7-16.5) | 630 (188.7-1228.8) | 3.8 (1.1-7.5) | -2.77 (-2.92--2.62) |
| Gabon | 12.2 (3.7-23.8) | 18 (5.5-35.3) | 14.1 (4-30.1) | 12.2 (3.5-26.1) | -1.07 (-1.19--0.95) |
| Gambia | 9.3 (2.9-16.5) | 23.9 (7.5-42.6) | 22.3 (6.2-44.5) | 21 (5.8-41.9) | -0.51 (-0.74--0.28) |
| Georgia | 312 (90.6-653.8) | 39.2 (11.4-82.2) | 158.1 (47.7-335.8) | 20.5 (6.2-43.5) | -2.56 (-2.78--2.33) |
| Germany | 3648.9 (1021.6-7070.7) | 22.8 (6.4-44.3) | 2449.5 (594.9-4801.4) | 10.6 (2.6-20.9) | -2.8 (-3.22--2.37) |
| Ghana | 177.3 (76.4-291.8) | 25.7 (11.1-42.4) | 216 (61.1-469.9) | 12.1 (3.4-26.4) | -2.62 (-2.97--2.28) |
| Greece | 250.3 (75.4-489.9) | 12.7 (3.8-24.8) | 260.7 (83.4-500.5) | 9.2 (3-17.8) | -1.16 (-1.36--0.96) |
| Greenland | 0.8 (0.2-1.7) | 21.6 (5.4-46.5) | 1.1 (0.2-2.6) | 13.6 (2.7-31.4) | -1.87 (-2.09--1.65) |
| Grenada | 2.3 (0.7-4.6) | 24.8 (7.2-49.9) | 1.8 (0.5-3.4) | 13.2 (3.9-25.8) | -2.35 (-2.58--2.12) |
| Guam | 1.4 (0.3-3.2) | 16 (3.8-36.8) | 4.4 (1-10.2) | 19.2 (4.2-44.2) | 0.72 (0.41-1.02) |
| Guatemala | 53.3 (15.9-108.6) | 12.7 (3.8-25.9) | 111.3 (32.8-230) | 8.5 (2.5-17.6) | -1.4 (-1.97--0.84) |
| Guinea | 87.3 (30.3-152.7) | 21.7 (7.5-38) | 100.7 (27.4-209.3) | 16.2 (4.4-33.6) | -0.85 (-0.92--0.78) |
| Guinea-Bissau | 16.8 (7-29.2) | 36.9 (15.3-64.1) | 20.4 (6.2-36.7) | 27 (8.2-48.7) | -0.96 (-1--0.91) |
| Guyana | 8.2 (2.7-18.4) | 18.9 (6.1-42.2) | 10 (3-21.9) | 13.6 (4.1-29.9) | -0.84 (-1.02--0.65) |
| Haiti | 108.9 (30.3-219.6) | 29.1 (8.1-58.6) | 168.7 (46.3-358.2) | 21.7 (5.9-46) | -0.96 (-1.07--0.86) |
| Honduras | 24.3 (7.1-54.3) | 10.4 (3.1-23.2) | 89.7 (26.3-192.2) | 12.7 (3.7-27.2) | 0.95 (0.72-1.18) |
| Hungary | 466 (134.7-928.8) | 24 (6.9-47.7) | 449.2 (116.7-900.1) | 17.6 (4.6-35.3) | -0.85 (-1.06--0.64) |
| Iceland | 7.6 (2-16.1) | 21.2 (5.5-44.8) | 5.2 (1.4-10.7) | 7.5 (2.1-15.6) | -3.65 (-3.79--3.51) |
| India | 4516.8 (2786.1-6400) | 9.1 (5.6-12.8) | 12231.7 (6501.4-18094.1) | 8.9 (4.8-13.2) | 0.12 (-0.03-0.27) |
| Indonesia | 2110.2 (1201-3073.7) | 19.6 (11.1-28.5) | 5080.7 (3133.6-7268.9) | 20.8 (12.8-29.7) | 0.42 (0.14-0.71) |
| Iran (Islamic Republic of) | 896.4 (375.5-1582.8) | 29.3 (12.3-51.7) | 1726.6 (753.9-2955.5) | 20.6 (9-35.2) | -1.2 (-1.45--0.94) |
| Iraq | 310.2 (90.9-621.3) | 35.5 (10.4-71.2) | 893.9 (276.4-1617.9) | 36 (11.1-65.2) | -0.76 (-1.27--0.25) |
| Ireland | 133.8 (33.1-282.3) | 25.2 (6.2-53.2) | 93.7 (18.9-183.9) | 10.1 (2-19.7) | -3.97 (-4.29--3.65) |
| Israel | 114.7 (30.8-221.7) | 18.2 (4.9-35.2) | 87 (18.3-175.3) | 6 (1.3-12.1) | -4.69 (-4.95--4.43) |
| Italy | 1704.6 (777-2652.1) | 14.6 (6.7-22.8) | 1294.4 (567.1-2018.1) | 7.7 (3.4-12) | -2.58 (-2.78--2.38) |
| Jamaica | 15.5 (4.9-33.5) | 6.9 (2.2-14.9) | 19.9 (6.1-42.5) | 5.6 (1.7-12) | -0.56 (-0.88--0.23) |
| Japan | 1734.7 (799.8-2678.5) | 8.1 (3.7-12.5) | 1678.6 (766.1-2595.2) | 4.1 (1.9-6.3) | -2.46 (-2.75--2.17) |
| Jordan | 29.9 (8.7-62.8) | 21.5 (6.3-45.2) | 66 (19.7-139.2) | 9.6 (2.9-20.2) | -3.49 (-3.77--3.21) |
| Kazakhstan | 361.1 (108.7-765.2) | 23.8 (7.2-50.4) | 426.2 (115.6-925.6) | 20.8 (5.6-45.1) | -0.73 (-1.38--0.07) |
| Kenya | 101.4 (49.3-173.8) | 10.9 (5.3-18.7) | 250.6 (109.8-445.2) | 10.6 (4.7-18.9) | 0.04 (-0.12-0.2) |
| Kiribati | 0.6 (0.2-1.6) | 15.7 (4.8-38.3) | 0.9 (0.3-2.1) | 12.3 (3.9-29.2) | -0.81 (-0.92--0.71) |
| Kuwait | 12.2 (3.7-23.3) | 21 (6.4-40.2) | 26.4 (7.2-55.4) | 11.1 (3-23.3) | -1.61 (-1.99--1.23) |
| Kyrgyzstan | 69.4 (22.6-150.4) | 18.7 (6.1-40.5) | 106.9 (35.5-238.8) | 21.1 (7-47.2) | 1.19 (0.59-1.79) |
| Lao People's Democratic Republic | 51.7 (13.9-102.4) | 21.7 (5.8-43) | 75.9 (21.6-160.1) | 15.8 (4.5-33.4) | -1.19 (-1.25--1.13) |
| Latvia | 126.9 (42.8-262.9) | 27.5 (9.3-57) | 105.9 (35.2-222.8) | 20.9 (7-44) | -0.96 (-1.34--0.58) |
| Lebanon | 85.1 (24.9-179.6) | 30.8 (9-65) | 171.8 (49.5-356.8) | 27.3 (7.9-56.8) | 0.49 (0.12-0.87) |
| Lesotho | 6.3 (2-15.6) | 5.4 (1.7-13.3) | 11.1 (3.2-24.8) | 7.6 (2.2-17) | 1.74 (1.47-2.01) |
| Liberia | 30.8 (9.1-56) | 22.5 (6.6-40.9) | 34.3 (9.5-68.1) | 16.5 (4.6-32.7) | -1.45 (-1.62--1.28) |
| Libya | 36.4 (10.5-76.2) | 17.5 (5-36.7) | 97.5 (28.3-206.4) | 18.6 (5.4-39.4) | 0.24 (-0.01-0.48) |
| Lithuania | 156.3 (53.5-329.3) | 27.2 (9.3-57.3) | 150.9 (47.7-307.6) | 20.9 (6.6-42.7) | -0.59 (-1.08--0.1) |
| Luxembourg | 13.8 (3.6-29.1) | 19.7 (5.2-41.4) | 8.9 (2.4-17.9) | 7.4 (2-15) | -3.48 (-3.54--3.41) |
| Madagascar | 63.1 (18.3-143.2) | 10.8 (3.1-24.4) | 111.4 (30.4-264.4) | 10 (2.7-23.7) | -0.49 (-0.74--0.24) |
| Malawi | 37.1 (10.6-83.3) | 8.4 (2.4-18.8) | 47.2 (15-115.5) | 5.8 (1.9-14.3) | -1.54 (-1.65--1.43) |
| Malaysia | 154.7 (56.2-303.7) | 15.1 (5.5-29.7) | 307.5 (118-553.2) | 9.5 (3.6-17.1) | -2.47 (-2.77--2.17) |
| Maldives | 3.8 (1.2-6.8) | 38.7 (12.1-69.7) | 6.4 (2-11.5) | 20.6 (6.4-37.1) | -2.47 (-2.6--2.35) |
| Mali | 109.2 (36.8-190.8) | 22.6 (7.6-39.6) | 116.6 (32.4-253.2) | 12.2 (3.4-26.6) | -2.42 (-2.72--2.12) |
| Malta | 15.7 (2.9-34.4) | 28.9 (5.4-63.3) | 16.1 (3.2-34.8) | 13.3 (2.6-28.8) | -3.26 (-3.55--2.96) |
| Marshall Islands | 0.4 (0.1-0.9) | 20.6 (6-47.5) | 0.6 (0.2-1.4) | 16.6 (5-39) | -0.67 (-0.81--0.53) |
| Mauritania | 31.7 (10.4-56.2) | 27.6 (9-48.8) | 28.2 (7.8-59.8) | 11.7 (3.2-24.9) | -2.95 (-3.25--2.66) |
| Mauritius | 17.9 (5.6-39.1) | 20.2 (6.3-44) | 18.2 (5.4-38.3) | 8.1 (2.4-17.2) | -4.04 (-4.53--3.55) |
| Mexico | 931.9 (437.9-1472.7) | 19 (8.9-30.1) | 2393.1 (1181.9-3732.5) | 17.2 (8.5-26.9) | -0.32 (-0.49--0.14) |
| Micronesia (Federated States of) | 1 (0.3-2.4) | 18.8 (5.3-43.8) | 1.4 (0.4-3.3) | 17.6 (5-41.6) | -0.22 (-0.31--0.13) |
| Monaco | 3.1 (0.5-7) | 34.2 (6-77.2) | 2.2 (0.4-4.9) | 19.2 (3.6-42.4) | -2.29 (-2.55--2.03) |
| Mongolia | 42 (12.8-90.2) | 35.2 (10.7-75.7) | 58.5 (17.3-124.3) | 25 (7.4-53.1) | -2 (-2.54--1.46) |
| Montenegro | 9.4 (3.1-18.9) | 12.4 (4-25) | 17.8 (5.8-35.7) | 13.6 (4.5-27.2) | 0.71 (0.45-0.98) |
| Morocco | 463.2 (167.3-882.2) | 28.8 (10.4-54.9) | 631.2 (267.4-1195.7) | 17.2 (7.3-32.5) | -1.99 (-2.31--1.66) |
| Mozambique | 56.5 (14.7-123.6) | 8.4 (2.2-18.4) | 86.5 (25.7-219.6) | 7.3 (2.2-18.6) | -0.19 (-0.4-0.03) |
| Myanmar | 567.9 (162.7-1068.7) | 21 (6-39.5) | 532.4 (147-1146.1) | 9.9 (2.7-21.3) | -2.83 (-2.94--2.71) |
| Namibia | 16.3 (5.1-30.1) | 18.4 (5.7-33.9) | 31.8 (10.7-57.7) | 20.1 (6.8-36.5) | 0.23 (-0.03-0.48) |
| Nauru | 0.1 (0-0.1) | 17.7 (5.4-40.4) | 0.1 (0-0.1) | 17.8 (5.5-39.7) | -0.05 (-0.85-0.76) |
| Nepal | 192 (58.5-343.7) | 18.5 (5.6-33.1) | 391.1 (99.4-827.3) | 14.5 (3.7-30.8) | -0.93 (-1.08--0.77) |
| Netherlands | 391.4 (108.6-783.5) | 15.5 (4.3-31) | 248.3 (56.5-481.8) | 5.8 (1.3-11.3) | -4.22 (-4.55--3.87) |
| New Zealand | 80.9 (27-153.9) | 16 (5.3-30.4) | 63.6 (19.9-120) | 6.5 (2-12.2) | -3.6 (-3.82--3.37) |
| Nicaragua | 20.6 (5.8-41.1) | 12.1 (3.4-24.1) | 61.3 (18.9-130) | 12 (3.7-25.4) | -0.46 (-0.74--0.18) |
| Niger | 69.7 (21.9-124.3) | 22.8 (7.1-40.6) | 97.6 (26.8-211.2) | 11.4 (3.1-24.6) | -2.9 (-3.29--2.5) |
| Nigeria | 1650.6 (829.4-2725.3) | 32.7 (16.4-54) | 1662.3 (739.9-2891.7) | 18.3 (8.1-31.8) | -2.15 (-2.32--1.97) |
| Niue | 0 (0-0.1) | 17.4 (5.5-40.8) | 0 (0-0.1) | 14.8 (4.5-32.8) | -0.48 (-0.54--0.42) |
| North Macedonia | 40.5 (12.1-87.2) | 17.9 (5.4-38.6) | 69.4 (18.1-158.9) | 16.3 (4.2-37.4) | -0.38 (-0.5--0.26) |
| Northern Mariana Islands | 0.1 (0-0.3) | 9.4 (2.2-22.5) | 0.6 (0.2-1.6) | 10.5 (2.6-25.4) | 0.71 (0.53-0.89) |
| Norway | 221.8 (96.8-341.7) | 25.4 (11.1-39.1) | 85.2 (35.1-136.2) | 7.2 (3-11.6) | -4.85 (-5.08--4.63) |
| Oman | 58.7 (22.5-100.1) | 83.2 (32-142) | 60.6 (19.3-111) | 37.2 (11.9-68.2) | -2.89 (-3.04--2.74) |
| Pakistan | 1857.3 (706.3-3166.5) | 28.6 (10.9-48.8) | 3791.5 (1411.7-6638.3) | 31.8 (11.9-55.7) | 0.46 (0.28-0.64) |
| Palau | 0.2 (0.1-0.4) | 14.4 (4.5-32.5) | 0.3 (0.1-0.7) | 12.5 (3.7-28.1) | -0.57 (-0.69--0.44) |
| Palestine | 56.7 (19.6-99.7) | 55.5 (19.1-97.5) | 99.4 (36.3-165.6) | 39.6 (14.5-66) | -1.28 (-1.45--1.1) |
| Panama | 25.9 (7.5-50) | 14.9 (4.3-28.7) | 36.4 (10.5-71.3) | 7.4 (2.1-14.4) | -2.22 (-2.4--2.05) |
| Papua New Guinea | 21.1 (5.7-51.3) | 10.3 (2.8-25.1) | 75.3 (20.2-171.2) | 15.9 (4.3-36.2) | 1.86 (1.52-2.21) |
| Paraguay | 29.4 (8.8-59.8) | 11.4 (3.4-23.2) | 64.1 (17.8-133) | 9.8 (2.7-20.4) | -0.28 (-0.57-0) |
| Peru | 148.6 (51.9-285.2) | 11 (3.8-21.1) | 147.7 (49.7-279.5) | 3.9 (1.3-7.4) | -3.45 (-4.08--2.82) |
| Philippines | 370 (196.9-575.4) | 11.3 (6-17.5) | 1253.2 (633-1893.9) | 14.1 (7.1-21.3) | 1.28 (0.94-1.61) |
| Poland | 1912.3 (762.4-3161.8) | 33.8 (13.5-55.8) | 1537.2 (551.4-2661.6) | 16.5 (5.9-28.5) | -2.59 (-2.91--2.27) |
| Portugal | 177.6 (62.8-335) | 9.6 (3.4-18.1) | 133.5 (40.7-252) | 4.6 (1.4-8.6) | -3.04 (-3.23--2.85) |
| Puerto Rico | 107.1 (34.3-191.1) | 23.4 (7.5-41.7) | 85.2 (28.3-156.4) | 9.5 (3.1-17.4) | -3.68 (-3.91--3.45) |
| Qatar | 3.3 (0.8-7.2) | 38.8 (9.7-84.2) | 12.7 (3-29) | 14.9 (3.5-34) | -3.18 (-3.78--2.58) |
| Republic of Korea | 299.1 (107.3-653.5) | 9 (3.2-19.7) | 277.9 (103.2-526.9) | 2.4 (0.9-4.6) | -4.38 (-5.05--3.71) |
| Republic of Moldova | 287.2 (84.1-544.4) | 51.5 (15.1-97.6) | 263.3 (76.8-526) | 35 (10.2-69.9) | -1.64 (-2.05--1.22) |
| Romania | 744.9 (269.4-1376.8) | 20.6 (7.5-38.1) | 1075.1 (218.3-2197.2) | 21.8 (4.4-44.6) | 0.25 (0.05-0.46) |
| Russian Federation | 8682.7 (4565-13524) | 37.2 (19.5-57.9) | 7985.2 (4075-12064.2) | 25.6 (13.1-38.7) | -1.55 (-2.02--1.09) |
| Rwanda | 40.8 (10.7-88.8) | 12.3 (3.2-26.7) | 47.1 (13.1-113.1) | 7 (1.9-16.7) | -2.77 (-3.04--2.49) |
| Saint Kitts and Nevis | 1.3 (0.4-2.7) | 25.5 (7.6-51.5) | 0.9 (0.3-1.8) | 11.5 (3.4-22) | -2.64 (-3.03--2.25) |
| Saint Lucia | 1.6 (0.5-3.3) | 14.6 (4.4-29.6) | 1.8 (0.5-3.7) | 7.1 (2.1-14.4) | -2.53 (-2.99--2.08) |
| Saint Vincent and the Grenadines | 1.7 (0.5-3.5) | 18.1 (5.7-38.5) | 1.9 (0.6-3.8) | 11.1 (3.4-22.4) | -1.89 (-2.17--1.6) |
| Samoa | 1.4 (0.5-3.3) | 13.6 (4.5-31.8) | 2.4 (0.7-5.3) | 14.5 (4.4-32.4) | 0.48 (0.35-0.61) |
| San Marino | 0.4 (0.1-0.9) | 10.1 (2.5-22.1) | 0.5 (0.1-1.2) | 7 (1.7-15.8) | -1.34 (-1.39--1.29) |
| Sao Tome and Principe | 1.7 (0.6-2.9) | 21.4 (7.4-36.4) | 2.2 (0.6-4.3) | 19.6 (5.6-38.5) | -0.17 (-0.28--0.07) |
| Saudi Arabia | 130.8 (36.5-251.4) | 21.4 (6-41.2) | 354.2 (66.7-693.9) | 21.9 (4.1-43) | 0.2 (-0.03-0.44) |
| Senegal | 118.7 (83.9-156.9) | 31.9 (22.5-42.1) | 216.9 (88.6-366.7) | 25.5 (10.4-43.1) | -0.69 (-0.8--0.58) |
| Serbia | 275.8 (77.6-603) | 18.7 (5.3-40.9) | 336.5 (100.9-703) | 15.3 (4.6-32.1) | -0.51 (-0.73--0.29) |
| Seychelles | 2.1 (0.8-3.5) | 30.1 (11.2-49.7) | 2.5 (0.9-4.3) | 19.7 (7.3-33.5) | -1.39 (-1.65--1.13) |
| Sierra Leone | 92.4 (42.4-150.9) | 41 (18.8-66.9) | 106.6 (37.2-189.9) | 27.3 (9.5-48.6) | -1.35 (-1.46--1.25) |
| Singapore | 29.5 (11.3-57.3) | 11.7 (4.5-22.7) | 36.5 (13-63.5) | 3.7 (1.3-6.4) | -4.22 (-4.4--4.03) |
| Slovakia | 223.5 (77.7-418.8) | 28.8 (10-53.9) | 197.5 (61.8-390.5) | 16.1 (5-31.9) | -1.78 (-2.22--1.34) |
| Slovenia | 37.6 (10.9-80.6) | 12.2 (3.5-26.1) | 37 (9.3-81.3) | 6.8 (1.7-15) | -2.34 (-2.52--2.16) |
| Solomon Islands | 6.1 (1.6-12.7) | 42.1 (11-87.2) | 10.8 (3-24) | 35.6 (9.9-78.9) | -0.57 (-0.71--0.43) |
| Somalia | 28.3 (7.1-63.7) | 11.6 (2.9-26.2) | 81.4 (20.7-183.8) | 11.4 (2.9-25.8) | 0.01 (-0.07-0.09) |
| South Africa | 189.5 (83.6-308.3) | 7.8 (3.5-12.7) | 396.7 (159.3-631.6) | 7.5 (3-12) | -0.12 (-0.51-0.28) |
| South Sudan | 18.3 (5.4-43.7) | 6.7 (2-16.1) | 21.8 (6.6-52.4) | 5.5 (1.7-13.2) | -0.65 (-0.71--0.58) |
| Spain | 749.4 (208.7-1518.8) | 10.4 (2.9-21.1) | 660.2 (156.9-1324.2) | 5.9 (1.4-11.8) | -2.31 (-2.47--2.15) |
| Sri Lanka | 185.4 (53.9-414.4) | 14.9 (4.3-33.4) | 290.6 (83.7-649.1) | 8.8 (2.5-19.7) | -1.57 (-1.75--1.39) |
| Sudan | 470.5 (126.9-937.7) | 44.1 (11.9-87.9) | 638 (169.5-1317.4) | 32.2 (8.6-66.5) | -1.31 (-1.41--1.2) |
| Suriname | 4.6 (1.4-9.4) | 15.2 (4.7-31.5) | 7.1 (2.1-14.7) | 10 (3-20.8) | -1.36 (-1.79--0.92) |
| Sweden | 428.7 (160.6-743.8) | 22.4 (8.4-38.9) | 214.4 (66.8-387.8) | 8.5 (2.6-15.3) | -3.74 (-3.88--3.59) |
| Switzerland | 173.1 (57.8-331.4) | 13.4 (4.5-25.7) | 114.4 (35.2-216.1) | 5.5 (1.7-10.5) | -3.45 (-3.63--3.28) |
| Syrian Arab Republic | 144.6 (47.2-333.9) | 24.6 (8-56.9) | 375.6 (114.3-828.9) | 26.1 (7.9-57.5) | 0.04 (-0.12-0.19) |
| Taiwan (Province of China) | 135.4 (48.1-266.8) | 6.8 (2.4-13.4) | 150.4 (55.6-287.5) | 2.9 (1.1-5.6) | -2.7 (-2.87--2.52) |
| Tajikistan | 156.4 (54.1-260.7) | 47.9 (16.6-79.9) | 295.4 (89.7-529.6) | 56.7 (17.2-101.7) | 1.34 (0.79-1.89) |
| Thailand | 266.6 (92.3-531.2) | 6.7 (2.3-13.4) | 485.7 (145.1-952.8) | 3.8 (1.1-7.5) | -1.94 (-2.31--1.57) |
| Timor-Leste | 3.5 (0.9-7) | 12.8 (3.4-25.9) | 26.5 (8.8-47.1) | 25.5 (8.5-45.4) | 2.66 (2.53-2.79) |
| Togo | 45.6 (21.5-72.7) | 34.3 (16.2-54.7) | 71.7 (20.3-136.3) | 18.7 (5.3-35.6) | -2.34 (-2.53--2.15) |
| Tokelau | 0 (0-0.1) | 18.2 (5.2-39.1) | 0 (0-0) | 12.9 (4-29.2) | -1.36 (-1.77--0.94) |
| Tonga | 0.7 (0.2-1.6) | 10.6 (3.1-24.8) | 1 (0.3-2.3) | 10.5 (3.2-24.8) | 0.09 (-0.13-0.3) |
| Trinidad and Tobago | 20.3 (6.2-41) | 19.7 (6-39.8) | 28.5 (8.2-59.3) | 12 (3.5-24.9) | -2.4 (-2.67--2.13) |
| Tunisia | 96.5 (31.2-215.2) | 15.9 (5.1-35.3) | 234.8 (65.5-529.7) | 15.2 (4.2-34.2) | -0.16 (-0.33-0.01) |
| Turkey | 607.7 (217.9-1167.4) | 14.7 (5.3-28.3) | 1036.2 (296.4-2013.3) | 9.7 (2.8-18.9) | -1.34 (-1.8--0.88) |
| Turkmenistan | 67.7 (21.4-138.7) | 30.4 (9.6-62.4) | 135.3 (35.3-312.5) | 31.4 (8.2-72.6) | -0.56 (-0.86--0.27) |
| Tuvalu | 0.2 (0.1-0.4) | 25.4 (6.7-52.5) | 0.3 (0.1-0.6) | 20.1 (5.9-43.5) | -0.62 (-0.72--0.52) |
| Uganda | 61.6 (16.1-145.4) | 8.4 (2.2-19.8) | 119.2 (31.7-278.9) | 8 (2.1-18.8) | -0.2 (-0.32--0.09) |
| Ukraine | 3297.4 (1319.3-6449.2) | 34.7 (13.9-67.9) | 4571.8 (1969.8-8479.4) | 45.5 (19.6-84.4) | 0.2 (-0.36-0.75) |
| United Arab Emirates | 8.6 (1.8-17.4) | 27.4 (5.8-55.6) | 26.4 (6.4-53.3) | 9.9 (2.4-19.9) | -3.94 (-4.64--3.23) |
| United Kingdom | 4592 (2623.8-6596.3) | 39.1 (22.4-56.2) | 1905 (1019.8-2725.1) | 12.5 (6.7-17.9) | -4.76 (-5.1--4.43) |
| United Republic of Tanzania | 89.3 (25.9-213.4) | 7.1 (2.1-16.9) | 223.7 (63.7-530.8) | 8.4 (2.4-20) | 0.65 (0.51-0.8) |
| United States of America | 10869.1 (4344.7-17777.3) | 26.5 (10.6-43.4) | 10053.5 (3501.1-17561.4) | 14.1 (4.9-24.6) | -2.81 (-3.08--2.54) |
| United States Virgin Islands | 2 (0.6-3.8) | 20.5 (6.2-39.3) | 4.6 (1.4-9.3) | 18.3 (5.4-36.9) | -0.11 (-0.39-0.17) |
| Uruguay | 87.7 (22.2-191.6) | 17.2 (4.4-37.7) | 80.4 (15.7-180.3) | 12 (2.4-27) | -1.34 (-1.52--1.15) |
| Uzbekistan | 339.2 (109.9-729.2) | 25.6 (8.3-55) | 838.9 (260.4-1815.9) | 37.7 (11.7-81.5) | 1.79 (0.88-2.7) |
| Vanuatu | 1.5 (0.4-3.5) | 21.1 (5.9-48.4) | 4.3 (1.3-10) | 21.2 (6.4-49.8) | -0.17 (-0.43-0.09) |
| Venezuela (Bolivarian Republic of) | 153.2 (52.8-278.7) | 13.9 (4.8-25.2) | 351.4 (128.8-639.2) | 9.9 (3.6-18.1) | -1.51 (-1.8--1.22) |
| Viet Nam | 908.3 (396.3-1453.8) | 18.5 (8.1-29.6) | 1025.9 (382.7-1914.2) | 9.6 (3.6-17.9) | -2.29 (-2.47--2.1) |
| Yemen | 323.3 (109.2-568) | 59 (19.9-103.7) | 808.4 (290.4-1358.2) | 55.6 (20-93.5) | -0.66 (-0.81--0.52) |
| Zambia | 39.3 (10.3-80.3) | 12.8 (3.4-26.1) | 76.6 (20.5-153.7) | 11.2 (3-22.4) | -0.49 (-0.88--0.11) |
| Zimbabwe | 73.2 (20.8-146.4) | 15.6 (4.4-31.3) | 122.1 (35.3-265.2) | 15.6 (4.5-33.9) | -0.12 (-0.37-0.13) |

Abbreviations: EAPC, estimated annual percentage change; SDI, Sociodemographic Index; UI, uncertainty interval.

**Table S2.** DALY of Cardiovascular diseases between 1990 and 2019 in the old at the global and regional level.

| Location | 1990 | |  | 2019 | | EAPC_95%CI |
| --- | --- | --- | --- | --- | --- | --- |
|  | Number_95%UI | ASR |  | Number_95%UI | ASR |  |
| Global | 1660754 (1166683-2099667) | 345.8 (242.9-437.2) |  | 2235041.3 (1469065.4-2920743.1) | 220.6 (145-288.3) | -1.66 (-1.74--1.58) |
| High SDI | 457668.6 (219995.7-661292.1) | 343.5 (165.1-496.4) |  | 352128.5 (141372.4-545723.6) | 150.5 (60.4-233.2) | -3.29 (-3.52--3.05) |
| High-middle SDI | 523641.3 (337757.4-691165.6) | 390.7 (252-515.7) |  | 623647.9 (366795.9-851533.6) | 240.8 (141.6-328.7) | -1.96 (-2.24--1.69) |
| Middle SDI | 386431.7 (286169.5-493869.7) | 325.8 (241.3-416.4) |  | 717120.1 (467785.7-970265) | 237.7 (155-321.6) | -0.91 (-1.06--0.77) |
| Low-middle SDI | 195503.9 (146975.8-247627.5) | 288.9 (217.2-365.9) |  | 374326.6 (254744.3-492341.8) | 232.7 (158.4-306.1) | -0.62 (-0.71--0.53) |
| Low SDI | 96695.1 (72774-124309.6) | 365.4 (275-469.7) |  | 166643.8 (119858-218595) | 292.1 (210.1-383.2) | -0.82 (-0.88--0.76) |
| Andean Latin America | 4247.9 (1745.9-7036.6) | 181.6 (74.6-300.8) |  | 6534.1 (2199.3-11713.1) | 98.2 (33-176) | -1.92 (-2.39--1.44) |
| Australasia | 9404.6 (2487.2-17860.1) | 309.5 (81.8-587.7) |  | 5518.8 (1454.4-10175.9) | 89.4 (23.6-164.8) | -4.71 (-5.02--4.4) |
| Caribbean | 10496.8 (5289.4-16236.8) | 330.9 (166.7-511.8) |  | 14822.3 (6841.4-23740.3) | 237.2 (109.5-379.9) | -1.16 (-1.37--0.95) |
| Central Asia | 29980.8 (16705.5-43753.7) | 538.6 (300.1-786) |  | 45556.9 (22243.6-71325.5) | 545.4 (266.3-853.9) | -0.09 (-0.57-0.39) |
| Central Europe | 87694.5 (34535.7-134510.7) | 458.7 (180.7-703.6) |  | 80301.5 (24972.3-129638.8) | 281.8 (87.6-454.9) | -1.78 (-1.97--1.58) |
| Central Europe, Eastern Europe, and Central Asia | 338388.6 (202478.7-465362.3) | 554.1 (331.6-762) |  | 353695.9 (200030.5-485769.3) | 429 (242.6-589.1) | -1.32 (-1.72--0.92) |
| Central Latin America | 26622.2 (13955.7-38557.2) | 279.2 (146.3-404.3) |  | 62012.7 (30370.4-92750.8) | 220.2 (107.8-329.3) | -0.85 (-1.03--0.67) |
| Central Sub-Saharan Africa | 6291 (3388.6-10827) | 248.8 (134-428.2) |  | 12402.7 (5433.2-23051) | 224.2 (98.2-416.7) | -0.35 (-0.47--0.22) |
| East Asia | 311528.1 (186842.3-452280.4) | 298.5 (179-433.3) |  | 531682.5 (297093-816275.1) | 202 (112.9-310.2) | -0.96 (-1.24--0.68) |
| Eastern Sub-Saharan Africa | 20184.7 (12822.4-28873.2) | 241.1 (153.2-344.9) |  | 32517 (19955.7-47341.9) | 186.2 (114.3-271.1) | -1 (-1.05--0.96) |
| High-income Asia Pacific | 33598.9 (16478.5-49858.9) | 133.9 (65.7-198.7) |  | 29115.3 (13645.3-43372.3) | 54.1 (25.4-80.6) | -3.15 (-3.46--2.85) |
| High-income North America | 196210.5 (80233.5-317642) | 434.9 (177.8-704) |  | 186156.6 (63449.9-320714) | 232.2 (79.2-400.1) | -2.75 (-2.99--2.51) |
| North Africa and Middle East | 110588.1 (70957.7-149421.3) | 563.6 (361.7-761.6) |  | 179872.1 (107026.6-250282.1) | 370.7 (220.6-515.8) | -1.62 (-1.71--1.52) |
| Oceania | 958.3 (449.2-1727.7) | 293.2 (137.4-528.5) |  | 2547.4 (1021.6-4771.5) | 348 (139.6-651.8) | 0.84 (0.7-0.97) |
| South Asia | 153331.8 (104851.9-205956.2) | 244.9 (167.5-329) |  | 363456.8 (222050.3-506431.4) | 217.6 (132.9-303.2) | -0.23 (-0.33--0.13) |
| Southeast Asia | 89422 (63398.9-117354.6) | 310.5 (220.1-407.4) |  | 170724.1 (117798.3-224991.3) | 240.5 (166-317) | -0.83 (-1--0.66) |
| Southern Latin America | 17570 (4534.2-32823) | 301.7 (77.9-563.6) |  | 19444.5 (3773.1-34903.5) | 186.5 (36.2-334.7) | -1.79 (-2--1.58) |
| Southern Sub-Saharan Africa | 5527.7 (2951-8137.3) | 173.2 (92.4-254.9) |  | 10934.5 (5431-16295.8) | 166 (82.5-247.4) | -0.15 (-0.47-0.18) |
| Tropical Latin America | 36955.1 (18422.4-54121.1) | 347 (173-508.1) |  | 59690.8 (31618.1-84182.6) | 202.6 (107.3-285.7) | -1.77 (-1.82--1.71) |
| Western Europe | 235565.9 (113223.3-342176.5) | 315.9 (151.9-458.9) |  | 131639.3 (52139.3-198946.1) | 120.2 (47.6-181.7) | -3.76 (-4.02--3.49) |
| Western Sub-Saharan Africa | 53861.6 (37067-75701.8) | 537.6 (370-755.6) |  | 62273.7 (40850.3-87388.7) | 309.5 (203.1-434.4) | -2.02 (-2.16--1.87) |

Abbreviations:DALY, Disability-Adjusted Life Years; EAPC, Estimated annual percentage change; SDI, Sociodemographic Index; UI, Uncertainty interval.

**Table S3.** DALY of Cardiovascular diseases between 1990 and 2019 in the old at the 204 Countries Level.

| Location | 1990 | | 2019 | | EAPC_95%CI |
| --- | --- | --- | --- | --- | --- |
|  | Number_95%UI | ASR | Number_95%UI | ASR |  |
| Afghanistan | 8165.8 (2228.1-15960) | 947.9 (258.6-1852.7) | 9592.4 (2715.4-17956.9) | 890.1 (252-1666.2) | -0.82 (-1.27--0.37) |
| Albania | 475.8 (143.9-1028.4) | 199.6 (60.4-431.5) | 1409.3 (287.8-3251.5) | 249.4 (50.9-575.5) | 1.47 (1.01-1.95) |
| Algeria | 9887.7 (2802.3-19334.7) | 673.7 (190.9-1317.4) | 11878.6 (3477.3-25102.5) | 302.3 (88.5-638.7) | -3.16 (-3.4--2.92) |
| American Samoa | 5.4 (1.5-11.9) | 218.7 (61.3-477.7) | 12.5 (3.5-27.2) | 225.6 (63-489.5) | 0.14 (0.06-0.22) |
| Andorra | 12 (2.6-28.6) | 168.1 (37.2-402.3) | 17.4 (3.8-39.4) | 104.4 (22.6-236.7) | -1.64 (-1.71--1.56) |
| Angola | 1877.8 (643.6-3279.9) | 463.9 (159-810.3) | 3057.5 (874-6116.4) | 266.7 (76.2-533.6) | -2.37 (-2.53--2.2) |
| Antigua and Barbuda | 28.6 (9.4-49.5) | 427.1 (140.4-740.5) | 30.9 (10.8-53.4) | 253.1 (88.4-438.1) | -2.09 (-2.3--1.89) |
| Argentina | 13577 (3073.6-27052.3) | 330.4 (74.8-658.4) | 14554.1 (2447.9-27906.1) | 215.2 (36.2-412.6) | -1.63 (-1.84--1.42) |
| Armenia | 2682.7 (940.8-4440.4) | 820 (287.5-1357.2) | 2429.2 (790.7-4588.4) | 443.2 (144.3-837.2) | -2.76 (-2.99--2.54) |
| Australia | 7999.2 (1955.1-15818.9) | 316 (77.2-624.8) | 4544.1 (1097.7-8834.5) | 87.5 (21.1-170.1) | -4.84 (-5.15--4.52) |
| Austria | 3788.6 (1280.2-6909.8) | 247.6 (83.7-451.5) | 2827.4 (756.1-5468.4) | 132.9 (35.5-257) | -2.46 (-2.61--2.31) |
| Azerbaijan | 3965.8 (1125.7-7960.3) | 706.4 (200.5-1418) | 6328.2 (1875.4-13136.7) | 594.8 (176.3-1234.8) | -1.03 (-1.32--0.74) |
| Bahamas | 63.2 (20.9-111.4) | 365.9 (121.3-645.1) | 111.5 (36.9-199.2) | 245.4 (81.2-438.3) | -1.5 (-1.71--1.29) |
| Bahrain | 133 (39.3-268.1) | 740 (218.5-1492.4) | 211.9 (56.6-434.5) | 202.5 (54.1-415.2) | -5.09 (-5.5--4.68) |
| Bangladesh | 25149.9 (10484.7-41267.4) | 482 (200.9-790.9) | 54910.4 (15817.1-100282.6) | 351.1 (101.1-641.2) | -0.55 (-0.81--0.3) |
| Barbados | 77.8 (23.2-158.9) | 206.9 (61.7-422.2) | 75.3 (21.6-152.5) | 117.9 (33.9-238.8) | -2.43 (-2.68--2.18) |
| Belarus | 10004.8 (2861.2-20536) | 595.4 (170.3-1222.1) | 15604.7 (4465.3-30957.5) | 751.1 (214.9-1490) | 1.07 (0.45-1.69) |
| Belgium | 4811.6 (1336.4-9406.4) | 240.6 (66.8-470.4) | 3165.4 (691.2-6166.7) | 114.6 (25-223.2) | -2.73 (-2.88--2.58) |
| Belize | 25.6 (7.8-54.4) | 231.3 (70-490.5) | 41.8 (12.7-87.2) | 136.9 (41.6-285.4) | -2.38 (-2.83--1.92) |
| Benin | 1272.7 (569.4-2089.7) | 549.4 (245.8-902.2) | 1470.6 (410.7-2895.4) | 281.6 (78.6-554.4) | -2.48 (-2.76--2.19) |
| Bermuda | 47 (15-85.5) | 608.2 (194.8-1107.1) | 40.2 (14.7-68.7) | 243.1 (88.7-415) | -3.27 (-3.51--3.03) |
| Bhutan | 105.7 (33.6-192.7) | 399.2 (127-727.7) | 219 (58.1-428.7) | 331.7 (88.1-649.3) | -0.57 (-0.66--0.47) |
| Bolivia (Plurinational State of) | 770.6 (223.9-1693.8) | 208.7 (60.6-458.7) | 1703.6 (465.2-3796.4) | 160.3 (43.8-357.2) | -0.92 (-1.16--0.67) |
| Bosnia and Herzegovina | 1129.4 (374.8-2519.5) | 236.2 (78.4-526.9) | 1840.1 (464.9-4250.3) | 231 (58.4-533.6) | -0.21 (-0.31--0.12) |
| Botswana | 225.8 (68.7-424.7) | 338.2 (102.9-636.3) | 527.3 (161.1-1007) | 350.7 (107.2-669.8) | -0.14 (-0.55-0.27) |
| Brazil | 36431.1 (18044.7-53564.7) | 350.5 (173.6-515.4) | 58520.2 (30794.3-82830.7) | 203.1 (106.9-287.5) | -1.79 (-1.85--1.74) |
| Brunei Darussalam | 53.1 (17.8-93.3) | 534.1 (178.9-938.6) | 81.9 (26.1-146.1) | 249.4 (79.3-444.6) | -2.51 (-2.72--2.3) |
| Bulgaria | 9548.8 (2208.2-19803.8) | 568.6 (131.5-1179.3) | 7533.3 (1764.1-15194.3) | 390 (91.3-786.6) | -1.76 (-2.11--1.41) |
| Burkina Faso | 2435.2 (1095.8-3994.7) | 474.5 (213.5-778.4) | 3355.9 (926.3-6460.9) | 339.6 (93.7-653.7) | -1.16 (-1.3--1.02) |
| Burundi | 708.8 (189.3-1599.9) | 265.9 (71-600.2) | 915.1 (245.5-2197.1) | 186.9 (50.1-448.7) | -1.39 (-1.46--1.32) |
| Cabo Verde | 177.5 (86.7-274.3) | 611.7 (298.7-945.2) | 246.9 (95.5-417) | 510.7 (197.6-862.5) | -1.12 (-1.42--0.82) |
| Cambodia | 1468.3 (379.3-2960.1) | 293.9 (75.9-592.4) | 2689.8 (740.2-5860.5) | 196.5 (54.1-428.1) | -1.56 (-1.7--1.42) |
| Cameroon | 1970.9 (799.4-3330.2) | 395.2 (160.3-667.7) | 3853.3 (1121.7-7599.9) | 301.7 (87.8-595.1) | -1.05 (-1.27--0.83) |
| Canada | 13184.7 (3957.7-26532.8) | 318 (95.4-639.9) | 11177.9 (2741-23859.8) | 127.9 (31.4-272.9) | -3.65 (-3.87--3.43) |
| Central African Republic | 432 (113.9-911.1) | 346.5 (91.4-730.8) | 557 (151-1290.2) | 260.6 (70.7-603.7) | -1.1 (-1.29--0.91) |
| Chad | 2067 (1077.6-3269) | 624.7 (325.7-988) | 2779.4 (986.3-4832.7) | 459 (162.9-798.1) | -1.07 (-1.16--0.98) |
| Chile | 2479.3 (743-4727.6) | 205.6 (61.6-392) | 3549 (769.5-7077.7) | 118.4 (25.7-236.2) | -1.92 (-2.18--1.66) |
| China | 302728.7 (177388.6-443189.6) | 300.9 (176.3-440.5) | 514504.5 (280334.1-798182.4) | 202.4 (110.3-314) | -0.98 (-1.27--0.69) |
| Colombia | 4428 (1622.9-8171.1) | 222.5 (81.6-410.7) | 7247.2 (2124.6-13905.2) | 113.5 (33.3-217.8) | -2.19 (-2.59--1.78) |
| Comoros | 41.7 (12-98.1) | 160.9 (46.3-379.1) | 75.6 (23.2-177.4) | 136.8 (42-320.8) | -0.56 (-0.67--0.46) |
| Congo | 542.7 (158-1046.9) | 444.8 (129.5-858.1) | 843 (243.1-1733) | 314.1 (90.6-645.8) | -1.35 (-1.53--1.17) |
| Cook Islands | 2.5 (0.8-5.8) | 170.8 (52.8-391.4) | 4.6 (1.1-11.2) | 149.5 (34.2-363.7) | -0.54 (-0.74--0.34) |
| Costa Rica | 441.3 (122.1-918.9) | 215.9 (59.7-449.5) | 870.7 (217.6-1890.1) | 139.6 (34.9-303) | -1.83 (-2.05--1.6) |
| Coted'Ivoire | 1673.8 (502.1-3086.7) | 404.3 (121.3-745.7) | 2682.2 (740.3-5632) | 244.7 (67.5-513.8) | -1.99 (-2.16--1.82) |
| Croatia | 3014.3 (828-6482.3) | 366.4 (100.6-787.9) | 2929.7 (610.2-6772.3) | 256 (53.3-591.9) | -0.61 (-0.89--0.33) |
| Cuba | 3924.6 (1165.6-8284.8) | 309.5 (91.9-653.4) | 4672.1 (1084.6-10705.9) | 206.6 (48-473.3) | -1.42 (-1.76--1.09) |
| Cyprus | 315.8 (85.1-683.8) | 295.1 (79.5-639.2) | 326.8 (96.4-670.8) | 129.7 (38.3-266.3) | -3.68 (-3.94--3.42) |
| Czechia | 10627.4 (2721.8-23381.7) | 585.1 (149.9-1287.3) | 7303.2 (1735.9-16414) | 268.2 (63.7-602.7) | -2.89 (-3.02--2.76) |
| Democratic People's Republic of Korea | 6260 (2326.5-10920.8) | 353.9 (131.5-617.5) | 14652.3 (4455.4-26439.2) | 383.3 (116.5-691.6) | 0.54 (0.4-0.67) |
| Democratic Republic of the Congo | 3134.9 (969.5-7199) | 175.4 (54.2-402.7) | 7593.7 (2077.8-17506.6) | 203.2 (55.6-468.4) | 0.65 (0.35-0.94) |
| Denmark | 3638.6 (1082.5-7350.8) | 354.8 (105.5-716.7) | 1289.1 (341.5-2632.9) | 90 (23.8-183.9) | -5.69 (-6.07--5.31) |
| Djibouti | 47.2 (15.9-84.4) | 343 (115.9-613.8) | 140.4 (37.2-308.7) | 228.4 (60.5-502.1) | -1.62 (-1.77--1.48) |
| Dominica | 20.5 (6.2-42.8) | 216.6 (65.9-452) | 15.6 (4.5-34.1) | 138.8 (40.3-304) | -1.63 (-1.84--1.42) |
| Dominican Republic | 928 (273.6-1938.6) | 215.7 (63.6-450.6) | 3198.6 (932.8-6724.1) | 293 (85.5-616) | 1.6 (1.43-1.77) |
| Ecuador | 984 (255.3-2148.7) | 159.8 (41.5-349) | 2461.4 (575.7-5595.4) | 135 (31.6-307) | -0.38 (-0.74--0.03) |
| Egypt | 20397.9 (7463.1-40330.8) | 622.2 (227.6-1230.1) | 28413.1 (10905-50882.1) | 392 (150.4-702) | -1.25 (-1.73--0.76) |
| El Salvador | 633.2 (196.4-1323) | 184.1 (57.1-384.7) | 1165.9 (332.5-2455.4) | 164.6 (47-346.8) | -0.37 (-0.52--0.23) |
| Equatorial Guinea | 71.7 (19.3-152.3) | 327.2 (88-694.8) | 93.9 (26.5-194.9) | 185.4 (52.4-385) | -2.39 (-2.73--2.05) |
| Eritrea | 164.2 (42.3-384.6) | 174.6 (45-409.1) | 425.3 (128.5-1036.2) | 159.1 (48.1-387.6) | -0.24 (-0.3--0.18) |
| Estonia | 1156.4 (401.3-2373.5) | 438.3 (152.1-899.5) | 675.7 (201.3-1407.2) | 201.8 (60.1-420.4) | -3.26 (-3.49--3.03) |
| Eswatini | 66.8 (18.7-146) | 212.6 (59.6-464.9) | 153.6 (41.9-324.4) | 234.6 (64-495.4) | 0.57 (0.25-0.89) |
| Ethiopia | 9079.9 (3587.2-16248.8) | 415 (164-742.7) | 10813.6 (4131.9-20057.6) | 240.3 (91.8-445.7) | -2.15 (-2.27--2.03) |
| Fiji | 151.8 (45.8-353.1) | 412.3 (124.5-959.1) | 293 (85.2-652.9) | 339.2 (98.6-755.8) | -0.66 (-0.77--0.54) |
| Finland | 3205.5 (1054.4-5886.1) | 349.4 (114.9-641.6) | 2338.4 (678.3-4330.8) | 152.2 (44.2-281.9) | -2.92 (-3.16--2.67) |
| France | 13794.1 (4461.9-26681.9) | 129.9 (42-251.3) | 9425.3 (2756-18081.1) | 57.3 (16.8-110) | -2.95 (-3.11--2.78) |
| Gabon | 231.9 (70.9-449) | 343.4 (105-664.8) | 257.6 (73.6-540.5) | 223 (63.7-468) | -1.27 (-1.39--1.15) |
| Gambia | 169.5 (52.7-304.6) | 437.6 (136.2-786.7) | 389.2 (106.7-783.4) | 367.2 (100.7-739.2) | -0.71 (-0.92--0.51) |
| Georgia | 5282.2 (1529.7-10976.4) | 664.4 (192.4-1380.6) | 2555.8 (760.1-5429.3) | 330.8 (98.4-702.6) | -2.93 (-3.2--2.66) |
| Germany | 57918 (15683.4-111814.3) | 362.6 (98.2-700) | 35214.4 (8367.4-67853.5) | 153.1 (36.4-295) | -3.18 (-3.54--2.81) |
| Ghana | 3336.9 (1440.1-5480.9) | 484.3 (209-795.5) | 3921.3 (1112.6-8469.6) | 220.5 (62.5-476.2) | -2.77 (-3.1--2.44) |
| Greece | 4099.9 (1231.8-7922.9) | 207.3 (62.3-400.5) | 3718.8 (1170.9-7044.1) | 131.9 (41.5-249.8) | -1.67 (-1.84--1.5) |
| Greenland | 15.4 (3.7-33.5) | 427.5 (103.6-933.7) | 22.1 (4.2-51.1) | 264.5 (50.6-611.9) | -1.91 (-2.08--1.73) |
| Grenada | 38 (10.8-76) | 411.6 (117.4-823) | 33.6 (9.8-65.3) | 253.1 (74.1-491.4) | -1.69 (-1.93--1.45) |
| Guam | 29 (6.7-67.3) | 331.4 (77-769.1) | 85.6 (18.1-198.8) | 370.6 (78.5-860.6) | 0.48 (0.16-0.8) |
| Guatemala | 962.8 (284.4-1941.6) | 229.8 (67.9-463.5) | 1776.5 (519.6-3687.9) | 135.6 (39.6-281.4) | -1.83 (-2.39--1.26) |
| Guinea | 1570.5 (553.1-2746.9) | 390.4 (137.5-682.8) | 1818 (494.2-3780.7) | 292.1 (79.4-607.5) | -0.93 (-0.96--0.9) |
| Guinea-Bissau | 329 (135.7-574.4) | 721.3 (297.4-1259.3) | 395.1 (118.5-715.8) | 523.2 (156.9-948) | -0.95 (-1--0.9) |
| Guyana | 159.7 (51-356.7) | 367.3 (117.4-820.2) | 198.8 (58.8-439.6) | 272 (80.4-601.3) | -0.77 (-0.93--0.61) |
| Haiti | 2178.9 (606.1-4371.6) | 581.3 (161.7-1166.2) | 3260.1 (898.4-6840.5) | 418.6 (115.4-878.3) | -1.1 (-1.24--0.97) |
| Honduras | 434.4 (126-964.3) | 185.8 (53.9-412.4) | 1585.1 (458.7-3363) | 224 (64.8-475.3) | 0.96 (0.71-1.2) |
| Hungary | 8117.2 (2295.5-16063.3) | 417.2 (118-825.5) | 7111.3 (1800.2-14004.5) | 278.7 (70.5-548.8) | -1.23 (-1.46--0.99) |
| Iceland | 126 (32.1-269.1) | 351.2 (89.6-749.8) | 81 (21.7-169.3) | 118 (31.6-246.7) | -3.93 (-4.01--3.84) |
| India | 89668.9 (54410.4-127541.2) | 180 (109.2-256) | 227039.5 (115894.7-340180.3) | 166 (84.8-248.8) | -0.16 (-0.32-0) |
| Indonesia | 41561.8 (24227.1-59474.9) | 385.6 (224.8-551.8) | 95477.5 (60015.3-134746.2) | 390.4 (245.4-551) | 0.21 (-0.06-0.47) |
| Iran (Islamic Republic of) | 18060.9 (7509.7-31853.3) | 590.3 (245.5-1041.2) | 28807.5 (12544.8-49205.2) | 342.9 (149.3-585.8) | -2.04 (-2.26--1.81) |
| Iraq | 5686.4 (1663.6-11378) | 651.4 (190.6-1303.5) | 16510.6 (5115.3-30002.2) | 665.2 (206.1-1208.8) | -0.74 (-1.25--0.22) |
| Ireland | 2325 (566.3-4867.6) | 438.3 (106.8-917.6) | 1508.8 (289.1-2930) | 162 (31-314.5) | -4.2 (-4.55--3.85) |
| Israel | 1935 (508.2-3731.9) | 307.1 (80.7-592.3) | 1355.9 (273.7-2661.7) | 93.5 (18.9-183.6) | -4.98 (-5.3--4.66) |
| Italy | 27341.8 (12368.8-41931.6) | 234.9 (106.3-360.3) | 18021.8 (7866.9-27816.5) | 107 (46.7-165.1) | -3.08 (-3.31--2.86) |
| Jamaica | 260.7 (80.5-561.6) | 116.3 (35.9-250.5) | 353.7 (107.3-753) | 99.8 (30.3-212.5) | -0.39 (-0.71--0.08) |
| Japan | 27835.3 (12863.7-42323.2) | 129.4 (59.8-196.7) | 24245.1 (11029.7-36702.4) | 58.6 (26.6-88.7) | -2.81 (-3.08--2.54) |
| Jordan | 570.8 (164.7-1198.7) | 410.8 (118.5-862.7) | 1249.5 (368.9-2624.3) | 181 (53.4-380.1) | -3.59 (-3.88--3.3) |
| Kazakhstan | 6431.6 (1885.1-13537.1) | 423.8 (124.2-892) | 7356.3 (1951.2-15883.8) | 358.7 (95.1-774.5) | -1.03 (-1.72--0.34) |
| Kenya | 1862.8 (902.1-3191.3) | 200.8 (97.3-344.1) | 4793 (2116.1-8412.1) | 203.1 (89.7-356.4) | 0.18 (-0.01-0.36) |
| Kiribati | 14.4 (4.4-34.9) | 351.6 (107.2-851.1) | 20 (6.3-47.2) | 272.4 (86.1-643) | -0.84 (-0.99--0.68) |
| Kuwait | 241.4 (73.3-459.7) | 416.3 (126.4-792.7) | 513 (136.6-1076.7) | 215.9 (57.5-453) | -1.88 (-2.24--1.52) |
| Kyrgyzstan | 1211.2 (387.3-2596) | 326.1 (104.3-698.9) | 1786.4 (580.1-3966.9) | 353.2 (114.7-784.3) | 0.73 (0.2-1.26) |
| Lao People's Democratic Republic | 1027.4 (275-2030.6) | 431.6 (115.5-853) | 1392.1 (396.9-2948.8) | 290.3 (82.8-615) | -1.48 (-1.52--1.44) |
| Latvia | 2056.6 (692.1-4202.4) | 446.3 (150.2-912) | 1588.2 (529-3281.9) | 313.8 (104.5-648.4) | -1.41 (-1.72--1.09) |
| Lebanon | 1567.2 (456.1-3292.5) | 567 (165-1191.3) | 2833.9 (815.2-5827.6) | 451 (129.7-927.5) | -0.03 (-0.37-0.31) |
| Lesotho | 121.9 (38.4-298.8) | 104.2 (32.9-255.4) | 221.5 (62.3-491.7) | 151.5 (42.6-336.4) | 1.89 (1.63-2.15) |
| Liberia | 561.2 (166.4-1023.2) | 410.6 (121.8-748.6) | 614.4 (172.1-1216.2) | 294.8 (82.6-583.5) | -1.54 (-1.69--1.38) |
| Libya | 672.5 (192-1399.5) | 324 (92.5-674.2) | 1741.8 (508.4-3641.6) | 332.5 (97.1-695.2) | 0.01 (-0.22-0.24) |
| Lithuania | 2453.1 (823.1-5103) | 427 (143.3-888.2) | 2208.4 (684.9-4464.2) | 306.4 (95-619.4) | -0.98 (-1.38--0.57) |
| Luxembourg | 233.2 (58.6-496.1) | 331.5 (83.3-705.1) | 139.5 (36.4-285.5) | 116.6 (30.4-238.7) | -3.83 (-3.94--3.72) |
| Madagascar | 1144.8 (328.9-2617.2) | 195.2 (56.1-446.3) | 2155.5 (589.8-5110.2) | 193.2 (52.9-457.9) | -0.3 (-0.55--0.06) |
| Malawi | 746.4 (213.8-1666.4) | 168.5 (48.3-376.2) | 916 (289-2223.2) | 113.3 (35.8-275) | -1.66 (-1.77--1.55) |
| Malaysia | 2934.5 (1080.1-5746.3) | 287 (105.6-562.1) | 5783.3 (2236-10251.3) | 178.9 (69.2-317.1) | -2.41 (-2.68--2.13) |
| Maldives | 76 (24-137.8) | 778.9 (245.4-1411.7) | 104.9 (32.1-188.7) | 337.2 (103.2-606.8) | -3.39 (-3.56--3.22) |
| Mali | 1992.8 (671-3486) | 413.2 (139.1-722.7) | 2055.2 (575.9-4479.7) | 215.8 (60.5-470.4) | -2.58 (-2.93--2.23) |
| Malta | 280.9 (50-619.9) | 517.6 (92.1-1142.2) | 268 (49.4-577.4) | 222 (40.9-478.3) | -3.47 (-3.76--3.19) |
| Marshall Islands | 7.7 (2.2-17.8) | 418.3 (121-964.7) | 13.1 (3.9-30.9) | 353.2 (104.8-830.3) | -0.31 (-0.48--0.13) |
| Mauritania | 570.3 (186.5-1016.5) | 495.7 (162.1-883.5) | 486.9 (132-1035.3) | 202.8 (55-431.2) | -3.08 (-3.39--2.76) |
| Mauritius | 352.5 (109.7-761.6) | 396.4 (123.4-856.6) | 336.1 (97.2-712) | 150.7 (43.6-319.2) | -4.3 (-4.78--3.81) |
| Mexico | 16113.6 (7502.1-25559.6) | 329.1 (153.2-522) | 41339.6 (20224.5-65333.6) | 297.5 (145.6-470.2) | -0.39 (-0.52--0.26) |
| Micronesia (Federated States of) | 21.8 (6.1-51) | 392.2 (109.2-917.1) | 29.2 (8.2-69.1) | 368.9 (104.1-874.7) | -0.19 (-0.25--0.13) |
| Monaco | 48.6 (8.5-109.9) | 539.6 (94.5-1220.6) | 33.9 (6.4-75.3) | 294.1 (55.3-653.2) | -2.43 (-2.73--2.12) |
| Mongolia | 747 (228.2-1600) | 627 (191.5-1343) | 1044.4 (306.4-2224.7) | 446 (130.8-949.9) | -1.92 (-2.47--1.36) |
| Montenegro | 165.3 (52.9-332.8) | 218.1 (69.8-439.2) | 303.9 (99.6-603.9) | 232.3 (76.2-461.5) | 0.42 (0.24-0.6) |
| Morocco | 8603.1 (3139.5-16294.2) | 535.4 (195.4-1014.1) | 11184.8 (4828.4-20651.3) | 304.3 (131.4-561.9) | -2.28 (-2.65--1.92) |
| Mozambique | 1103 (288.6-2428.5) | 164.6 (43.1-362.4) | 1700.6 (497.7-4286.6) | 143.8 (42.1-362.4) | -0.1 (-0.35-0.15) |
| Myanmar | 11228.1 (3211.7-21064.8) | 415.5 (118.9-779.5) | 9726.7 (2660.2-20733.6) | 180.3 (49.3-384.4) | -3.19 (-3.33--3.06) |
| Namibia | 311 (95.4-575.9) | 350 (107.3-648.1) | 560.3 (187.7-1014.5) | 353.8 (118.5-640.5) | -0.03 (-0.25-0.19) |
| Nauru | 1.2 (0.4-2.8) | 374.5 (110.5-857.6) | 1.4 (0.4-3.2) | 403 (123-892.6) | 0.25 (-0.49-0.98) |
| Nepal | 3900.4 (1179.3-6920.1) | 375.1 (113.4-665.6) | 7317.9 (1840.9-15506.5) | 272.2 (68.5-576.9) | -1.21 (-1.38--1.03) |
| Netherlands | 6677.9 (1820.8-13281.1) | 264.2 (72.1-525.5) | 3980.2 (872.7-7565.3) | 93.1 (20.4-176.9) | -4.43 (-4.78--4.08) |
| New Zealand | 1405.4 (467.4-2633.8) | 277.1 (92.2-519.4) | 974.7 (299.8-1796.7) | 99.4 (30.6-183.3) | -4.06 (-4.37--3.75) |
| Nicaragua | 366 (103.6-730.6) | 214.2 (60.6-427.5) | 1020.2 (306.8-2175.6) | 199.7 (60-425.9) | -0.68 (-0.91--0.45) |
| Niger | 1341.2 (427-2396.6) | 437.8 (139.4-782.2) | 1847 (512.6-3986.5) | 215.2 (59.7-464.6) | -2.94 (-3.35--2.52) |
| Nigeria | 29736.3 (14895.6-49699) | 589.3 (295.2-985) | 29191.7 (12992.3-50834.5) | 320.7 (142.7-558.4) | -2.25 (-2.44--2.06) |
| Niue | 0.8 (0.3-1.9) | 300.2 (94.5-698.7) | 0.7 (0.2-1.6) | 270.5 (80.8-596.6) | -0.45 (-0.54--0.35) |
| North Macedonia | 749.5 (220.8-1624) | 331.6 (97.7-718.4) | 1195.6 (300.5-2776) | 281.3 (70.7-653.1) | -0.81 (-0.96--0.66) |
| Northern Mariana Islands | 3 (0.7-7.1) | 201.8 (46.7-485.5) | 14.1 (3.4-34.7) | 229.1 (55-564.2) | 0.8 (0.66-0.95) |
| Norway | 3668.8 (1611.8-5595.6) | 420 (184.5-640.5) | 1327.1 (534.3-2089.1) | 112.6 (45.4-177.3) | -5.02 (-5.2--4.84) |
| Oman | 1172 (452.4-2008) | 1662.7 (641.8-2848.7) | 1135.1 (362.3-2077.7) | 697.5 (222.6-1276.6) | -3.17 (-3.31--3.03) |
| Pakistan | 34506.9 (13063-58404) | 531.7 (201.3-899.9) | 73970 (27559.9-129372.3) | 621 (231.4-1086.2) | 0.63 (0.42-0.85) |
| Palau | 3.5 (1.1-7.7) | 291.1 (90.4-652.5) | 6.5 (1.9-14.8) | 263.8 (75.7-597.8) | -0.4 (-0.46--0.34) |
| Palestine | 1036.5 (353-1835.3) | 1013.6 (345.2-1794.8) | 1819.5 (654.2-3030.6) | 724.5 (260.5-1206.7) | -1.39 (-1.58--1.21) |
| Panama | 445.2 (127.3-852.7) | 256.1 (73.2-490.6) | 628.5 (181.6-1223.5) | 127.3 (36.8-247.9) | -2.12 (-2.31--1.93) |
| Papua New Guinea | 450.4 (121.8-1093.3) | 220.1 (59.5-534.3) | 1565.6 (421-3569.9) | 330.6 (88.9-753.8) | 1.83 (1.55-2.11) |
| Paraguay | 524 (155.4-1058) | 203 (60.2-409.9) | 1170.6 (324.7-2428.4) | 179.1 (49.7-371.6) | -0.24 (-0.52-0.04) |
| Peru | 2493.2 (883.7-4762.4) | 184.1 (65.2-351.6) | 2369.2 (801.6-4403.6) | 62.8 (21.3-116.8) | -3.61 (-4.29--2.94) |
| Philippines | 6247.4 (3357.5-9639.1) | 190.2 (102.2-293.4) | 23375.5 (11788.6-34950.7) | 262.6 (132.4-392.6) | 1.6 (1.22-1.99) |
| Poland | 32406.2 (12761.5-53469.4) | 572.4 (225.4-944.4) | 24683.4 (8539.7-42550.9) | 264.3 (91.4-455.6) | -2.89 (-3.22--2.56) |
| Portugal | 2902.3 (1019.8-5383.3) | 156.9 (55.1-291) | 1974 (595.4-3678.2) | 67.5 (20.4-125.7) | -3.43 (-3.65--3.22) |
| Puerto Rico | 1823 (583.8-3235.9) | 397.6 (127.3-705.7) | 1445.7 (501.9-2640.7) | 160.6 (55.8-293.4) | -3.64 (-3.86--3.43) |
| Qatar | 69.1 (16.8-152.8) | 803.7 (195.7-1776.6) | 271.8 (62.9-629.3) | 318.3 (73.7-737) | -3.2 (-3.76--2.63) |
| Republic of Korea | 5160.3 (1851.9-11093) | 155.4 (55.8-334) | 4155.6 (1555.3-7721.6) | 36.5 (13.7-67.9) | -4.89 (-5.52--4.26) |
| Republic of Moldova | 4813.4 (1411-9085.7) | 863.1 (253-1629.2) | 4242.5 (1236.9-8488.6) | 563.5 (164.3-1127.5) | -1.86 (-2.29--1.43) |
| Romania | 12285.9 (4380.5-22425.7) | 340.1 (121.3-620.9) | 17056.8 (3319.2-33870) | 345.9 (67.3-686.9) | 0.01 (-0.14-0.15) |
| Russian Federation | 146648 (78221.3-223960.3) | 628 (335-959.1) | 132024.2 (67720.3-195118.9) | 423.7 (217.3-626.2) | -1.82 (-2.35--1.28) |
| Rwanda | 796.2 (208.4-1730) | 239.3 (62.7-520) | 875.7 (241.5-2076.7) | 129.5 (35.7-307) | -2.97 (-3.26--2.68) |
| Saint Kitts and Nevis | 23.3 (6.9-46.8) | 446.4 (132.3-897.3) | 18.1 (5.2-34.3) | 223.2 (64.4-422.9) | -2.5 (-2.77--2.23) |
| Saint Lucia | 27.8 (8.2-56.3) | 247.9 (73.3-502.1) | 32.4 (9.6-65.4) | 125.9 (37.2-254.5) | -2.36 (-2.82--1.9) |
| Saint Vincent and the Grenadines | 28.3 (8.9-59.6) | 308 (96.3-648.6) | 31.6 (9.4-64) | 187 (55.9-378.7) | -1.96 (-2.3--1.63) |
| Samoa | 27.9 (9-65.3) | 268.8 (86.9-629.1) | 44.9 (13.3-100.7) | 273.7 (81.3-613.4) | 0.23 (0.15-0.32) |
| San Marino | 7.1 (1.7-15.6) | 165.5 (40.2-363) | 8 (1.9-18.1) | 105.9 (25.6-240.4) | -1.59 (-1.64--1.54) |
| Sao Tome and Principe | 30.4 (10.6-51.8) | 377.5 (131-642.9) | 38 (10.8-74.6) | 337.1 (95.6-661.7) | -0.38 (-0.46--0.3) |
| Saudi Arabia | 2544.5 (686.4-4913.9) | 416.7 (112.4-804.7) | 7656.9 (1392.3-14907.3) | 474.3 (86.2-923.4) | 0.58 (0.36-0.81) |
| Senegal | 2159.6 (1561.4-2818.1) | 579.4 (418.9-756) | 3854.6 (1567.8-6545.5) | 453.6 (184.5-770.2) | -0.82 (-0.95--0.7) |
| Serbia | 4739.2 (1284.3-10344.3) | 321.5 (87.1-701.7) | 5239.3 (1525.6-10898.2) | 239 (69.6-497.1) | -1.22 (-1.37--1.07) |
| Seychelles | 37.1 (13.8-61.4) | 522.3 (193.8-864.9) | 45.2 (16.6-77.2) | 352.6 (129.5-601.7) | -1.45 (-1.68--1.22) |
| Sierra Leone | 1627.2 (740.2-2676.6) | 721.2 (328.1-1186.3) | 1918.1 (670.7-3430.7) | 490.6 (171.6-877.5) | -1.26 (-1.36--1.16) |
| Singapore | 550.2 (212.6-1068.1) | 217.8 (84.2-422.8) | 632.7 (223.1-1087.8) | 64.3 (22.7-110.5) | -4.46 (-4.67--4.25) |
| Slovakia | 3799.2 (1317.3-7002.1) | 488.9 (169.5-901.1) | 3092 (964.2-5972) | 252.7 (78.8-488.1) | -2.17 (-2.63--1.71) |
| Slovenia | 636.2 (180.6-1357.1) | 205.9 (58.4-439.3) | 603.6 (146.8-1321.6) | 111.1 (27-243.1) | -2.53 (-2.69--2.36) |
| Solomon Islands | 136.2 (35.6-285.6) | 931.9 (243.6-1954.2) | 229 (63.7-509.4) | 752 (209.1-1672.9) | -0.8 (-0.96--0.63) |
| Somalia | 563.4 (140.2-1276.6) | 231.6 (57.6-524.7) | 1668.5 (423-3741.1) | 234.5 (59.4-525.7) | 0.09 (0.04-0.13) |
| South Africa | 3412.2 (1508.1-5466) | 141 (62.3-225.9) | 7132 (2814.3-11208) | 135 (53.3-212.1) | -0.1 (-0.51-0.3) |
| South Sudan | 344.4 (100-826.2) | 126.5 (36.7-303.4) | 415.3 (123.3-994.5) | 104.9 (31.2-251.2) | -0.59 (-0.66--0.51) |
| Spain | 12137.5 (3366.4-24296.1) | 169 (46.9-338.2) | 9957.6 (2248.7-19582.9) | 88.7 (20-174.4) | -2.59 (-2.76--2.42) |
| Sri Lanka | 3358.1 (973-7519.2) | 270.8 (78.4-606.2) | 5244.5 (1490.8-11618.1) | 159.5 (45.3-353.3) | -1.68 (-1.87--1.49) |
| Sudan | 8840.9 (2381-17610.9) | 828.5 (223.1-1650.4) | 11394.9 (2999.9-23665.8) | 575 (151.4-1194.2) | -1.44 (-1.5--1.38) |
| Suriname | 84.1 (25.5-173.6) | 280.1 (85-578.4) | 134.5 (40.4-278.3) | 190.6 (57.3-394.2) | -1.41 (-1.79--1.02) |
| Sweden | 6602.3 (2465.5-11355.7) | 345.5 (129-594.2) | 3217 (988.8-5724.7) | 127 (39-226.1) | -3.77 (-3.96--3.58) |
| Switzerland | 2676.2 (887.7-5050.3) | 207.2 (68.7-391) | 1604.5 (489.5-2982.1) | 77.8 (23.7-144.6) | -3.72 (-3.92--3.53) |
| Syrian Arab Republic | 2691.4 (866.7-6301.9) | 458.3 (147.6-1073.1) | 6742.7 (2030.8-14865.5) | 468.1 (141-1032) | -0.24 (-0.42--0.07) |
| Taiwan (Province of China) | 2539.4 (896-4983.7) | 127.4 (45-250.1) | 2525.7 (955-4664.7) | 49.4 (18.7-91.3) | -3.2 (-3.45--2.95) |
| Tajikistan | 2763.2 (974.5-4561.5) | 846.6 (298.6-1397.6) | 5402.2 (1633.1-9716.4) | 1037.8 (313.7-1866.7) | 1.19 (0.72-1.67) |
| Thailand | 4688.5 (1631.8-9273.4) | 118.6 (41.3-234.5) | 8703.8 (2578.2-16805.1) | 68.5 (20.3-132.3) | -1.83 (-2.18--1.48) |
| Timor-Leste | 66.3 (17.6-134.9) | 245.7 (65.2-499.9) | 487.2 (163.2-872.6) | 470.1 (157.5-841.9) | 2.4 (2.31-2.48) |
| Togo | 838 (398.2-1343.4) | 630.3 (299.5-1010.6) | 1355.1 (384.5-2571.1) | 353.7 (100.3-671) | -2.24 (-2.4--2.07) |
| Tokelau | 0.6 (0.2-1.3) | 333.8 (96.9-709.3) | 0.4 (0.1-0.9) | 243.2 (75-539.4) | -1.26 (-1.49--1.02) |
| Tonga | 13.6 (3.9-32) | 207.2 (59.4-488.4) | 17.5 (5.3-41.3) | 189.5 (57.3-447.8) | -0.21 (-0.42--0.01) |
| Trinidad and Tobago | 371.7 (112.1-747.4) | 360.9 (108.9-725.8) | 540.6 (153.7-1120.1) | 227.2 (64.6-470.7) | -2.23 (-2.49--1.97) |
| Tunisia | 1766.4 (559.2-3916.9) | 290.1 (91.8-643.4) | 4086.3 (1097.9-9284.2) | 264.2 (71-600.3) | -0.42 (-0.56--0.29) |
| Turkey | 11755.2 (4183.3-22451) | 284.8 (101.4-544) | 17863.1 (4982.5-34524.7) | 167.4 (46.7-323.5) | -2.02 (-2.48--1.55) |
| Turkmenistan | 1232.6 (380.9-2502) | 554.3 (171.3-1125.2) | 2521.7 (637.1-5870.2) | 586.1 (148.1-1364.4) | -0.65 (-0.98--0.31) |
| Tuvalu | 4.6 (1.2-9.3) | 534.2 (141.5-1096.6) | 4.9 (1.4-10.6) | 386.2 (112.8-836.6) | -0.93 (-0.99--0.87) |
| Uganda | 1130.4 (296.4-2682.1) | 154.2 (40.4-365.8) | 2163.3 (578.4-5021.4) | 146 (39-338.9) | -0.27 (-0.44--0.11) |
| Ukraine | 53581.2 (21803.7-102723.6) | 564.2 (229.6-1081.7) | 71493.8 (31245.3-130924.4) | 711.4 (310.9-1302.8) | -0.17 (-0.85-0.52) |
| United Arab Emirates | 180.3 (37.2-366.6) | 576.6 (118.9-1172.1) | 624.5 (147.6-1262.9) | 233.4 (55.2-472.1) | -3.53 (-4.12--2.94) |
| United Kingdom | 76823.9 (43690.1-109855.7) | 654.8 (372.4-936.3) | 29724.3 (15875.2-42466.4) | 194.9 (104.1-278.4) | -5 (-5.37--4.63) |
| United Republic of Tanzania | 1678.7 (486.5-4058.6) | 133.2 (38.6-321.9) | 3990.5 (1135.8-9545.1) | 150.2 (42.8-359.3) | 0.48 (0.33-0.63) |
| United States of America | 183006 (71227.7-301502.3) | 446.7 (173.9-735.9) | 174953.6 (58628.1-305772) | 245 (82.1-428.2) | -2.66 (-2.9--2.42) |
| United States Virgin Islands | 36.4 (10.7-69.8) | 371.8 (109.4-713.9) | 84.9 (24.7-170.9) | 335.4 (97.5-675) | 0.1 (-0.23-0.43) |
| Uruguay | 1512.9 (369.3-3339.5) | 297.5 (72.6-656.8) | 1340.4 (257-2989.7) | 200.8 (38.5-447.8) | -1.49 (-1.68--1.31) |
| Uzbekistan | 5664.4 (1829.8-12087.8) | 427.1 (138-911.5) | 16132.8 (4955-34767.8) | 724.4 (222.5-1561.1) | 2.07 (1.16-2.99) |
| Vanuatu | 30.9 (8.6-70.5) | 423 (118.1-967) | 84 (24.9-197) | 419.1 (124.1-983) | -0.19 (-0.44-0.07) |
| Venezuela (Bolivarian Republic of) | 2797.8 (956.3-5066.2) | 253.3 (86.6-458.7) | 6379 (2324.3-11464.9) | 180.5 (65.8-324.4) | -1.47 (-1.79--1.15) |
| Viet Nam | 16257.2 (7244-25774.1) | 331 (147.5-524.8) | 17133.7 (6599.3-32014.7) | 160.4 (61.8-299.6) | -2.71 (-2.9--2.53) |
| Yemen | 6470.6 (2182.8-11403.6) | 1181.1 (398.4-2081.5) | 15157.4 (5348.2-25716.3) | 1043.3 (368.1-1770) | -0.92 (-1.09--0.76) |
| Zambia | 758.2 (199.4-1553.4) | 246.6 (64.9-505.3) | 1442.4 (385.5-2886.1) | 210.2 (56.2-420.5) | -0.67 (-1.07--0.26) |
| Zimbabwe | 1390.1 (387.8-2769.9) | 296.9 (82.8-591.5) | 2339.8 (679.3-5034.1) | 299.1 (86.8-643.4) | -0.08 (-0.28-0.12) |

Abbreviations:DALY, Disability-Adjusted Life Years; EAPC, Estimated annual percentage change; SDI, Sociodemographic Index; UI, Uncertainty interval.

**Table S4.** Annual percentage change for cardiovascular diseases deaths overall (net drift) and in each age group (Local drift) by sex from 1990 to 2019 in the old.

| **Age group** | **Both** | **Male** | **Female** |
| --- | --- | --- | --- |
| 60-64 | -1.87 (-2.07, -1.68) | -1.84 (-2.08, -1.61) | -1.95 (-2.13, -1.77) |
| 65-69 | -2.03 (-2.18, -1.89) | -2 (-2.18, -1.81) | -2.17 (-2.3, -2.05) |
| 70-74 | -1.93 (-2.06, -1.8) | -1.88 (-2.04, -1.71) | -2.12 (-2.23, -2.01) |
| 75-79 | -1.76 (-1.88, -1.64) | -1.63 (-1.8, -1.46) | -2.02 (-2.12, -1.93) |
| 80-84 | -1.65 (-1.78, -1.52) | -1.45 (-1.64, -1.26) | -1.96 (-2.06, -1.87) |
| 85-89 | -1.42 (-1.61, -1.24) | -1.12 (-1.4, -0.84) | -1.74 (-1.86, -1.61) |

CI, confidence interval.

**Table S5.** Fitted longitudinal age effects of cardiovascular diseases deaths (per 100 000 person-years) and the corresponding 95% CIs in the old.

| **Age group** | **Both** | **Male** | **Female** |
| --- | --- | --- | --- |
| 60-64 | 6.46 (6.28, 6.64) | 9.05 (8.74, 9.37) | 3.95 (3.86, 4.05) |
| 65-69 | 9.9 (9.64, 10.16) | 13.37 (12.93, 13.83) | 6.7 (6.55, 6.85) |
| 70-74 | 15.48 (15.09, 15.87) | 19.99 (19.33, 20.67) | 11.59 (11.35, 11.84) |
| 75-79 | 24.65 (24.03, 25.28) | 30.71 (29.67, 31.79) | 19.97 (19.57, 20.37) |
| 80-84 | 43.14 (42.09, 44.21) | 52.46 (50.69, 54.29) | 37.03 (36.34, 37.73) |
| 85-89 | 79.26 (77.32, 81.24) | 94.85 (91.48, 98.34) | 71.18 (69.88, 72.51) |

CI, confidence interval.

**Table S6.** Annual percentage change for cardiovascular diseases DALY rate overall (net drift) and in each age group (Local drift) by sex from 1990 to 2019 in the old.

| **Age group** | **Both** | **Male** | **Female** |
| --- | --- | --- | --- |
| 60-64 | -1.81 (-1.95, -1.67) | -1.77 (-1.94, -1.6) | -1.88 (-2.01, -1.75) |
| 65-69 | -1.97 (-2.08, -1.86) | -1.93 (-2.07, -1.8) | -2.1 (-2.2, -2.01) |
| 70-74 | -1.89 (-2, -1.79) | -1.85 (-1.99, -1.71) | -2.07 (-2.16, -1.98) |
| 75-79 | -1.76 (-1.87, -1.64) | -1.65 (-1.81, -1.49) | -2.01 (-2.1, -1.92) |
| 80-84 | -1.68 (-1.82, -1.54) | -1.51 (-1.71, -1.31) | -1.97 (-2.07, -1.87) |
| 85-89 | -1.47 (-1.68, -1.26) | -1.2 (-1.53, -0.87) | -1.77 (-1.92, -1.62) |

CI, confidence interval.

**Table S7.** Fitted longitudinal age effects of cardiovascular diseases DALY rate (per 100 000 person-years) and the corresponding 95% CIs in the old.

| **Age group** | **Both** | **Male** | **Female** |
| --- | --- | --- | --- |
| 60-64 | 185.62 (181.72, 189.6) | 259.99 (253.24, 266.92) | 114.17 (112, 116.38) |
| 65-69 | 240.61 (235.58, 245.76) | 325.29 (316.73, 334.08) | 163.09 (160.12, 166.11) |
| 70-74 | 308.55 (301.88, 315.38) | 399.46 (388.34, 410.88) | 230.96 (226.76, 235.23) |
| 75-79 | 388.63 (379.72, 397.75) | 486.23 (471.51, 501.41) | 313.92 (308.09, 319.86) |
| 80-84 | 520.53 (508.16, 533.19) | 636.24 (615.57, 657.6) | 445.11 (436.82, 453.55) |
| 85-89 | 726.43 (707.44, 745.92) | 874.52 (841.53, 908.81) | 649.95 (637.19, 662.96) |

CI, confidence interval.

**Table S8.** Relative risk for cardiovascular diseases deaths rate of each period compared with the reference (2000–2004) and the corresponding 95% CIs by sex in the old.

| **Periods** | **Both** | **Male** | **Female** |
| --- | --- | --- | --- |
| 1990-1994 | 1.18 (1.15, 1.21) | 1.18 (1.13, 1.22) | 1.2 (1.17, 1.23) |
| 1995-1999 | 1.07 (1.04, 1.09) | 1.06 (1.03, 1.1) | 1.07 (1.05, 1.1) |
| 2000-2004 | 1 (1, 1) | 1 (1, 1) | 1 (1, 1) |
| 2005-2009 | 0.9 (0.88, 0.93) | 0.91 (0.88, 0.94) | 0.89 (0.87, 0.91) |
| 2010-2014 | 0.81 (0.79, 0.83) | 0.83 (0.8, 0.86) | 0.79 (0.77, 0.8) |
| 2015-2019 | 0.76 (0.74, 0.78) | 0.78 (0.75, 0.8) | 0.73 (0.72, 0.75) |

CI, confidence interval.

**Table S9.** Relative risk for cardiovascular diseases DALY rate of each period compared with the reference (2000–2004) and the corresponding 95% CIs by sex in the old.

| **Periods** | **Both** | **Male** | **Female** |
| --- | --- | --- | --- |
| 1990-1994 | 1.16 (1.14, 1.19) | 1.16 (1.12, 1.19) | 1.18 (1.16, 1.21) |
| 1995-1999 | 1.06 (1.04, 1.09) | 1.06 (1.03, 1.09) | 1.07 (1.05, 1.09) |
| 2000-2004 | 1 (1, 1) | 1 (1, 1) | 1 (1, 1) |
| 2005-2009 | 0.9 (0.88, 0.92) | 0.91 (0.88, 0.94) | 0.89 (0.87, 0.91) |
| 2010-2014 | 0.81 (0.79, 0.83) | 0.82 (0.8, 0.85) | 0.78 (0.77, 0.8) |
| 2015-2019 | 0.75 (0.73, 0.77) | 0.76 (0.74, 0.79) | 0.73 (0.71, 0.74) |

CI, confidence interval.

**Table S10.** Relative risk for cardiovascular diseases deaths rate of each birth cohort compared with the reference (cohort 1935-1944) and the corresponding 95% CIs by sex in the old.

| **Cohorts** | **Both** | **Male** | **Female** |
| --- | --- | --- | --- |
| 1900-1909 | 1.75 (1.65, 1.86) | 1.61 (1.46, 1.77) | 1.93 (1.84, 2.01) |
| 1905-1914 | 1.7 (1.63, 1.78) | 1.62 (1.52, 1.72) | 1.85 (1.79, 1.91) |
| 1910-1919 | 1.57 (1.52, 1.63) | 1.52 (1.44, 1.6) | 1.68 (1.63, 1.73) |
| 1915-1924 | 1.43 (1.38, 1.47) | 1.4 (1.34, 1.47) | 1.49 (1.45, 1.53) |
| 1920-1929 | 1.35 (1.31, 1.39) | 1.33 (1.28, 1.39) | 1.4 (1.36, 1.44) |
| 1925-1934 | 1.24 (1.21, 1.28) | 1.24 (1.19, 1.29) | 1.26 (1.23, 1.29) |
| 1930-1939 | 1.11 (1.07, 1.14) | 1.11 (1.07, 1.15) | 1.11 (1.08, 1.14) |
| 1935-1944 | 1 (1, 1) | 1 (1, 1) | 1 (1, 1) |
| 1940-1949 | 0.88 (0.85, 0.92) | 0.88 (0.84, 0.92) | 0.89 (0.86, 0.91) |
| 1945-1954 | 0.82 (0.79, 0.86) | 0.83 (0.78, 0.87) | 0.82 (0.79, 0.85) |
| 1950-1959 | 0.78 (0.74, 0.83) | 0.79 (0.73, 0.85) | 0.78 (0.74, 0.82) |

CI, confidence interval.

**Table S11.** Relative risk for cardiovascular diseases DALY rate of each birth cohort compared with the reference (cohort 1935-1944) and the corresponding 95% CIs by sex in the old.

| **Cohorts** | **Both** | **Male** | **Female** |
| --- | --- | --- | --- |
| 1900-1909 | 1.75 (1.63, 1.88) | 1.62 (1.46, 1.81) | 1.92 (1.83, 2.02) |
| 1905-1914 | 1.71 (1.63, 1.78) | 1.63 (1.53, 1.74) | 1.84 (1.78, 1.9) |
| 1910-1919 | 1.57 (1.52, 1.63) | 1.53 (1.45, 1.6) | 1.67 (1.63, 1.72) |
| 1915-1924 | 1.42 (1.38, 1.46) | 1.4 (1.35, 1.46) | 1.48 (1.45, 1.52) |
| 1920-1929 | 1.34 (1.3, 1.37) | 1.32 (1.28, 1.37) | 1.39 (1.36, 1.42) |
| 1925-1934 | 1.23 (1.2, 1.26) | 1.23 (1.19, 1.26) | 1.25 (1.22, 1.28) |
| 1930-1939 | 1.1 (1.08, 1.13) | 1.1 (1.07, 1.14) | 1.11 (1.08, 1.13) |
| 1935-1944 | 1 (1, 1) | 1 (1, 1) | 1 (1, 1) |
| 1940-1949 | 0.89 (0.86, 0.91) | 0.88 (0.85, 0.91) | 0.89 (0.87, 0.91) |
| 1945-1954 | 0.83 (0.8, 0.86) | 0.83 (0.8, 0.87) | 0.82 (0.8, 0.85) |
| 1950-1959 | 0.79 (0.76, 0.83) | 0.8 (0.76, 0.84) | 0.79 (0.75, 0.82) |

CI, confidence interval.

**Table S12.** Annual percentage change for cardiovascular diseases deaths rate overall (net drift) and in each age group (local drift) by sex and SDI region from 1990 to 2019 in the old.

| **Age group** | **Both** | | | | |
| --- | --- | --- | --- | --- | --- |
|  | **Low SDI** | **Low-middle SDI** | **Middle SDI** | **Middle-high SDI** | **High SDI** |
| 60-64 | -1.03 (-1.26, -0.8) | -0.48 (-0.64, -0.32) | -1.41 (-1.63, -1.19) | -2.78 (-3.18, -2.39) | -2.91 (-3.19, -2.62) |
| 65-69 | -1.02 (-1.2, -0.85) | -0.73 (-0.85, -0.6) | -1.48 (-1.65, -1.31) | -2.74 (-3.03, -2.45) | -3.49 (-3.7, -3.29) |
| 70-74 | -0.9 (-1.06, -0.74) | -0.89 (-1, -0.78) | -1.29 (-1.44, -1.13) | -2.35 (-2.6, -2.09) | -3.82 (-4, -3.64) |
| 75-79 | -0.8 (-0.97, -0.63) | -1.03 (-1.14, -0.91) | -0.9 (-1.05, -0.75) | -1.83 (-2.06, -1.6) | -3.96 (-4.12, -3.8) |
| 80-84 | -0.72 (-0.94, -0.51) | -0.95 (-1.09, -0.81) | -0.45 (-0.62, -0.28) | -1.86 (-2.1, -1.63) | -3.64 (-3.79, -3.48) |
| 85-89 | -0.73 (-1.08, -0.38) | -0.88 (-1.1, -0.66) | 0.16 (-0.1, 0.41) | -1.63 (-1.96, -1.31) | -3.02 (-3.22, -2.83) |
| **Age group** | **Male** | | | | |
|  | **Low SDI** | **Low-middle SDI** | **Middle SDI** | **Middle-high SDI** | **High SDI** |
| 60-64 | -1.03 (-1.31, -0.74) | -0.37 (-0.57, -0.17) | -1.2 (-1.45, -0.95) | -2.76 (-3.2, -2.32) | -2.92 (-3.2, -2.65) |
| 65-69 | -1.03 (-1.25, -0.81) | -0.57 (-0.73, -0.42) | -1.3 (-1.49, -1.1) | -2.7 (-3.04, -2.37) | -3.5 (-3.71, -3.3) |
| 70-74 | -0.9 (-1.1, -0.7) | -0.69 (-0.83, -0.54) | -1.12 (-1.3, -0.94) | -2.24 (-2.55, -1.92) | -3.87 (-4.05, -3.69) |
| 75-79 | -0.76 (-0.98, -0.54) | -0.78 (-0.94, -0.63) | -0.72 (-0.91, -0.54) | -1.59 (-1.89, -1.28) | -3.99 (-4.16, -3.82) |
| 80-84 | -0.63 (-0.92, -0.34) | -0.71 (-0.9, -0.52) | -0.23 (-0.45, -0.01) | -1.55 (-1.89, -1.21) | -3.65 (-3.84, -3.47) |
| 85-89 | -0.52 (-1, -0.04) | -0.66 (-0.97, -0.36) | 0.43 (0.09, 0.78) | -1.22 (-1.72, -0.72) | -2.95 (-3.2, -2.7) |
| **Age group** | **Female** | | | | |
|  | **Low SDI** | **Low-middle SDI** | **Middle SDI** | **Middle-high SDI** | **High SDI** |
| 60-64 | -0.88 (-1.27, -0.49) | -0.53 (-0.79, -0.26) | -1.74 (-1.96, -1.51) | -3.01 (-3.4, -2.62) | -3.07 (-3.47, -2.68) |
| 65-69 | -0.86 (-1.15, -0.56) | -0.84 (-1.04, -0.64) | -1.79 (-1.96, -1.62) | -3.03 (-3.3, -2.76) | -3.76 (-4.04, -3.48) |
| 70-74 | -0.78 (-1.04, -0.52) | -1.08 (-1.26, -0.9) | -1.56 (-1.71, -1.42) | -2.7 (-2.92, -2.48) | -4.07 (-4.29, -3.85) |
| 75-79 | -0.74 (-1.01, -0.48) | -1.25 (-1.42, -1.07) | -1.19 (-1.32, -1.05) | -2.25 (-2.43, -2.07) | -4.22 (-4.4, -4.03) |
| 80-84 | -0.78 (-1.11, -0.45) | -1.16 (-1.37, -0.95) | -0.74 (-0.89, -0.6) | -2.23 (-2.4, -2.06) | -3.87 (-4.03, -3.7) |
| 85-89 | -0.95 (-1.46, -0.43) | -1.04 (-1.36, -0.72) | -0.13 (-0.34, 0.08) | -1.92 (-2.14, -1.69) | -3.25 (-3.44, -3.05) |

SDI, socio-demographic index.

**Table S13.** Fitted longitudinal age effects of cardiovascular diseases deaths (per 100 000 person-years) and the corresponding 95% CIs by sex and SDI region in the old.

| **Age group** | **Both** | | | | |
| --- | --- | --- | --- | --- | --- |
|  | **Low SDI** | **Low-middle SDI** | **Middle SDI** | **Middle-high SDI** | **High SDI** |
| 60-64 | 8.27 (7.99, 8.56) | 6.03 (5.88, 6.18) | 6.25 (6.04, 6.46) | 7.75 (7.35, 8.18) | 4.94 (4.75, 5.14) |
| 65-69 | 13.1 (12.68, 13.53) | 9.51 (9.29, 9.73) | 9.68 (9.39, 9.99) | 11.83 (11.26, 12.44) | 7.05 (6.79, 7.31) |
| 70-74 | 20.6 (19.93, 21.29) | 14.06 (13.73, 14.39) | 15.66 (15.19, 16.14) | 19 (18.12, 19.92) | 10.55 (10.18, 10.93) |
| 75-79 | 30.92 (29.87, 32.02) | 22.27 (21.74, 22.82) | 25.87 (25.09, 26.67) | 31.01 (29.59, 32.48) | 16.56 (16.01, 17.13) |
| 80-84 | 49.21 (47.47, 51.01) | 36.6 (35.71, 37.52) | 48.47 (47.05, 49.93) | 57.47 (54.93, 60.12) | 28.44 (27.56, 29.34) |
| 85-89 | 69.3 (66.5, 72.22) | 56.51 (55.01, 58.05) | 90.96 (88.17, 93.84) | 113.11 (108.1, 118.36) | 58.98 (57.25, 60.77) |
| **Age group** | **Male** | | | | |
|  | **Low SDI** | **Low-middle SDI** | **Middle SDI** | **Middle-high SDI** | **High SDI** |
| 60-64 | 10.96 (10.5, 11.43) | 8.04 (7.79, 8.28) | 8.3 (7.99, 8.62) | 11.73 (11.03, 12.48) | 7.18 (6.9, 7.47) |
| 65-69 | 16.77 (16.09, 17.47) | 12.21 (11.85, 12.57) | 12.48 (12.04, 12.94) | 17.06 (16.07, 18.11) | 10.01 (9.64, 10.4) |
| 70-74 | 25.65 (24.59, 26.75) | 17.34 (16.83, 17.88) | 19.52 (18.83, 20.23) | 25.51 (24.05, 27.05) | 14.49 (13.95, 15.04) |
| 75-79 | 36.66 (35.05, 38.35) | 26.61 (25.78, 27.47) | 31.69 (30.54, 32.88) | 39.16 (36.86, 41.6) | 21.94 (21.14, 22.77) |
| 80-84 | 59.68 (56.95, 62.54) | 43.57 (42.17, 45.01) | 58.09 (55.98, 60.27) | 69.39 (65.29, 73.75) | 36.25 (34.98, 37.56) |
| 85-89 | 83.8 (79.3, 88.56) | 66.86 (64.47, 69.34) | 110.82 (106.51, 115.3) | 134.73 (126.36, 143.66) | 71.74 (69.23, 74.34) |
| **Age group** | **Female** | | | | |
|  | **Low SDI** | **Low-middle SDI** | **Middle SDI** | **Middle-high SDI** | **High SDI** |
| 60-64 | 5.52 (5.21, 5.85) | 4.01 (3.85, 4.18) | 4.16 (4.02, 4.3) | 4.19 (3.98, 4.41) | 2.82 (2.68, 2.97) |
| 65-69 | 9.37 (8.88, 9.88) | 6.84 (6.59, 7.1) | 6.94 (6.74, 7.15) | 7.43 (7.11, 7.76) | 4.41 (4.22, 4.62) |
| 70-74 | 15.59 (14.78, 16.44) | 10.9 (10.5, 11.31) | 12.06 (11.73, 12.41) | 13.92 (13.39, 14.47) | 7.35 (7.05, 7.67) |
| 75-79 | 25.48 (24.12, 26.92) | 18.26 (17.58, 18.96) | 20.89 (20.32, 21.48) | 25.32 (24.42, 26.25) | 12.74 (12.26, 13.24) |
| 80-84 | 39.97 (37.8, 42.28) | 30.52 (29.39, 31.69) | 41.33 (40.26, 42.43) | 50.43 (48.74, 52.17) | 23.89 (23.08, 24.71) |
| 85-89 | 57.71 (54.22, 61.43) | 48.24 (46.33, 50.23) | 79.06 (76.96, 81.21) | 103.31 (99.9, 106.83) | 53.44 (51.77, 55.17) |

SDI, socio-demographic index.

**Table S14.** Annual percentage change for cardiovascular diseases DALY rate overall (net drift) and in each age group (local drift) by sex and SDI region from 1990 to 2019 in the old.

| **Age group** | **Both** | | | | |
| --- | --- | --- | --- | --- | --- |
|  | **Low SDI** | **Low-middle SDI** | **Middle SDI** | **Middle-high SDI** | **High SDI** |
| 60-64 | -1.03 (-1.07, -0.98) | -0.47 (-0.58, -0.36) | -1.38 (-1.53, -1.23) | -2.66 (-2.94, -2.38) | -2.78 (-3, -2.57) |
| 65-69 | -1.01 (-1.05, -0.98) | -0.73 (-0.82, -0.64) | -1.45 (-1.57, -1.33) | -2.63 (-2.85, -2.41) | -3.36 (-3.52, -3.2) |
| 70-74 | -0.9 (-0.93, -0.86) | -0.9 (-0.99, -0.81) | -1.28 (-1.4, -1.16) | -2.28 (-2.49, -2.07) | -3.71 (-3.86, -3.56) |
| 75-79 | -0.8 (-0.85, -0.76) | -1.04 (-1.15, -0.93) | -0.94 (-1.07, -0.81) | -1.84 (-2.05, -1.62) | -3.88 (-4.04, -3.73) |
| 80-84 | -0.73 (-0.79, -0.67) | -0.97 (-1.12, -0.82) | -0.53 (-0.7, -0.35) | -1.92 (-2.17, -1.67) | -3.62 (-3.79, -3.44) |
| 85-89 | -0.74 (-0.85, -0.63) | -0.89 (-1.14, -0.64) | 0.05 (-0.23, 0.34) | -1.72 (-2.1, -1.34) | -3.04 (-3.27, -2.8) |
| **Age group** | **Male** | | | | |
|  | **Low SDI** | **Low-middle SDI** | **Middle SDI** | **Middle-high SDI** | **High SDI** |
| 60-64 | -1.02 (-1.09, -0.96) | -0.35 (-0.47, -0.22) | -1.17 (-1.34, -1) | -2.64 (-2.95, -2.33) | -2.81 (-3.01, -2.6) |
| 65-69 | -1.02 (-1.07, -0.97) | -0.57 (-0.67, -0.46) | -1.26 (-1.4, -1.12) | -2.6 (-2.85, -2.35) | -3.39 (-3.55, -3.23) |
| 70-74 | -0.89 (-0.94, -0.84) | -0.7 (-0.81, -0.6) | -1.12 (-1.26, -0.98) | -2.2 (-2.45, -1.94) | -3.78 (-3.94, -3.63) |
| 75-79 | -0.77 (-0.83, -0.7) | -0.81 (-0.94, -0.68) | -0.77 (-0.94, -0.61) | -1.63 (-1.92, -1.34) | -3.94 (-4.11, -3.78) |
| 80-84 | -0.64 (-0.74, -0.55) | -0.75 (-0.93, -0.57) | -0.33 (-0.55, -0.11) | -1.66 (-2.02, -1.31) | -3.66 (-3.86, -3.46) |
| 85-89 | -0.55 (-0.73, -0.38) | -0.69 (-1, -0.38) | 0.32 (-0.06, 0.7) | -1.36 (-1.94, -0.77) | -2.99 (-3.28, -2.69) |
| **Age group** | **Female** | | | | |
|  | **Low SDI** | **Low-middle SDI** | **Middle SDI** | **Middle-high SDI** | **High SDI** |
| 60-64 | -0.87 (-0.95, -0.79) | -0.55 (-0.68, -0.41) | -1.71 (-1.87, -1.55) | -2.86 (-3.13, -2.58) | -2.91 (-3.21, -2.61) |
| 65-69 | -0.85 (-0.91, -0.79) | -0.85 (-0.96, -0.75) | -1.76 (-1.88, -1.63) | -2.89 (-3.1, -2.69) | -3.58 (-3.81, -3.36) |
| 70-74 | -0.78 (-0.84, -0.72) | -1.08 (-1.19, -0.98) | -1.55 (-1.66, -1.43) | -2.6 (-2.79, -2.42) | -3.91 (-4.11, -3.72) |
| 75-79 | -0.75 (-0.82, -0.68) | -1.24 (-1.36, -1.12) | -1.22 (-1.34, -1.09) | -2.22 (-2.39, -2.05) | -4.11 (-4.29, -3.92) |
| 80-84 | -0.78 (-0.88, -0.69) | -1.15 (-1.31, -0.99) | -0.81 (-0.96, -0.66) | -2.26 (-2.44, -2.07) | -3.83 (-4.02, -3.64) |
| 85-89 | -0.94 (-1.11, -0.78) | -1.04 (-1.3, -0.78) | -0.22 (-0.47, 0.02) | -1.98 (-2.24, -1.71) | -3.27 (-3.51, -3.03) |

SDI, socio-demographic index.

**Table S15.** Fitted longitudinal age effects of cardiovascular diseases DALY rate (per 100 000 person-years) and the corresponding 95% CIs by sex and SDI region in the old.

| **Age group** | **Both** | | | | |
| --- | --- | --- | --- | --- | --- |
|  | **Low SDI** | **Low-middle SDI** | **Middle SDI** | **Middle-high SDI** | **High SDI** |
| 60-64 | 234.58 (232.92, 236.26) | 171.32 (168.26, 174.44) | 180.91 (176.66, 185.26) | 224.25 (215.36, 233.51) | 141.77 (137.4, 146.27) |
| 65-69 | 313.5 (311.26, 315.75) | 228.26 (224.17, 232.41) | 237.01 (231.5, 242.65) | 289.72 (278.3, 301.62) | 171.61 (166.31, 177.08) |
| 70-74 | 404.26 (401.15, 407.39) | 277.24 (271.88, 282.7) | 314.2 (306.58, 322.01) | 381.23 (366.05, 397.05) | 210.91 (204.31, 217.73) |
| 75-79 | 481.64 (477.5, 485.81) | 348.17 (340.79, 355.72) | 410.43 (399.75, 421.4) | 491.52 (470.99, 512.95) | 261.26 (252.92, 269.87) |
| 80-84 | 589.01 (583.38, 594.7) | 439.86 (429.7, 450.25) | 588.18 (572.2, 604.61) | 696.4 (666.3, 727.86) | 342.38 (331.56, 353.56) |
| 85-89 | 631.57 (623.74, 639.5) | 517.21 (502.64, 532.21) | 840.23 (814.2, 867.1) | 1039.96 (990.95, 1091.39) | 538.32 (520.97, 556.25) |
| **Age group** | **Male** | | | | |
|  | **Low SDI** | **Low-middle SDI** | **Middle SDI** | **Middle-high SDI** | **High SDI** |
| 60-64 | 310.96 (307.86, 314.1) | 228.54 (223.94, 233.24) | 240.19 (233.82, 246.74) | 338.81 (323.42, 354.93) | 205.39 (199.08, 211.91) |
| 65-69 | 402.08 (397.99, 406.2) | 293.63 (287.65, 299.75) | 305.53 (297.41, 313.88) | 417.53 (398.21, 437.79) | 242.94 (235.32, 250.82) |
| 70-74 | 505.13 (499.55, 510.77) | 343.32 (335.66, 351.15) | 392.13 (381.06, 403.52) | 512.84 (488.08, 538.86) | 288.92 (279.53, 298.62) |
| 75-79 | 574.25 (567.05, 581.54) | 418.35 (407.97, 428.99) | 504.15 (488.61, 520.18) | 623.72 (590.77, 658.51) | 346.17 (334.38, 358.38) |
| 80-84 | 717.28 (707.16, 727.56) | 526.66 (512.24, 541.49) | 707.08 (683.72, 731.24) | 846.05 (797.73, 897.29) | 437.57 (422.16, 453.54) |
| 85-89 | 768.49 (754.02, 783.24) | 615.05 (593.99, 636.84) | 1026.21 (986.28, 1067.75) | 1247.19 (1164.68, 1335.54) | 658.65 (633.53, 684.76) |
| **Age group** | **Female** | | | | |
|  | **Low SDI** | **Low-middle SDI** | **Middle SDI** | **Middle-high SDI** | **High SDI** |
| 60-64 | 156.23 (154.38, 158.11) | 114.12 (111.72, 116.57) | 120.93 (118.02, 123.92) | 122.08 (117.52, 126.81) | 81.63 (78.29, 85.11) |
| 65-69 | 223 (220.38, 225.66) | 163.76 (160.39, 167.2) | 170.23 (166.27, 174.29) | 182.59 (176.2, 189.21) | 108.52 (104.24, 112.97) |
| 70-74 | 303.82 (300.02, 307.66) | 213.81 (209.15, 218.57) | 242.08 (236.37, 247.93) | 279.17 (269.82, 288.84) | 148.13 (142.44, 154.05) |
| 75-79 | 393.55 (388.21, 398.96) | 283.34 (276.7, 290.15) | 330.95 (322.77, 339.35) | 399.72 (386.36, 413.55) | 201.7 (194.12, 209.57) |
| 80-84 | 475.06 (467.98, 482.24) | 364.17 (355.03, 373.53) | 500.57 (488.02, 513.45) | 607.85 (587.52, 628.88) | 287.66 (277.4, 298.31) |
| 85-89 | 522.14 (512.44, 532.02) | 438.84 (425.8, 452.27) | 729.26 (709.05, 750.04) | 944.65 (911.49, 979.01) | 486.56 (469.52, 504.21) |

SDI, socio-demographic index.

**Table S16.** Relative risk for cardiovascular diseases deaths rate of each period compared with the reference (2000–2004) and the corresponding 95% CIs by sex and SDI region in the old.

| **Periods** | **Both** | | | | |
| --- | --- | --- | --- | --- | --- |
|  | **Low SDI** | **Low-middle SDI** | **Middle SDI** | **Middle-high SDI** | **High SDI** |
| 1990-1994 | 1.07 (1.03, 1.11) | 1.12 (1.09, 1.15) | 1.12 (1.08, 1.16) | 1.13 (1.07, 1.19) | 1.48 (1.43, 1.54) |
| 1995-1999 | 1.04 (1, 1.07) | 1.04 (1.02, 1.07) | 1 (0.97, 1.03) | 1.05 (1, 1.11) | 1.21 (1.17, 1.25) |
| 2000-2004 | 1 (1, 1) | 1 (1, 1) | 1 (1, 1) | 1 (1, 1) | 1 (1, 1) |
| 2005-2009 | 0.95 (0.92, 0.99) | 0.98 (0.96, 1) | 0.98 (0.95, 1.01) | 0.87 (0.83, 0.91) | 0.79 (0.77, 0.82) |
| 2010-2014 | 0.9 (0.87, 0.93) | 0.94 (0.91, 0.96) | 0.93 (0.9, 0.96) | 0.73 (0.69, 0.77) | 0.67 (0.65, 0.7) |
| 2015-2019 | 0.86 (0.83, 0.9) | 0.9 (0.88, 0.92) | 0.86 (0.83, 0.89) | 0.66 (0.63, 0.7) | 0.64 (0.62, 0.67) |
| **Periods** | **Male** | | | | |
|  | **Low SDI** | **Low-middle SDI** | **Middle SDI** | **Middle-high SDI** | **High SDI** |
| 1990-1994 | 1.06 (1.01, 1.11) | 1.09 (1.06, 1.13) | 1.11 (1.06, 1.15) | 1.1 (1.03, 1.18) | 1.51 (1.46, 1.57) |
| 1995-1999 | 1.03 (0.99, 1.08) | 1.03 (1, 1.07) | 1 (0.96, 1.04) | 1.04 (0.97, 1.1) | 1.23 (1.18, 1.27) |
| 2000-2004 | 1 (1, 1) | 1 (1, 1) | 1 (1, 1) | 1 (1, 1) | 1 (1, 1) |
| 2005-2009 | 0.96 (0.92, 1) | 1 (0.97, 1.03) | 0.99 (0.95, 1.02) | 0.88 (0.82, 0.93) | 0.8 (0.77, 0.83) |
| 2010-2014 | 0.91 (0.87, 0.95) | 0.96 (0.93, 0.99) | 0.96 (0.92, 0.99) | 0.74 (0.7, 0.79) | 0.68 (0.65, 0.71) |
| 2015-2019 | 0.87 (0.83, 0.91) | 0.92 (0.89, 0.95) | 0.9 (0.86, 0.93) | 0.68 (0.64, 0.72) | 0.65 (0.63, 0.68) |
| **Periods** | **Female** | | | | |
|  | **Low SDI** | **Low-middle SDI** | **Middle SDI** | **Middle-high SDI** | **High SDI** |
| 1990-1994 | 1.06 (1, 1.13) | 1.15 (1.1, 1.2) | 1.15 (1.11, 1.19) | 1.19 (1.14, 1.24) | 1.49 (1.43, 1.56) |
| 1995-1999 | 1.03 (0.97, 1.09) | 1.05 (1, 1.09) | 1 (0.97, 1.03) | 1.09 (1.04, 1.13) | 1.21 (1.17, 1.26) |
| 2000-2004 | 1 (1, 1) | 1 (1, 1) | 1 (1, 1) | 1 (1, 1) | 1 (1, 1) |
| 2005-2009 | 0.95 (0.9, 1.01) | 0.96 (0.92, 1) | 0.96 (0.93, 0.99) | 0.85 (0.82, 0.89) | 0.78 (0.75, 0.81) |
| 2010-2014 | 0.9 (0.85, 0.95) | 0.92 (0.88, 0.95) | 0.89 (0.86, 0.91) | 0.71 (0.68, 0.74) | 0.65 (0.62, 0.67) |
| 2015-2019 | 0.87 (0.82, 0.92) | 0.89 (0.86, 0.93) | 0.82 (0.79, 0.84) | 0.65 (0.62, 0.67) | 0.61 (0.59, 0.64) |

CI, confidence interval; SDI, socio-demographic index.

**Table S17.** Relative risk for cardiovascular diseases DALY rate of each period compared with the reference (2000–2004) and the corresponding 95% CIs by sex and SDI region in the old.

| **Periods** | **Both** | | | | |
| --- | --- | --- | --- | --- | --- |
|  | **Low SDI** | **Low-middle SDI** | **Middle SDI** | **Middle-high SDI** | **High SDI** |
| 1990-1994 | 1.07 (1.06, 1.08) | 1.12 (1.1, 1.15) | 1.11 (1.08, 1.14) | 1.1 (1.05, 1.15) | 1.45 (1.41, 1.5) |
| 1995-1999 | 1.04 (1.03, 1.05) | 1.04 (1.02, 1.07) | 1 (0.97, 1.03) | 1.04 (1, 1.09) | 1.2 (1.17, 1.24) |
| 2000-2004 | 1 (1, 1) | 1 (1, 1) | 1 (1, 1) | 1 (1, 1) | 1 (1, 1) |
| 2005-2009 | 0.95 (0.94, 0.96) | 0.98 (0.96, 1) | 0.97 (0.95, 0.99) | 0.86 (0.83, 0.9) | 0.8 (0.77, 0.83) |
| 2010-2014 | 0.9 (0.89, 0.91) | 0.94 (0.92, 0.96) | 0.92 (0.89, 0.94) | 0.72 (0.69, 0.75) | 0.67 (0.65, 0.7) |
| 2015-2019 | 0.86 (0.86, 0.87) | 0.9 (0.88, 0.92) | 0.85 (0.83, 0.87) | 0.65 (0.62, 0.68) | 0.64 (0.62, 0.66) |
| **Periods** | **Male** | | | | |
|  | **Low SDI** | **Low-middle SDI** | **Middle SDI** | **Middle-high SDI** | **High SDI** |
| 1990-1994 | 1.06 (1.04, 1.07) | 1.09 (1.07, 1.12) | 1.09 (1.06, 1.13) | 1.07 (1.01, 1.13) | 1.48 (1.43, 1.53) |
| 1995-1999 | 1.03 (1.02, 1.05) | 1.03 (1.01, 1.06) | 1 (0.97, 1.03) | 1.02 (0.97, 1.08) | 1.22 (1.18, 1.26) |
| 2000-2004 | 1 (1, 1) | 1 (1, 1) | 1 (1, 1) | 1 (1, 1) | 1 (1, 1) |
| 2005-2009 | 0.95 (0.94, 0.97) | 1 (0.98, 1.02) | 0.98 (0.95, 1.01) | 0.87 (0.82, 0.91) | 0.8 (0.77, 0.83) |
| 2010-2014 | 0.91 (0.9, 0.92) | 0.96 (0.94, 0.98) | 0.94 (0.92, 0.97) | 0.73 (0.69, 0.77) | 0.68 (0.66, 0.71) |
| 2015-2019 | 0.87 (0.85, 0.88) | 0.91 (0.89, 0.93) | 0.88 (0.85, 0.91) | 0.66 (0.62, 0.7) | 0.65 (0.62, 0.67) |
| **Periods** | **Female** | | | | |
|  | **Low SDI** | **Low-middle SDI** | **Middle SDI** | **Middle-high SDI** | **High SDI** |
| 1990-1994 | 1.06 (1.05, 1.08) | 1.16 (1.13, 1.19) | 1.13 (1.1, 1.16) | 1.16 (1.12, 1.2) | 1.45 (1.4, 1.51) |
| 1995-1999 | 1.03 (1.01, 1.04) | 1.05 (1.03, 1.08) | 1 (0.97, 1.03) | 1.07 (1.03, 1.11) | 1.2 (1.16, 1.25) |
| 2000-2004 | 1 (1, 1) | 1 (1, 1) | 1 (1, 1) | 1 (1, 1) | 1 (1, 1) |
| 2005-2009 | 0.95 (0.94, 0.97) | 0.96 (0.94, 0.98) | 0.95 (0.93, 0.98) | 0.85 (0.82, 0.88) | 0.78 (0.75, 0.82) |
| 2010-2014 | 0.9 (0.89, 0.91) | 0.92 (0.9, 0.94) | 0.88 (0.85, 0.9) | 0.71 (0.68, 0.73) | 0.65 (0.62, 0.68) |
| 2015-2019 | 0.87 (0.86, 0.88) | 0.89 (0.87, 0.92) | 0.81 (0.79, 0.83) | 0.64 (0.62, 0.67) | 0.61 (0.59, 0.64) |

CI, confidence interval; SDI, socio-demographic index.

**Table S18.** Relative risk for cardiovascular diseases deaths rate of each birth cohort compared with the reference (cohort 1935-1944) and the corresponding 95% CIs by sex and SDI region in the old.

| **Cohorts** | **Both** | | | | |
| --- | --- | --- | --- | --- | --- |
|  | **Low SDI** | **Low-middle SDI** | **Middle SDI** | **Middle-high SDI** | **High SDI** |
| 1900-1909 | 1.33 (1.18, 1.49) | 1.37 (1.27, 1.47) | 1.12 (1.03, 1.23) | 1.87 (1.67, 2.09) | 3.22 (3, 3.46) |
| 1905-1914 | 1.26 (1.17, 1.35) | 1.31 (1.25, 1.37) | 1.24 (1.17, 1.31) | 1.84 (1.69, 1.99) | 3 (2.84, 3.18) |
| 1910-1919 | 1.22 (1.16, 1.29) | 1.29 (1.24, 1.34) | 1.25 (1.2, 1.31) | 1.62 (1.51, 1.73) | 2.69 (2.56, 2.83) |
| 1915-1924 | 1.19 (1.14, 1.24) | 1.23 (1.19, 1.27) | 1.26 (1.21, 1.31) | 1.41 (1.32, 1.51) | 2.27 (2.17, 2.38) |
| 1920-1929 | 1.15 (1.1, 1.19) | 1.15 (1.12, 1.18) | 1.24 (1.2, 1.29) | 1.39 (1.31, 1.47) | 1.88 (1.79, 1.96) |
| 1925-1934 | 1.09 (1.06, 1.13) | 1.09 (1.07, 1.12) | 1.19 (1.15, 1.23) | 1.28 (1.21, 1.35) | 1.51 (1.44, 1.58) |
| 1930-1939 | 1.05 (1.01, 1.09) | 1.04 (1.02, 1.07) | 1.1 (1.06, 1.14) | 1.1 (1.04, 1.17) | 1.21 (1.15, 1.27) |
| 1935-1944 | 1 (1, 1) | 1 (1, 1) | 1 (1, 1) | 1 (1, 1) | 1 (1, 1) |
| 1940-1949 | 0.95 (0.91, 0.99) | 0.99 (0.96, 1.01) | 0.92 (0.89, 0.96) | 0.77 (0.72, 0.83) | 0.89 (0.84, 0.94) |
| 1945-1954 | 0.88 (0.84, 0.93) | 0.96 (0.93, 0.99) | 0.87 (0.83, 0.92) | 0.72 (0.66, 0.79) | 0.77 (0.72, 0.83) |
| 1950-1959 | 0.85 (0.8, 0.91) | 0.98 (0.93, 1.03) | 0.84 (0.79, 0.9) | 0.64 (0.57, 0.73) | 0.72 (0.66, 0.79) |
| **Cohorts** | **Male** | | | | |
|  | **Low SDI** | **Low-middle SDI** | **Middle SDI** | **Middle-high SDI** | **High SDI** |
| 1900-1909 | 1.25 (1.07, 1.47) | 1.26 (1.14, 1.39) | 1.03 (0.92, 1.15) | 1.67 (1.41, 1.98) | 3.15 (2.89, 3.44) |
| 1905-1914 | 1.23 (1.12, 1.34) | 1.22 (1.15, 1.3) | 1.17 (1.09, 1.25) | 1.7 (1.52, 1.9) | 3 (2.81, 3.19) |
| 1910-1919 | 1.2 (1.12, 1.29) | 1.22 (1.16, 1.28) | 1.2 (1.14, 1.27) | 1.52 (1.39, 1.67) | 2.7 (2.56, 2.85) |
| 1915-1924 | 1.18 (1.12, 1.25) | 1.17 (1.13, 1.22) | 1.22 (1.16, 1.28) | 1.35 (1.25, 1.47) | 2.3 (2.19, 2.42) |
| 1920-1929 | 1.15 (1.09, 1.2) | 1.1 (1.07, 1.15) | 1.21 (1.16, 1.27) | 1.36 (1.26, 1.46) | 1.88 (1.79, 1.97) |
| 1925-1934 | 1.09 (1.04, 1.14) | 1.07 (1.04, 1.11) | 1.17 (1.12, 1.21) | 1.27 (1.19, 1.36) | 1.51 (1.44, 1.58) |
| 1930-1939 | 1.05 (1, 1.1) | 1.04 (1.01, 1.08) | 1.1 (1.05, 1.14) | 1.1 (1.02, 1.18) | 1.2 (1.15, 1.26) |
| 1935-1944 | 1 (1, 1) | 1 (1, 1) | 1 (1, 1) | 1 (1, 1) | 1 (1, 1) |
| 1940-1949 | 0.94 (0.89, 0.99) | 0.98 (0.95, 1.02) | 0.93 (0.89, 0.98) | 0.76 (0.7, 0.83) | 0.88 (0.83, 0.93) |
| 1945-1954 | 0.88 (0.83, 0.94) | 0.96 (0.92, 1) | 0.89 (0.85, 0.95) | 0.72 (0.65, 0.8) | 0.77 (0.72, 0.82) |
| 1950-1959 | 0.86 (0.78, 0.93) | 0.99 (0.93, 1.05) | 0.87 (0.81, 0.94) | 0.65 (0.56, 0.74) | 0.71 (0.65, 0.78) |
| **Cohorts** | **Female** | | | | |
|  | **Low SDI** | **Low-middle SDI** | **Middle SDI** | **Middle-high SDI** | **High SDI** |
| 1900-1909 | 1.38 (1.17, 1.63) | 1.45 (1.31, 1.62) | 1.24 (1.16, 1.33) | 2.08 (1.92, 2.26) | 3.53 (3.27, 3.81) |
| 1905-1914 | 1.27 (1.14, 1.41) | 1.38 (1.28, 1.48) | 1.35 (1.28, 1.41) | 2.03 (1.91, 2.15) | 3.25 (3.04, 3.46) |
| 1910-1919 | 1.2 (1.11, 1.31) | 1.35 (1.28, 1.43) | 1.34 (1.29, 1.4) | 1.78 (1.68, 1.88) | 2.88 (2.72, 3.06) |
| 1915-1924 | 1.16 (1.08, 1.25) | 1.28 (1.22, 1.35) | 1.33 (1.28, 1.38) | 1.54 (1.46, 1.62) | 2.4 (2.27, 2.54) |
| 1920-1929 | 1.12 (1.05, 1.19) | 1.19 (1.14, 1.24) | 1.3 (1.25, 1.34) | 1.48 (1.41, 1.56) | 1.97 (1.87, 2.09) |
| 1925-1934 | 1.07 (1.01, 1.14) | 1.11 (1.07, 1.16) | 1.21 (1.18, 1.25) | 1.31 (1.25, 1.38) | 1.55 (1.47, 1.64) |
| 1930-1939 | 1.04 (0.98, 1.1) | 1.05 (1.01, 1.09) | 1.11 (1.07, 1.14) | 1.11 (1.06, 1.17) | 1.23 (1.16, 1.31) |
| 1935-1944 | 1 (1, 1) | 1 (1, 1) | 1 (1, 1) | 1 (1, 1) | 1 (1, 1) |
| 1940-1949 | 0.95 (0.89, 1.02) | 0.99 (0.94, 1.03) | 0.91 (0.87, 0.95) | 0.77 (0.72, 0.83) | 0.88 (0.82, 0.95) |
| 1945-1954 | 0.9 (0.83, 0.98) | 0.96 (0.91, 1.02) | 0.84 (0.8, 0.88) | 0.71 (0.65, 0.77) | 0.75 (0.69, 0.83) |
| 1950-1959 | 0.87 (0.77, 0.98) | 0.98 (0.9, 1.06) | 0.79 (0.74, 0.85) | 0.62 (0.55, 0.7) | 0.72 (0.63, 0.82) |

CI, confidence interval; SDI, socio-demographic index.

**Table S19.** Relative risk for cardiovascular diseases DALY rate of each birth cohort compared with the reference (cohort 1935-1944) and the corresponding 95% CIs by sex and SDI region in the old.

| **Cohorts** | **Both** | | | | |
| --- | --- | --- | --- | --- | --- |
|  | **Low SDI** | **Low-middle SDI** | **Middle SDI** | **Middle-high SDI** | **High SDI** |
| 1900-1909 | 1.33 (1.28, 1.38) | 1.37 (1.26, 1.49) | 1.15 (1.04, 1.26) | 1.88 (1.65, 2.13) | 3.17 (2.93, 3.44) |
| 1905-1914 | 1.26 (1.24, 1.29) | 1.31 (1.25, 1.37) | 1.26 (1.19, 1.33) | 1.84 (1.69, 2) | 2.95 (2.79, 3.13) |
| 1910-1919 | 1.22 (1.21, 1.24) | 1.29 (1.25, 1.34) | 1.27 (1.22, 1.32) | 1.62 (1.51, 1.73) | 2.64 (2.51, 2.77) |
| 1915-1924 | 1.19 (1.17, 1.2) | 1.23 (1.2, 1.26) | 1.26 (1.22, 1.3) | 1.4 (1.32, 1.49) | 2.23 (2.13, 2.32) |
| 1920-1929 | 1.15 (1.14, 1.16) | 1.15 (1.12, 1.18) | 1.24 (1.2, 1.27) | 1.37 (1.3, 1.44) | 1.83 (1.76, 1.91) |
| 1925-1934 | 1.1 (1.09, 1.1) | 1.1 (1.07, 1.12) | 1.18 (1.15, 1.21) | 1.26 (1.2, 1.32) | 1.48 (1.43, 1.54) |
| 1930-1939 | 1.05 (1.04, 1.06) | 1.04 (1.02, 1.06) | 1.1 (1.07, 1.13) | 1.09 (1.04, 1.14) | 1.19 (1.15, 1.24) |
| 1935-1944 | 1 (1, 1) | 1 (1, 1) | 1 (1, 1) | 1 (1, 1) | 1 (1, 1) |
| 1940-1949 | 0.95 (0.94, 0.96) | 0.98 (0.96, 1.01) | 0.93 (0.9, 0.95) | 0.78 (0.73, 0.82) | 0.89 (0.85, 0.93) |
| 1945-1954 | 0.88 (0.87, 0.89) | 0.96 (0.93, 0.98) | 0.88 (0.85, 0.91) | 0.73 (0.68, 0.78) | 0.78 (0.74, 0.82) |
| 1950-1959 | 0.85 (0.84, 0.87) | 0.98 (0.95, 1.01) | 0.85 (0.81, 0.89) | 0.65 (0.6, 0.71) | 0.73 (0.68, 0.78) |
| **Cohorts** | **Male** | | | | |
|  | **Low SDI** | **Low-middle SDI** | **Middle SDI** | **Middle-high SDI** | **High SDI** |
| 1900-1909 | 1.26 (1.19, 1.34) | 1.27 (1.15, 1.4) | 1.05 (0.93, 1.19) | 1.69 (1.39, 2.05) | 3.13 (2.83, 3.45) |
| 1905-1914 | 1.23 (1.19, 1.27) | 1.23 (1.16, 1.3) | 1.19 (1.11, 1.28) | 1.72 (1.53, 1.93) | 2.97 (2.78, 3.17) |
| 1910-1919 | 1.21 (1.19, 1.23) | 1.23 (1.18, 1.28) | 1.22 (1.16, 1.28) | 1.54 (1.41, 1.68) | 2.66 (2.53, 2.8) |
| 1915-1924 | 1.18 (1.16, 1.2) | 1.18 (1.14, 1.21) | 1.22 (1.17, 1.27) | 1.35 (1.26, 1.46) | 2.27 (2.17, 2.37) |
| 1920-1929 | 1.14 (1.13, 1.16) | 1.1 (1.08, 1.13) | 1.21 (1.17, 1.25) | 1.34 (1.26, 1.43) | 1.85 (1.77, 1.92) |
| 1925-1934 | 1.09 (1.08, 1.1) | 1.07 (1.04, 1.09) | 1.16 (1.13, 1.2) | 1.25 (1.18, 1.32) | 1.49 (1.43, 1.54) |
| 1930-1939 | 1.05 (1.04, 1.06) | 1.04 (1.02, 1.06) | 1.1 (1.06, 1.13) | 1.09 (1.03, 1.15) | 1.19 (1.15, 1.24) |
| 1935-1944 | 1 (1, 1) | 1 (1, 1) | 1 (1, 1) | 1 (1, 1) | 1 (1, 1) |
| 1940-1949 | 0.94 (0.93, 0.95) | 0.98 (0.96, 1.01) | 0.94 (0.9, 0.97) | 0.76 (0.71, 0.82) | 0.89 (0.85, 0.93) |
| 1945-1954 | 0.88 (0.87, 0.89) | 0.96 (0.93, 0.99) | 0.9 (0.86, 0.93) | 0.73 (0.68, 0.79) | 0.78 (0.74, 0.82) |
| 1950-1959 | 0.86 (0.84, 0.87) | 1 (0.96, 1.03) | 0.88 (0.84, 0.93) | 0.66 (0.59, 0.72) | 0.72 (0.68, 0.77) |
| **Cohorts** | **Female** | | | | |
|  | **Low SDI** | **Low-middle SDI** | **Middle SDI** | **Middle-high SDI** | **High SDI** |
| 1900-1909 | 1.38 (1.31, 1.46) | 1.46 (1.34, 1.59) | 1.26 (1.16, 1.37) | 2.07 (1.9, 2.27) | 3.46 (3.17, 3.76) |
| 1905-1914 | 1.27 (1.23, 1.31) | 1.38 (1.31, 1.45) | 1.37 (1.3, 1.44) | 2.01 (1.89, 2.14) | 3.17 (2.97, 3.38) |
| 1910-1919 | 1.21 (1.18, 1.23) | 1.35 (1.31, 1.4) | 1.36 (1.31, 1.41) | 1.77 (1.68, 1.86) | 2.8 (2.64, 2.96) |
| 1915-1924 | 1.16 (1.14, 1.18) | 1.28 (1.24, 1.32) | 1.33 (1.28, 1.37) | 1.52 (1.45, 1.59) | 2.33 (2.21, 2.46) |
| 1920-1929 | 1.12 (1.1, 1.14) | 1.19 (1.16, 1.22) | 1.29 (1.25, 1.33) | 1.46 (1.4, 1.52) | 1.91 (1.82, 2.01) |
| 1925-1934 | 1.07 (1.06, 1.09) | 1.12 (1.09, 1.14) | 1.21 (1.18, 1.24) | 1.29 (1.24, 1.35) | 1.52 (1.44, 1.59) |
| 1930-1939 | 1.04 (1.03, 1.05) | 1.05 (1.02, 1.07) | 1.11 (1.08, 1.14) | 1.1 (1.06, 1.15) | 1.21 (1.15, 1.28) |
| 1935-1944 | 1 (1, 1) | 1 (1, 1) | 1 (1, 1) | 1 (1, 1) | 1 (1, 1) |
| 1940-1949 | 0.95 (0.94, 0.97) | 0.98 (0.96, 1.01) | 0.91 (0.88, 0.94) | 0.78 (0.74, 0.82) | 0.89 (0.84, 0.95) |
| 1945-1954 | 0.9 (0.88, 0.91) | 0.96 (0.93, 0.99) | 0.84 (0.81, 0.87) | 0.72 (0.68, 0.77) | 0.76 (0.71, 0.82) |
| 1950-1959 | 0.87 (0.85, 0.89) | 0.97 (0.94, 1.01) | 0.8 (0.76, 0.84) | 0.64 (0.58, 0.7) | 0.73 (0.66, 0.8) |

CI, confidence interval; SDI, socio-demographic index.


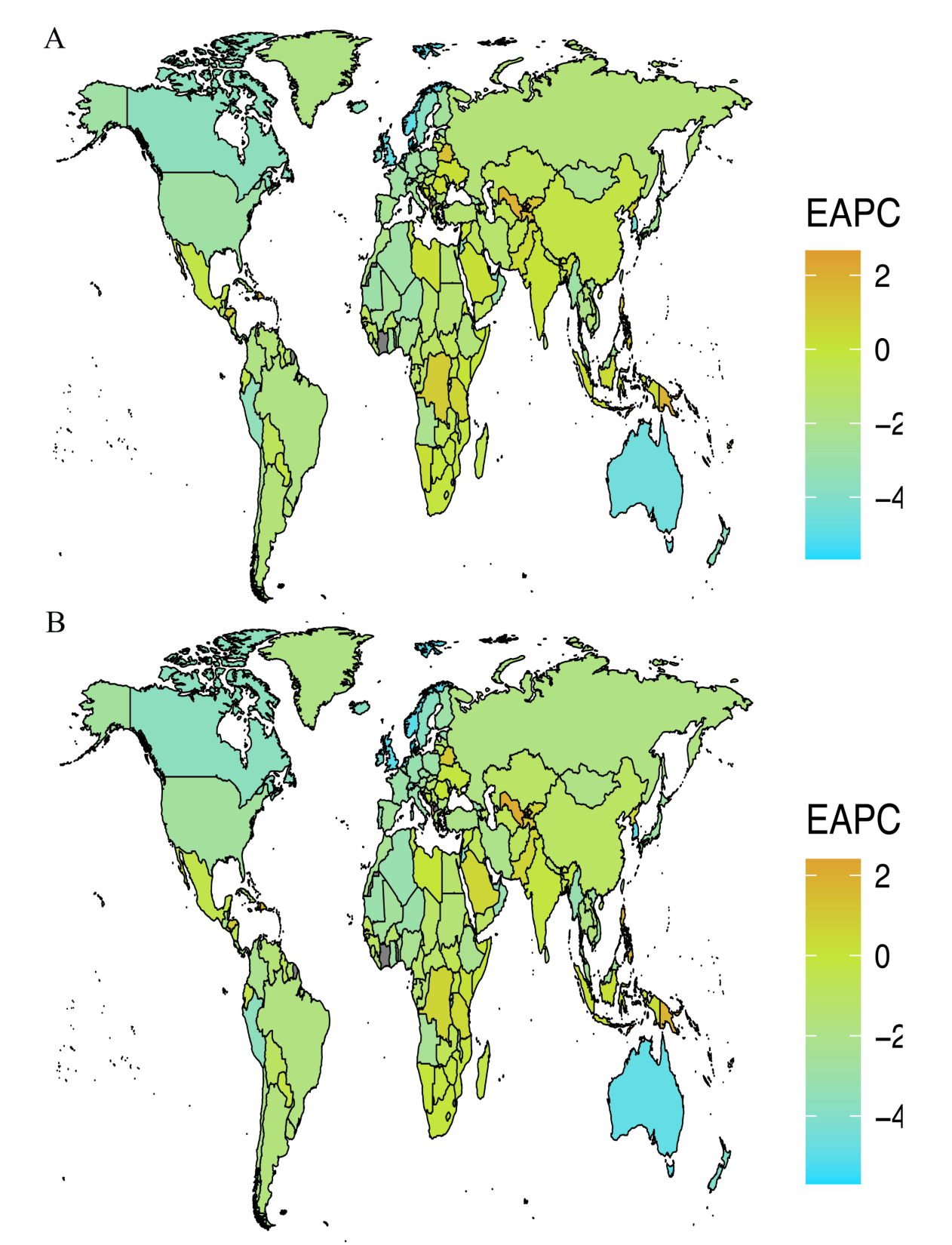


**Figure S1.** Global Burden of disease for CVD in women and men for 204 countries and territories (A) EAPC for CVD mortality; (B) EAPC of CVD DALY.


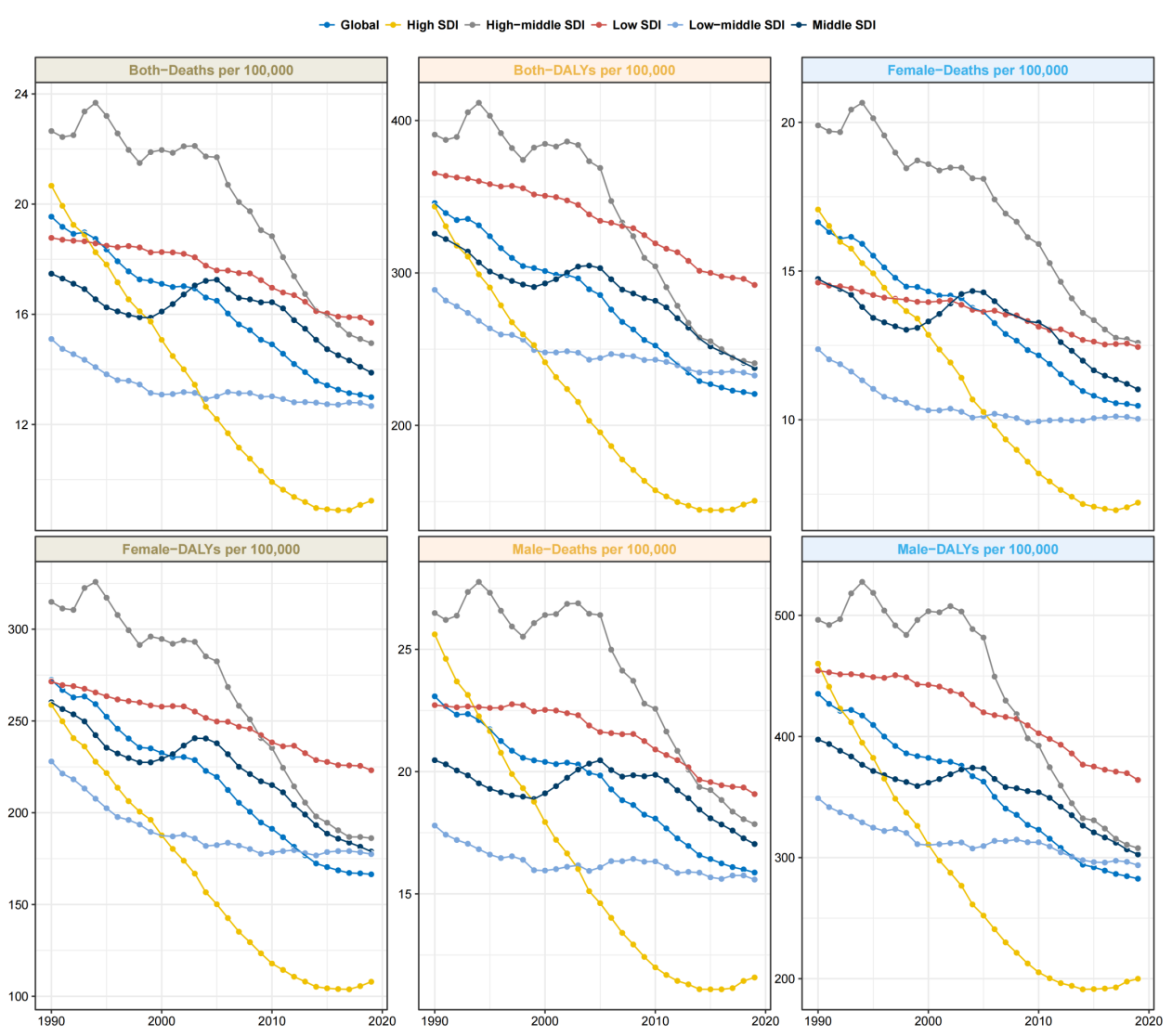


**Figure S2.** Trends of ASMR and ASDR of CVD in regions with different development levels from 1990 to 2019.


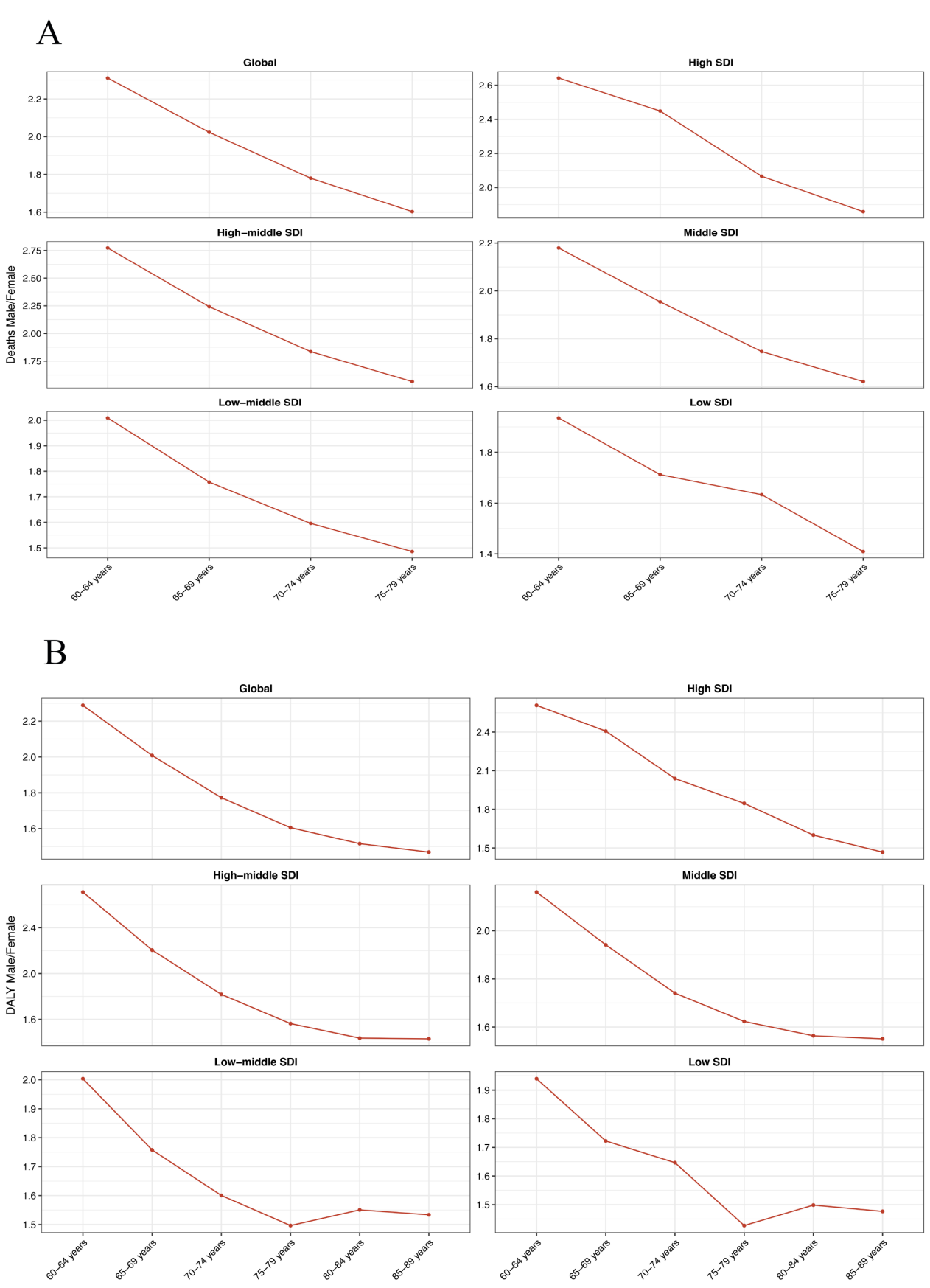


**Figure S3.** Mortality and disability-adjusted life years (DALY) ratio of men to women for bipolar disorder by age subgroup (A) mortality ratio of men to women; (B) The proportion of DALYs among men and women.


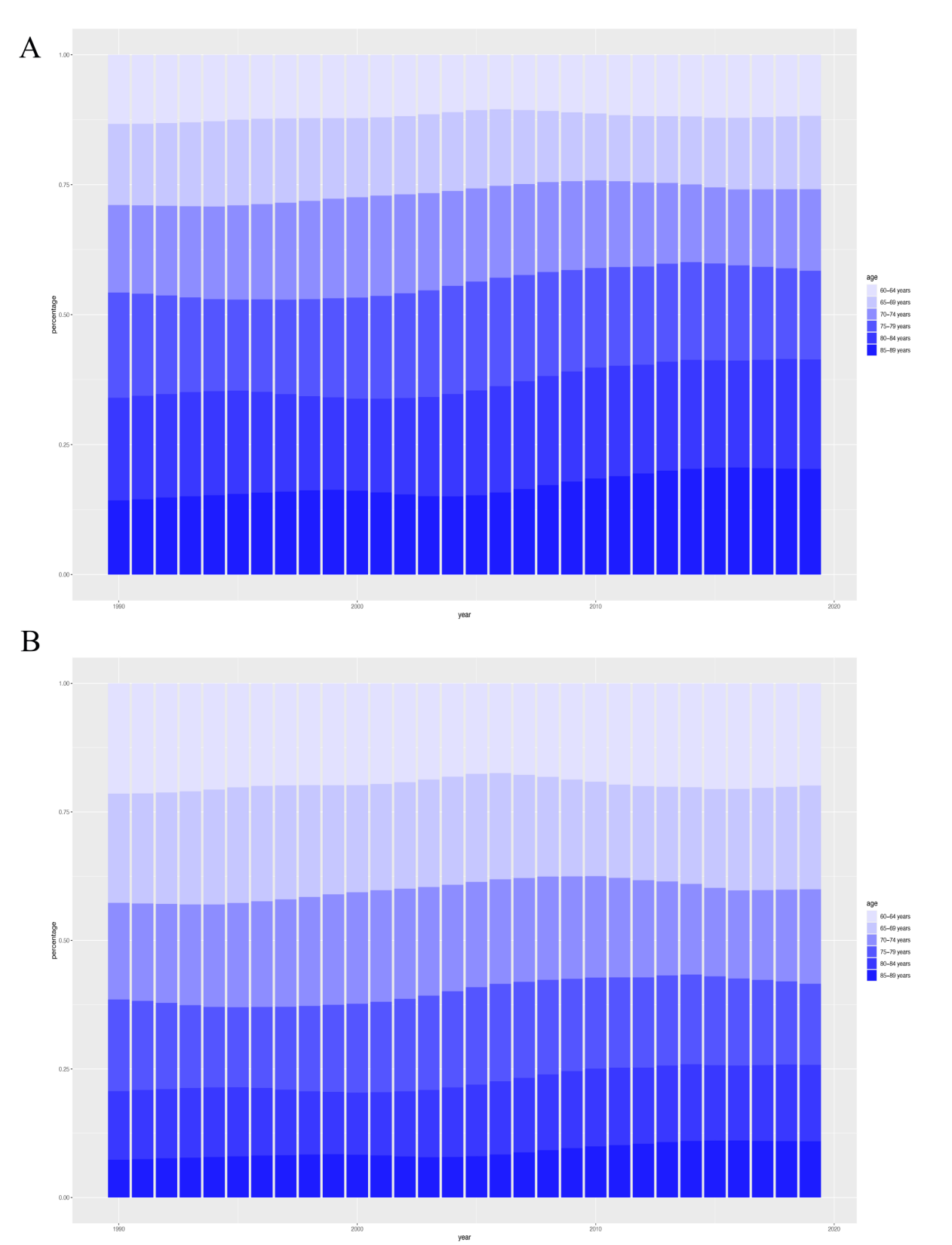


**Figure S4.** Temporal changes in the age distribution of CVD mortality and DALY among people aged 60 and above from 1990 to 2019.

**Figure S5.** The various ways of reducing CVD mortality due to SSBs.
